# Comparing *Campylobacter jejuni* to three other enteric pathogens in OligoMM^12^ mice reveals pathogen-specific host and microbiota responses

**DOI:** 10.1080/19490976.2024.2447832

**Published:** 2025-01-21

**Authors:** Mathias K.-M. Herzog, Audrey Peters, Nizar Shayya, Monica Cazzaniga, Kardokh Kaka Bra, Trisha Arora, Manja Barthel, Ersin Gül, Luca Maurer, Patrick Kiefer, Philipp Christen, Katharina Endhardt, Julia A. Vorholt, Gad Frankel, Markus M. Heimesaat, Stefan Bereswill, Cormac G. M. Gahan, Marcus J. Claesson, Xavier Domingo-Almenara, Wolf-Dietrich Hardt

**Affiliations:** a Institute of Microbiology, Department of Biology, ETH Zurich, Zurich, Switzerland; b Department of Life Sciences, MRC Centre for Bacterial Resistance Biology, Imperial College London, London, UK; c Gastrointestinal Microbiology Research Group, Institute of Microbiology, Infectious Diseases and Immunology, Charité-Universitätsmedizin Berlin, Corporate Member of Freie Universität Berlin, Humboldt-Universität zu Berlin, and Berlin Institute of Health, Berlin, Germany; d APC Microbiome Ireland, University College Cork, Cork, Ireland; e School of Microbiology, University College Cork, Cork, Ireland; f Omic Sciences Unit, EURECAT – Technology Centre of Catalonia, Reus, Spain; g Department of Pathology and Molecular Pathology, University Hospital Zurich, Zurich, Switzerland; h School of Pharmacy, University College Cork, Cork, Ireland

**Keywords:** Infectious diseases, mouse model, microbiota, *Campylobacter jejuni*, *Listeria monocytogenes*, *Citrobacter rodentium*, *Salmonella* Typhimurium

## Abstract

*Campylobacter jejuni*, non-typhoidal *Salmonella* spp., *Listeria monocytogenes* and enteropathogenic/enterohemorrhagic *Escherichia coli* (EPEC/EHEC) are leading causes of food-borne illness worldwide. *Citrobacter rodentium* has been used to model EPEC and EHEC infection in mice. The gut microbiome is well-known to affect gut colonization and host responses to many food-borne pathogens. Recent progress has established gnotobiotic mice as valuable models to study how microbiota affect the enteric infections by *S*. Typhimurium, *C. rodentium* and *L. monocytogenes*. However, for *C. jejuni*, we are still lacking a suitable gnotobiotic mouse model. Moreover, the limited comparability of data across laboratories is often negatively affected by variations between different research facilities or murine microbiotas. In this study, we applied the standardized gnotobiotic OligoMM^12^ microbiota mouse model and compared the infections in the same facility. We provide evidence of robust colonization and significant pathological changes in OligoMM^12^ mice following infection with these pathogens. Moreover, we offer insights into pathogen-specific host responses and metabolite signatures, highlighting the advantages of a standardized mouse model for direct comparisons of factors influencing the pathogenesis of major food-borne pathogens. Notably, we reveal for the first time that *C. jejuni* stably colonizes OligoMM^12^ mice, triggering inflammation. Additionally, our comparative approach successfully identifies pathogen-specific responses, including the detection of genes uniquely associated with *C. jejuni* infection in humans. These findings underscore the potential of the OligoMM^12^ model as a versatile tool for advancing our understanding of food-borne pathogen interactions.

## Introduction

From the early work of Louis Pasteur and Robert Koch on bacterial infections such as anthrax, animal models have played an important role in understanding infectious diseases and remain important today as in many cases the complexity of host-pathogen interactions cannot (yet) be sufficiently simulated by alternative models.^1–3^ This is especially true for bacterial enteric pathogens as they do not only interact with the host but also with the microbiome residing in the intestine of the host.^4^

Today, infectious disease research is typically conducted by individual research groups focusing on a single pathogen. Every research group establishes specialized models in order to understand the pathogenesis of the pathogen of interest. This approach certainly has advantages, but variations between *in vivo* models and animal facilities typically hinder meaningful comparisons of results.

OligoMM^12^ mice have a defined gut microbiome comprising 12 members that are sequenced and culturable.^5,6^ This community is stable over time and passed on to the offspring.^5^ These properties make it a perfect model to compare entero-pathogens, as the gut microbiome has a major impact on colonization and disease severity.^4,7^ Depending on specific environmental conditions in the respective facilities, mice (C57BL/6J, 129S1) with a complex microbiome display colonization resistance against human entero-pathogens and exhibit a limited disease phenotype.^4^ These mice are often called “specific pathogen free” (SPF) as their microbiome is only defined by the absence of certain potential pathogens and thus varies between animal facilities.^8–10^

*Campylobacter jejuni (C. jejuni)*, *Citrobacter rodentium* (*C. rodentium*, as a model system for EPEC and EHEC), *Listeria monocytogenes (L. monocytogenes)* and *Salmonella enterica* serovar Typhimurium (*S*. Tm) are the major causes of foodborne diseases in Europe and world-wide.^12,13^ Campylobacteriosis, Salmonellosis, Listeriosis and infections with Shiga-toxin producing *E. coli* make up more than 95% of all reported human zoonoses in the EU.^13^

*C. jejuni* is a Gram-negative, spirally curved, motile bacterium with a single flagellum at one or both poles. After ingestion of contaminated food *C. jejuni* triggers enteritis which typically involves fever, vomiting and abdominal cramps in humans.^14,15^ Some individuals suffer from post-infectious immune disorders such as reactive arthritis and Guillain-Barré syndrome.^16^ The established mouse models that are commonly used to investigate the inflammatory potential of *C. jejuni* and the clinical outcome of campylobacteriosis, are wildtype and IL-10-deficient C57BL/6J mice, respectively (in some cases with manipulated microbiome).^4,17^ However, to date, we are lacking a mouse model with a fully defined intestinal microbiota composition for studying colonization resistance against *C. jejuni* and how this pathogen elicits enteric disease.

*C. rodentium* is a Gram-negative, rod-shaped, non-motile murine pathogen, which is broadly used to model infections with enteropathogenic *Escherichia coli* (EPEC) and enterohaemorrhagic *E. coli* (EHEC). *C. rodentium*, EPEC and EHEC share a similar infection strategy and virulence genes.^18^ EPEC mainly causes diarrhea in young children in low- and middle-income countries whereas EHEC is found in industrial countries and can lead to hemorrhagic colitis and hemolytic uremic syndrome (HUS). Utilizing *C. rodentium* as a model pathogen in mice has the advantage that no manipulation of the microbiome is necessary to infect wild-type C57BL/6J (or other strains) mice, even if the mice feature a complex SPF microbiota. In immune competent C57BL/6J mice *C. rodentium* triggers self-limiting colitis and crypt elongation in the distal colon. Clearance of infection usually occurs around 21 days post-infection with a peak of infection around day 8 in immune competent mice.^19^

*L. monocytogenes* is a Gram-positive, rod-shaped, motile bacterium that causes an invasive disease with fever and flu-like symptoms in humans after ingesting contaminated food. Immunocompromised individuals including pregnant women (where infection can lead to spontaneous miscarriage) are most at risk and mortality rate is very high (between 20-30% in specific common-source outbreaks).^20–23^ To efficiently infect mice with *L. monocytogenes* it is necessary to manipulate either the receptor on the pathogen’s surface (InlA) or the receptor (E-cadherin) on the gut epithelial cells of the host.^24–26^ Various studies have compared *L. monocytogenes* infections in conventionally raised mice and those with altered microbiomes (germ-free, monocolonized, or antibiotic pre-treated).^27–30^

*S*. Tm is a Gram-negative, rod-shaped, motile, non-typhoidal member of the *Salmonella* genus. *S*. Tm is one of the most common members of the *Enterobacteriaceae* family that causes gastroenteritis in humans. Humans are most commonly infected by contaminated food like eggs, poultry meat, pork and dairy products^12^ and usually develop symptoms 7-132 h after ingestion.^31^ Disease symptoms typically include abdominal pain (gastroenteritis), diarrhea, nausea and transient fever.^12^ SPF mice harboring a complex microbiota can only be infected if the microbiome is disrupted (antibiotics or diet) or is immature (germ-free, OligoMM^12^, LCM).^4,32^

C57BL/6J OligoMM^12^ mice have been shown to allow colonization by *C. rodentium* (10^8^ CFU/mouse; DBS100),^33^ *L. monocytogenes* (5×10^9^ CFU/mouse; EGD-e)^34^ and *S*. Tm (5×10^7^ CFU/mouse; SL1344).^5^ However, growth kinetics are unknown in case of *L. monocytogenes*. Colonization and disease phenotype of *C. jejuni* infected OligoMM^12^ mice is yet completely unknown.

The aim of the presented work was to analyze colonization kinetics and pathogenicity of four major human pathogens in the same mouse model (OligoMM^12^ C57BL/6^Bern^) to find common and pathogen specific host responses and microbiome changes.

## Methods

### Animal experiments

All animal experiments were approved by the veterinary office in Zurich and conducted under the license number ZH158/19 according to swiss national and cantonal regulations.

**Figure 1.**
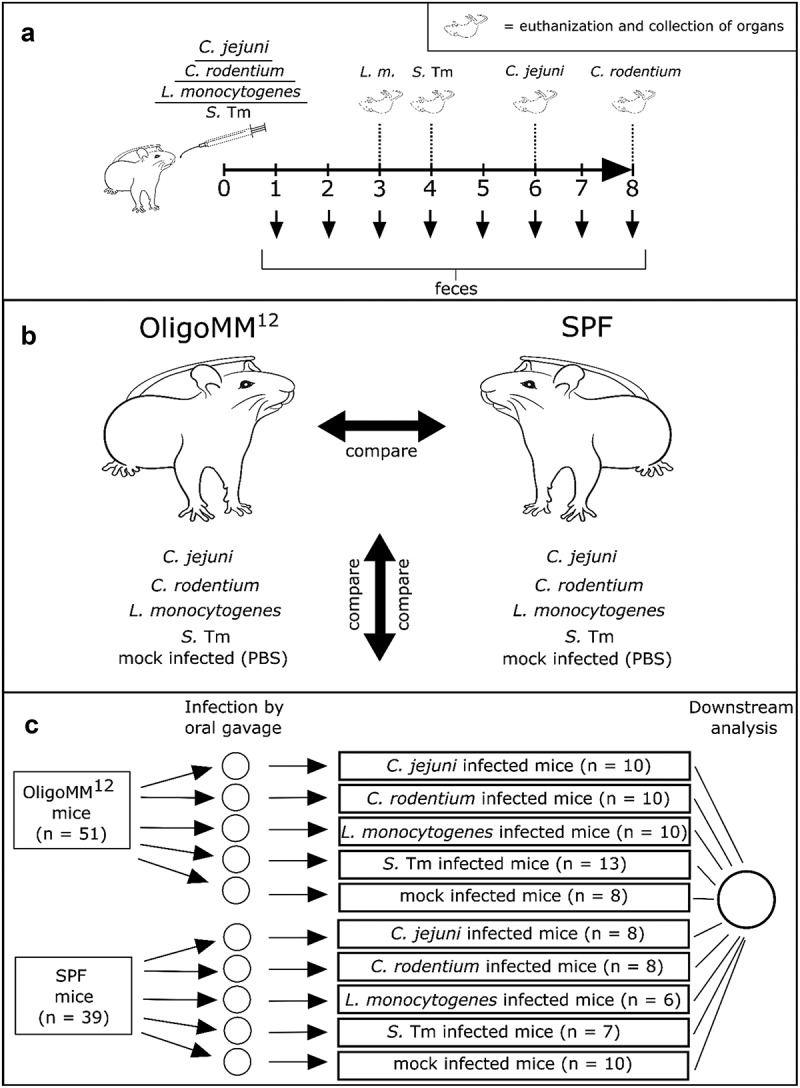
Experimental design. (a) Mice were infected with 10^9 CFU (*C.j.* ; *C.r.* ; *L.m.*), 5x10^7 CFU (*S.* Tm) or mock infected with phosphate buffer saline (PBS; vehicle control). The duration of infection was based on growth kinetics and disease severity for each pathogen (see methods). (b) The experiments were designed to allow comparison in two dimensions, between pathogens and also between OligoMM^12^ and SPF mice.) (c) Number of mice in the different arms of the experiment. Infectious dose of three SPF mice in the *C. rodentium* group had a low infection dose and were thus excluded from the analyses in Figure 2. Mouse illustrations in this figure were purchased from VectorStock.

The OligoMM^12^ B6 line was generated from C57BL/6 germ-free mice that have been inbred within isolator-based colonies for more than 40 generations at the University of Bern, and then at the ETH Zurich (EPIC; 12 h Light/dark cycle, 50% humidity, 25°C temperature). For clarity, we designate this genotype as C57BL/6^Bern^. SPF mice were obtained from The Jackson Laboratory and bred under SPF conditions in individually ventilated cages at EPIC. All mice were transferred to the experimental room at least 1 week before the experiment and kept in IVC cages to acclimatize. (All experiments were conducted with sex-matched male and female mice 8–12 weeks of age at the start of the experiment.) During the experiment, all mice were kept in individually ventilated cages and received sterile mouse chow (3437, KLIBA NAFAG, Kaiseraugst, Switzerland) and water. The mice were gavaged with the indicated dose of pathogenic bacteria (Figure 1) and monitored every day post infection. A fresh feces pellet was collected from each mouse every day after the infection into a sterile and pre-weighted Eppendorf tube. (Feces were homogenized and plated to determine CFU; see below). The endpoint for OligoMM^12^ mice infected with *C. rodentium* or *C. jejuni* was set at 2 days without a rise in fecal CFU densities. For *L. monocytogenes* and *S*. Tm, the endpoint was chosen to minimize animal suffering, as infections lasting for more than 4 or 5 days with wildtype strains of these two pathogens are often lethal. Infection durations in SPF mice matched those of OligoMM^12^ mice for each pathogen. At endpoint, mice were euthanized by CO_2_ asphyxiation, cervical dislocation and immediate removal of vital organs. A macroscopic image of the distal gastrointestinal tract (cecum and colon) was taken with a digital camera (Canon EOS M50 MarkII with Canon EFM 28 mm f3.5 Macro IS STM). Tissue samples of the liver, spleen, proximal ileum, distal ileum, cecum and proximal colon were embedded in cryo-medium (Tissue-Tek® O.C.T.™ Compound) and snap-frozen immediately in liquid nitrogen. Additionally, a tissue sample of the terminal colon was fixed in 4% PFA and paraffin embedded (see “Histology”). A cecum tissue sample was transferred to RNAlater™ (SIGMA) and snap-frozen in liquid nitrogen. Feces and cecum content were transferred to sterile and pre-weighted Eppendorf tubes for CFU quantification (see “Determination of colony forming units”). Liver, spleen and mesenteric lymph node samples were transferred to sterile Eppendorf tubes for CFU quantification (see “Determination of colony forming units”). Two more feces and cecum content samples were transferred into sterile Eppendorf tubes for metabolite analysis (see “Targeted metabolomics”).

### Pathogen strains

We used *Salmonella enterica* serovar Typhimurium (SL1344),^35^ *Listeria monocytogenes* EDG-e (murinized *InlA*),^25^ *Citrobacter rodentium* (ICC169)^36^ and *Campylobacter jejuni* (81–176)^37^ for the *in vivo* infection experiments. *S*. Tm was grown overnight in LB medium. Cells from a 4-hour subculture were washed with PBS and used for inoculation. *C. rodentium* was also grown in LB medium containing 50 µg/mL of nalidixic acid, then washed with PBS before being used for inoculation. *L. monocytogenes* was cultured overnight in liquid BHI medium (brain heart infusion broth; Thermo Fisher Scientific), washed in PBS, and used for inoculation. *C. jejuni* was grown on Karmali plates (Karmali Selective Agar, Blood-Free; Thermo Scientific™) under microaerophilic conditions for 48 hours. A plate with optimal growth was selected, and the cells were scraped and suspended in 1.4 mL of PBS. The inoculum size was verified by plating on the following media: Karmali agar for *C. jejuni*, MacConkey agar (Thermo Fisher Scientific) for *S*. Tm and *C. rodentium*, and selective Listeria agar and BHI agar for *L. monocytogenes*.

### Determination of colony forming units (CFU)

Feces, cecum content and organs (liver, spleen, mesenteric lymph nodes) were homogenized (5 mm steel bead beating) in PBS, diluted and plated on appropriate solid growth medium. (MacConkey for *S*. Tm and *C. rodentium*, Listeria-selective agar (Oxford formulation) for *L. monocytogenes* and Karmali agar for *C. jejuni* and incubated at 37°C. *S*. Tm, *C. rodentium* and *L. monocytogenes* CFU were counted on the next day and *C. jejuni* colonies were counted two days after plating. Plates for analysis of live *C. jejuni* densities were incubated under microaerophilic conditions (CampyGen™ 3.5 L). For feces and cecum content CFUs were calculated per gram of sample (tubes were weighted before and after sample collection) and for the organs CFUs were calculated per organ.

### 16S sequencing

DNA extraction, library preparation and sequencing were performed by SeqBiome (Cork, Ireland). De-multiplexed, paired FASTQ files were returned from the sequencing facility (SeqBiome, Cork, Ireland). The 16S rRNA gene sequencing analysis aimed to elucidate the microbial communities present, focusing initially on rigorous quality control of raw sequence data using FastQC, and the removal of adapters and primers via Cutadapt to ensure sequence integrity. The sequencing targeted the V3-V4 region with specific primers (Forward Primer: 5’ ACACTCTTTCCCTACACGACGCTCTTCCGATCTCCTACGGGNGGCWGCAG; Reverse Primer: 5’ GTGACTGGAGTTCAGACGTGTGCTCTTCCGATCTGACTACHVGGGTATCTAATCC). The cleaned sequences were further processed through the DADA2 pipeline within the QIIME2 framework. Following the examination of a selection of sequence reads with DADA2 to determine quality benchmarks, the forward and reverse reads were shortened to 280 base pairs and 260 base pairs, correspondingly, and requiring a minimum of 20 base pairs overlap for merging reads, thus enhancing sequence assembly accuracy. Following processing, accurate Amplicon Sequence Variants (ASVs) were derived through quality trimming, filtering, error correction, and chimera removal. A custom-trained classifier, developed in QIIME2 based on the Greengenes2 2022.10 reference database for the V3-V4 region, was employed for precise taxonomic classification.

### RNA sequencing

Cecum tissue samples (frozen in RNAlater) were thawed on ice, transferred to RLT buffer (RNeasy Kit, QIAGEN) and homogenized by bead beating (5 mm steel) in a tissue lyzer (QIAGEN) for 3 min at 25 Hz. After centrifugation the supernatant was mixed with 70% EtOH. The rest of the RNA extraction was performed according to the manufacturers protocol (RNeasy Kit, QIAGEN).

The RNA concentration was measured by NanoDrop 2000c (ThermoFisher). The RNA samples were sent to Alithea Genomics (Lausanne, Switzerland) for library preparation, sequencing and data analysis (DE analysis with contrasts: pathogen ~ PBS control and PBS control SPF ~ PBS control OligoMM^12^).

Further data analyses of the differential expression data was performed in R (4.3.2) using the readr,^38^ dplyr,^39^ tidyr,^40^ ggplot2^41^ and VennDiagram^42^ package. Pathway analysis of the generated gene lists (e.g. shared genes of all pathogens, unique genes of single pathogens) were further analyzed using the **D**atabase for **A**nnotation, **V**isualization and **I**ntegrated **D**iscovery (DAVID).^43,44^

### Lipocalin-2 assay

The Lipocalin-2 concentrations were determined from feces samples with the DuoSet® Mouse Lipocalin-2/NGAL Kit (R&D systems®) according to the manufacturer instructions.

### Metabolite profiling

The feces and cecum content samples for metabolomics analysis were processed immediately after dissection of the mice with a protocol for amino acid (AA) measurement and short-chain fatty acid (SCFA) measurement. For the AA preparation, samples were diluted to a concentration of 40 µL/mg with PBS and homogenized in a tissue lyzer (no bead) at 25 Hz (2 min). After centrifugation the supernatant was transferred to a new tube and centrifugated again (4°C). The supernatant of this second centrifugation was transferred to a new tube, snap frozen in liquid nitrogen and stored at −80°C. Sample preparation for LC-MS measurement was performed by adding isotope labeled glutamate following two dilution steps (first with H_2_O, second with HILIC buffer; final dilution: 250 ng feces/mL)

For SCFA analysis we used a previously described method by Liebisch et al.^45^ with some modifications. The sample preparation for SCFA measurement was started by adding 70% isopropanol (in H_2_O) and homogenizing by bead beating (5 mm, steel). After centrifugation an isotope labeled standard mix (500 mM D_3_ acetate, D_5_ 500 mM propionate, 500 mM D_7_ butyrate, 300 mM U-^13^C fumarate, 300 mM D_4_ succinate, and 300 mM, U-^13^C malate) was added and the sample was diluted again with 70% isopropanol. Derivatisation of the SCFA was performed using 3-Nitrophenylhydrazine and *N*-(3-dimethylaminopropyl)-*N*′-ethylcarbodiimide hydrochloride (40°C, 30 min) and quenched with formic acid. After another dilution step with 70% isopropanol the samples were stored at −80°C until LC-MS measurement (final dilution: 250 ng feces/mL).

Amino acids analysis by LC-MS was carried out with a Ultimate 3000 HPLC instrument (Thermo Scientific, Waltham, MA, USA) hyphenated to a QExactive plus mass spectrometer (Thermo Scientific, Waltham, MA, USA) based on a previously described Method.^46^ For LC separation we used an AQUITY BEH NH2 column (particle size 1.7 µm; 100 × 2.1 mm, Waters, Milford, MA, USA) with a buffer consisting of 10 mM ammonium formate (short buffer) in water: acetonitrile (50:50) (solvent A) and the same buffer in acetonitrile : water : methanol (95:5:5) as mobile phases. To shift pH 1.76 ml of formic acid were added to both mobile phases. Sample injection volume was 2 µl. At a flow rate of 500 µl min-1 we run a linear gradient with solvent B changing as follows: 0 min: 84.3%; 1.5 min: 84.3%; 5.5 min: 5.3%; 7.5 min: 5.3%; 8 min: 84.3%. Subsequently, the column was equilibrated for 2 minutes at initial condition. Heated electro spray ionization was performed in the positive FTMS mode at a mass resolution of 70’000 (at m/z 200). Source parameters were set as follows: spray voltage: 3.5 kV; S-lens RF level: 50; sheath gas: 50; aux gas: 20; sweep gas: 0; aux gas heater: 350°C.

Short chain fatty acids analysis by reversed phase LC-MS was carried out with an Ultimate 3000 UPLC instrument (Thermo Scientific, Waltham, MA, USA) hyphenated to a QExative plus mass spectrometer (Thermo Scientific, Waltham, MA, USA) as described previously^45^ with slight modifications. For LC separation we used a Kinetex XB C18 column (particle size 1.7 µm; 50 × 2.1 mm, Phenomenex, Torrance, CA, USA) with 0.1% formic acid in water (solvent A) and 0.1% formic acid in acetonitrile as mobile phases. Sample injection volume was 2 µl. At a flow rate of 500 µl min^−1^ we run a linear gradient with solvent B changing as follows: 0 min: 2%; 3 min: 95%; 5 min: 95%; 5.3 min: 2%. Subsequently, the column was equilibrated for 2 minutes at initial condition. Heated electro spray ionization was performed in the negative FTMS mode at a mass resolution of 70’000 (at m/z 200). Source parameters were set as follows: spray voltage: 2.7 kV; S-lens RF level: 50; sheath gas: 50; aux gas: 20; sweep gas: 0; aux gas heater: 350°C.

To quantify the relative amino acid concentrations, peak areas of [M+H]^+^ extracted ion chromatograms (EICs) for the monoisotopic peaks were normalized against the peak area of the labeled internal standard U-^13^C, ^15^N glutamate. In case of derivatized organic acids, peak areas of [M-H]^−^ EICs acetate, propionate, butyrate, malate, fumarate, and succinate were normalized to their labeled internal standard peak areas. Peak areas from all other organic acids were normalized to peak area of labeled succinate.

The statistical analysis was performed using R (version 4.3.1). R packages ggplot2 (version 3.5.0) and ggpubr (version 0.6.0) were used to make boxplots and heatmaps. Wilcoxon test was used to calculate the statistical significance with PBS as the reference group.

### Histology

The cryo-embedded tissue (liver, spleen, cecum, proximal ileum, distal ileum, and proximal colon) was cut to sections of 5 micrometer thickness with a cryotome (CryoStar N×50von expredia) and stained (H&E). Tissue sections were scored (blindly) using a Axioskop 2 plus (Zeiss) microscope with high-power-field (400X) magnification. Scoring was based on four criteria: Submucosal edema (scored 0 to 3), where 0 indicates no edema. Epithelial disruption, with 0 indicating a healthy, intact epithelial layer with no shedding. Goblet cell count (scored 0 to 3), where 0 represents more than 28 goblet cells and 3 represents 1 or no goblet cells. Polymorphonuclear (PMN) infiltration into the lamina propria (scored 0 to 4), where 0 represents 5 or fewer PMNs and 4 represents more than 100 PMNs.

The distal 0.5 cm of the colon was fixed in 4% paraformaldehyde (PFA) for 2 h and then submerged in 70% ethanol until embedding in paraffin. Fixed tissues were paraffin embedded and sectioned at 5 μm. Sections were then stained with hematoxylin and eosin (H&E). When possible, at least 20 well-oriented crypts were measured in a section from each mouse. The mean of each mouse was plotted for statistical analysis. Images were acquired using a Zeiss AxioVision Z1 microscope, using an Axiocam 105 color camera, and processed using Zen 3.5 Blue Version (Carl Zeiss MicroImaging GmbH, Germany).

For immunofluorescence, sections were dewaxed by submersion in Histo-Clear solution twice for 10 min, 100% ethanol twice for 10 min, 95% ethanol twice for 10 min, 80% ethanol twice for 3 min, and PBS-0.1% Tween 20–0.1% saponin (PBS-TS) twice for 3 min. Subsequently, sections were heated for 30 min in demasking solution (0.3% trisodium citrate-0.05% Tween 20 in distilled H_2_O). Once cooled, slides were rinsed in PBS-TS and then blocked in PBS-TS supplemented with 10% normal donkey serum (NDS) for 20 min in a humid chamber, before being incubated with primary antibody diluted in PBS-TS with 10% NDS for 1 hour. Sections were incubated with anti-PCNA antibody (1:500) and anti-*C.rodentium* antibody (1:50). Slides were rinsed twice for 10 min each time in PBS-TS, followed by incubation with secondary antibodies (1:100) and DAPI (1:1000). Details about the antibodies and reagents used can be found in Table 1. Washing steps were repeated before slides were mounted with ProLong Gold antifade mountant. Images were acquired using a Zeiss AxioVision Z1 microscope, using a Hamamatsu microscope camera, and processed using Zen 3.5 Blue Version (Carl Zeiss MicroImaging GmbH, Germany).

**Table 1.** Antibodies and reagents for immunofluorescence staining

Reageant	Source	Identifier
**Antibodies/Fluorescent stains**		
Alexa Fluor® 488 AffiniPure™ Donkey Anti-Mouse IgG (H+L)	Jackson ImmunoResearch	Cat# 715-545-150
Anti-PCNA antibody [PC10]	Abcam	Cat# ab29
Cy™5 AffiniPure™ Donkey Anti-Rabbit IgG (H+L)	Jackson ImmunoResearch	Cat# 711-175-152
DAPI (4’,6-Diamidino-2-Phenylindole, Dilactate)	Invitrogen, ThermoFisher Scientific	Cat# D3571
Rabbit polyclonal anti-C. rodentium O152	Claire Jenkins, Public Health England^11^	N/A
**Chemicals**		
Dulbecco’s Phosphate Buffered Saline (no Mg, Ca)	Sigma-Aldrich	Cat# D8537
Histoclear II	VWR	Cat #360021 h
Normal Donkey Serum	Sigma-Aldrich	Cat# D9663-10 ML
Polysorbate 20, Fisher BioReagents™	ThermoFisher Scientific	Cat# 10485733
ProLong Gold antifade mountant	Thermo Fisher Scientific	Cat# P36930
Saponin Quillaja sp.	Sigma-Aldrich	Cat# S4521-25 G
Tri-sodium citrate (dihydrate)	Sigma-Aldrich	Cat# S4641

## Results

### All four pathogens colonize the gut of OligoMM^12^ mice

While previous work had explored *S*. Typhimurium, *C. rodentium* and *L. monocytogenes* colonization of OligoMM^12^ mice, it had remained unclear if *C. jejuni* would colonize and if this might elicit enteropathy. The latter would be particularly interesting, since we are still lacking robust gnotobiotic models to study this common enteropathogen. To systematically compare gut colonization and disease kinetics of four important enteric pathogens, we chose C57BL/6 mice, as this is the most widely used mouse strain in research.^47^ The OligoMM^12^ model is particularly relevant, as it is a low complexity model that is easily standardized between laboratories and animal facilities in order to limit the confounding microbiome variation that exists between research facilities.^8–10,48^ We chose C57BL/6J mice with a normal *E. coli* and pathogen-free (SPF) gut microbiota to compare OligoMM^12^ mice (C57BL/6^Bern^) to a classical infection model in the field.

We infected OligoMM^12^ and SPF mice via the orogastric route as illustrated in Figure 1 and allowed the infection to progress until a defined endpoint. The endpoint for OligoMM^12^ mice infected with *C. rodentium* or *C. jejuni* was defined as 2 days without rise of fecal CFU densities. The endpoint for *L. monocytogenes* and *S*. Tm experiments was chosen to reduce animal suffering and death by disease (infections longer than day 4 and day 5 in *L. monocytogenes* and *S*. Tm infections are often lethal). The duration of the infection experiments with SPF mice was the same as in OligoMM^12^ mice in the respective pathogen group (see Methods).

**Figure 2.**
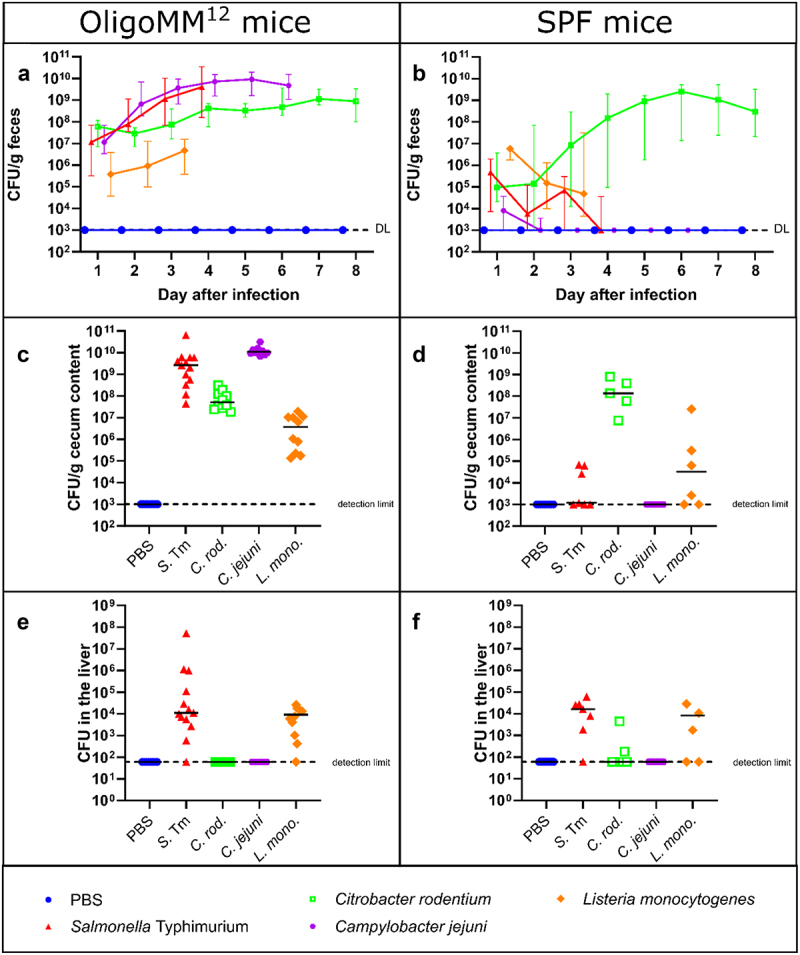
Colonization of OligoMM^12^ and SPF mice with the four pathogens. (a) Median (with range) of CFU per gram in feces of OligoMM^12^ mice for the days post infection (gavage on day 0). (b) Median (with range) of CFU per gram in feces of SPF mice for the days post infection. (c) CFU per gram in the cecum content of OligoMM^12^on the last day of the experiment. (d) CFU per gram in the cecum content of SPF mice on the last day of the experiment. (e) CFU in the liver of OligoMM^12^ mice on the last day of the experiment. (f) CFU in the liver of SPF mice on the last day of the experiment. Solid lines indicate median and dashed lines indicate the detection limit. The data was collected from at least two independent experiments per pathogen group and mouse model. (n of OligoMM^12^ mice: PBS (8), *S*. Tm (13), *C. rodentium* (10), *C. jejuni* (10), *L. monocytogenes* (10); n of SPF mice: PBS (10), *S*. Tm (7), *C. rodentium* (5), *C. jejuni* (8), *L. monocytogenes* (6)).

Our experiments revealed that all four pathogens stably colonized the gut of OligoMM^12^ mice, with an increase in fecal shedding over time, indicating that they are able to grow in the OligoMM^12^ mouse gut (Figure 2a). In contrast, only *C. rodentium* fecal shedding increased over time in the SPF mice (Figure 2b). In SPF mice, *C. rodentium* initially colonizes the cecal lymphoid patch, where it adapts to the gut environment and starts expressing virulence genes (establishment phase, 1–3 days post-infection).^49^ Then, *C. rodentium* sporadically starts to seed the distal colon, which marks the beginning of the expansion phase (4–8 days post-infection), characterized by rapid proliferation of the pathogen in the cecal and the colonic mucosa and increased bacterial shedding in feces.^49^ Fecal bacterial shedding in SPF mice infected with *C. rodentium* follow the kinetics that have previously been reported (Figure 2a,b and S1D). Nevertheless, in OligoMM^12^ mice, high levels of bacterial shedding (10^8^ CFU/g) were seen from the beginning of infection, suggesting the establishment phase is bypassed in the OligoMM^12^ mice and that *C. rodentium* is able to colonize very early on. This is likely due to the absence of bacterial competition and the increase in niche accessibility for *C. rodentium* in the OligoMM^12^ model.

*C. jejuni* pathogen densities reached up to 10^10^ CFU/g in OligoMM^12^ mice (consistent with SAB mice^50^ while the pathogen was undetectable in the feces of most SPF mice after two days (Figure 2a-b and S1B). Fecal shedding of *L. monocytogenes* increased from day 1 to day 3 p.i. in the OligoMM^12^ mice, while they declined in the SPF animals (Figure 2a,b and S1E-F). In line with previous work,^32^ fecal shedding for *S*. Tm increased by about 5–10 fold per day in the OligoMM^12^mice, reaching 10^9^-10^10^ CFU/g by day 4, but remained low (≤10^6^ CFU/g) in SPF mice and declined below the detection limit in approximately half of the mice at days 2–4 p.i. (Figure S1G-H).

The pathogen densities found in the cecum content of the infected OligoMM^12^ mice were significantly higher than in the feces on day 6 of the infection in the *C. jejuni* group and significantly lower than in the feces in *C. rodentium* infected mice at day 8 (Figure S3A). In contrast, fecal and cecal pathogen burdens did not show significant differences in the *S*. Tm and *L. monocytogenes* groups. This suggests that CFU/g in feces is a good approximation of the pathogen amount in the cecum for *S*. Tm and *L. monocytogenes* but to a lesser extent for the other two pathogens. Higher *C. rodentium* CFU in the feces might be explained by the potential bypassing of the establishment phase with the initial colonization of the cecum and the distal colon being the main site of *C. rodentium* growth in the guts of OligoMM^12^ mice (Figure S3A). In contrast, *C. rodentium* yielded approximately equivalent pathogen densities in the cecum content and the feces of SPF mice. We did not observe visual differences in attachment efficiency to the epithelial cells in the colon of *C. rodentium* infected OligoMM^12^ and SPF mice (Figure S4). Overall, these data suggest that the gut microbiota can affect the main site of gut-luminal growth of enteropathogenic bacteria and that the extent of this microbiome-mediated control may differ between the different pathogens (Figure S3B).

We detected viable pathogens in the mesenteric lymph nodes in OligoMM^12^ mice infected with *L. monocytogenes* and *S*. Tm, and in a few of the mice infected with *C. rodentium* and *C. jejuni* (Figure S2C). The mesenteric lymph nodes were colonized in SPF mice but only by *S*. Tm and *L. monocytogenes* (Figure S2D), which is consistent with the well-documented capacity for systemic dissemination of these pathogens.^26,51,52^ Similarly, *S*. Tm and *L. monocytogenes* were consistently found in systemic organs (liver and spleen) of the majority of mice, while *C. rodentium* only occasionally spread to systemic sites in SPF mice. *C. jejuni* was never detectable in livers or spleens (Figure 2e,f and Figure S2A-B). It is interesting to note that *S*. Tm and *L. monocytogenes* CFU in the spleens and livers were equally high in both mouse models, despite the large differences in fecal shedding. This suggests that these two pathogens initiate systemic spread from sites in the upper gastrointestinal tract, e.g. when the inoculum passes through the small intestine during the first hours after orogastric inoculation.

### *C. jejuni*, like the other three pathogens elicits gut inflammation in OligoMM^12^ mice

Next, we asked if high levels of gut colonization in the OligoMM^12^ mice translate into a detectable host response. We observed macroscopic changes of the cecum in OligoMM^12^ mice infected with each of the four pathogens (Figure S5). Most of the ceca from *C. rodentium* infected mice had an inflamed cecal patch but looked otherwise normal. The ceca of *S*. Tm infected mice showed considerable size reduction, a color change as well as a reduced amount of cecum content. This is consistent with earlier observations and indicative of pronounced mucosal inflammation.^5,51^ *C. jejuni* infected mice had slightly shrunken ceca, white patches and a characteristic gas-filled section in the proximal colon. The ceca in the *L. monocytogenes* group showed extensive mouse to mouse variation but some were also reduced in size with white patches and gas filled sections in the proximal colon.

**Figure 3.**
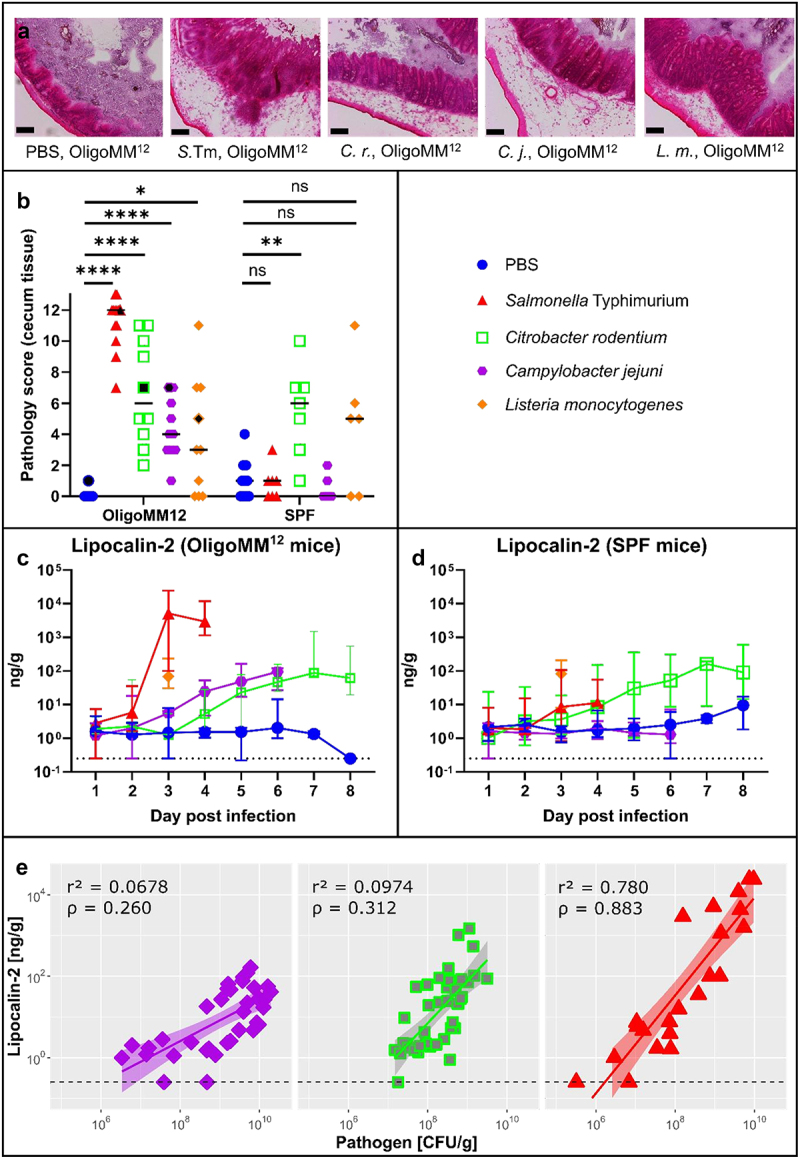
All four pathogens trigger inflammation in OligoMM^12^ mice. (a) Light microscopy images of cecum tissue (H&E staining) of OligoMM^12^ mice. (black bar = 100 µm; corresponding scores indicated with black filled data point in panel B) (b) total pathology score for cecum tissue (H&E staining) of OligoMM^12^ and SPF mice. (mann-whitney test: *p* > .05 = ns, *p* < .05 = *, *p* < .01 = **, *p* < .001 = ***, *p* < .0001) (c) lipocalin-2 levels (as a marker for gut inflammation) in the feces of OligoMM^12^ mice. (no data for *L. monocytogenes* on day 1 and 2) (d) lipocalin-2 levels (as a marker for gut inflammation) in the feces of SPF mice. (no data for *L. monocytogenes* on day 1 and 2) (e) correlation analysis of lipocalin-2 levels vs. pathogen densities in the feces of OligoMM^12^mice. Purple, green and red lines, highlighted with a shaded area, represent the linear model with the corresponding confidence interval. R^2^ value and Pearson correlation were calculated and are shown on the corresponding graph.

Analyzing the tissue from the gastrointestinal tract microscopically, we found significantly elevated levels of enteropathy in the cecum tissue of OligoMM^12^ mice infected with each of the four pathogens (Figure 3a,b). The cecum, proximal ileum, distal ileum, and proximal colon were scored in four categories: We scored the presence and size of submucosal edema, the number of polymorphonuclear neutrophils (PMNs) per optical field, the number of goblet cells with mucus-filled vacuoles and the loss of epithelial integrity, as described before for *S*. Tm^53^ (Materials and Methods). The scores of the four categories were added together to obtain a total pathology score. Importantly, *C. jejuni* caused significant enteropathy in the OligoMM^12^ mice. This indicates that the OligoMM^12^ mice can offer a gnotobiotic model to study this pathogen’s enteric virulence. Interestingly, *C. jejuni* elicited lower degrees of pathology than *S*. Tm infection. Some *C. rodentium* animals and one *L. monocytogenes* infected mouse had similarly high scores as *S*. Tm, while others showed more benign enteropathy in OligoMM^12^ mice, comparable to the *C. jejuni* infections (Figure 3b).

A hallmark of *C. rodentium* infection is an increased length of the crypts in the terminal colon of infected mice.^54^ These phenotypes were seen in both OligoMM^12^ and SPF mice (Figure S2E-F). Additionally, we found significantly elevated crypt lengths in *L. monocytogenes* infected SPF mice but not OligoMM^12^ mice suggesting that colonic mucosa pathology may be dependent on unknown disease-modulating effects of the resident gut microbiota. Interestingly, the *C. jejuni* infected OligoMM^12^ mice featured elevated crypt lengths which shows that the distal colon is also affected by the high *C. jejuni* densities in this mouse model. No crypt elongation was detected in the *S*. Tm group (neither in OligoMM^12^ nor in SPF mice) despite massive cecum tissue damage. This confirms the cecum as the main replication site of *S*. Tm in mice.

The gut inflammation marker lipocalin-2 results were consistent with those obtained from histopathological analysis (Figure 3b–d). Measuring lipocalin-2 concentrations in the feces of infected mice offers the advantage of noninvasive, continuous monitoring throughout the infection experiment without the need of euthanization.

We observed a strong difference between the pathology scores and lipocalin-2 levels in OligoMM^12^ compared to SPF mice infected with *S*. Tm which indicates that the gut microbiota have a pronounced effect not only on gut colonization but also protect the host from severe symptoms. Similar observations were made for *C. jejuni*, despite lower inflammation levels (compared to *S*. Tm) in highly colonized (~10^10^ CFU/g) OligoMM^12^ mice (Figures 3 and 2a). In contrast, *L. monocytogenes* and *C. rodentium* infection elicited similar inflammation levels in OligoMM^12^ and SPF mice. The latter is not surprising since we also observed similar colonization levels (Figure 2a,b) in both mouse models. However, colonization levels of *L. monocytogenes* differed between OligoMM^12^ and SPF mice but did not translate into a difference in inflammation levels.

In *S*. Tm and *C. rodentium* infections, gut inflammation is well established to promote gut-luminal pathogen growth. This has been attributed to inflammation-mediated suppression of microbiota growth (or re-growth after streptomycin pre-treatment).^55–58^ To assess, if this is also true for *C. jejuni*, we plotted the fecal lipocalin-2 concentrations (from Figure 3c) against the fecal pathogen loads (Figure 2a). This analysis yielded a strong positive correlation for the *S*. Tm group and a weaker positive correlation for the *C. rodentium* and *C. jejuni* infected mice (Figure 3e). Unfortunately, we did not obtain enough data to perform a similar analysis for *L. monocytogenes*. Nonetheless, our observations generally show that gut inflammation correlates with gut-luminal pathogen densities. It remains however unclear, if gut inflammation is the cause or the consequence of enhanced gut-luminal growth of *S*. Tm, *C. rodentium* and *C. jejuni*. This will be an interesting question to address in future work.

### Comparative approach allows determination of common and unique host/microbiota responses to pathogen infection

#### Specific T-cell response in *C. jejuni* infected OligoMM^12^ mice at day 6 p.i.

To gain a deeper insight into the differences and similarities of the host immune response to the four pathogens, we analyzed the gene expression patterns of the cecum tissue. The cecum tissue was chosen for differential gene expression analysis because we saw high pathogen burden in the cecum content (Figure 2c) of infected OligoMM^12^ mice. The data suggested that this is a relevant site of pathogen growth and significant pathological changes were observed in the tissue (in OligoMM^12^ mice) (Figure 3a,b). Differential gene expression analysis was performed between the pathogen group and the corresponding mock infected (PBS) group in OligoMM^12^ (Figure S6, Table S1–2) and SPF mice (Figure S7). Additionally, we compared the gene expression between mock-infected OligoMM^12^ and SPF mice to understand the baseline differences between the two mouse models (Figure S8). Overall, we detected mRNAs for around 11,000 to 13,000 genes in every group (OligoMM^12^/SPF) with the highest proportion of differentially expressed genes (27%) in the *S*. Tm group (OligoMM^12^ and the lowest proportion of differently expressed genes (1%) in the *C. jejuni* group (SPF mice; Figure S9).

Principal component analysis of the expression data showed good separation of the mock treated (PBS) OligoMM^12^ mice and the pathogen infected in all four groups (Figure S10). On the other hand, mock (PBS) infected OligoMM^12^ and mock infected SPF mice cluster together (Figure S8). This shows not only that all four pathogens trigger a detectable host response in OligoMM^12^ mice but also that these mice have a similar baseline gene expression in the cecum tissue compared to SPF mice. The latter stresses the usefulness of the defined microbiota model (OligoMM^12^) as a proxy for SPF mice to analyze host-pathogen-microbiota interactions.

**Figure 4.**
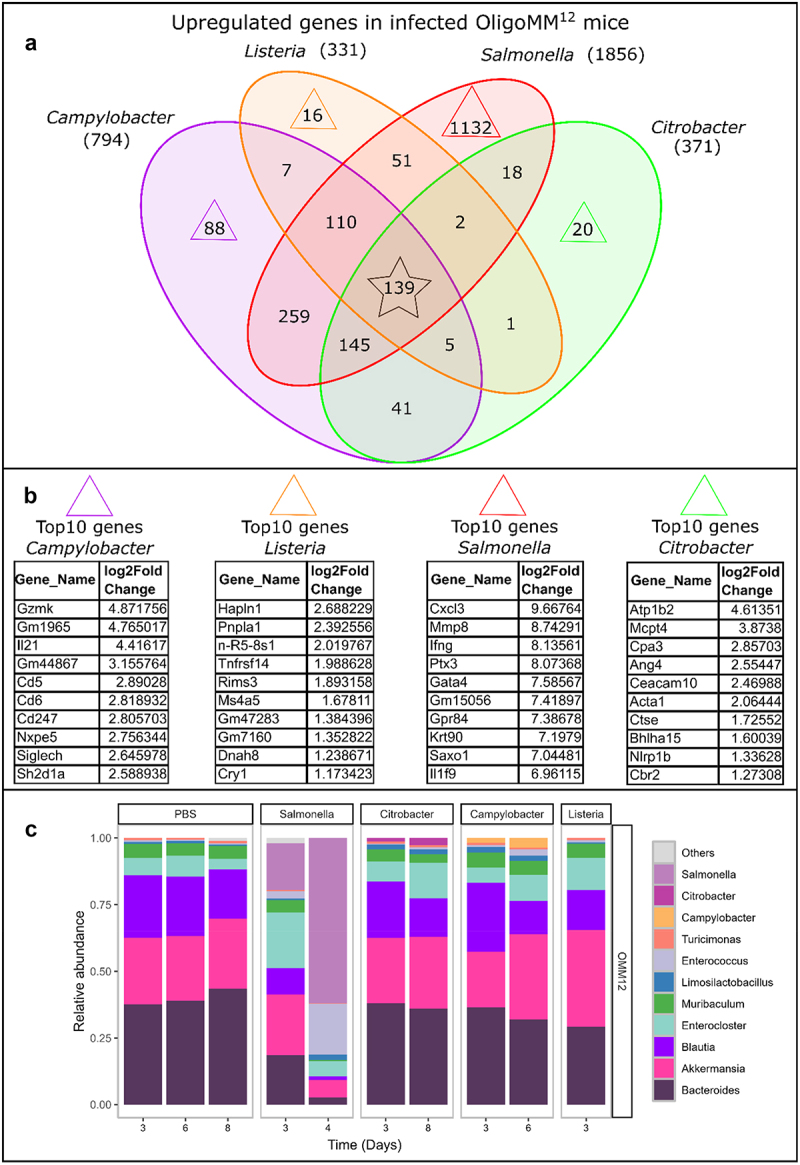
Host and microbiota response to pathogen infection in OligoMM^12^ mice. (a) Number of significantly upregulated genes unique to and shared between pathogen groups. Every colored circle contains the total number of significantly upregulated genes in that pathogen group. The number of shared genes between the respective groups is shown wherever circles overlap. Triangles mark the number of genes that are unique to each pathogen group. The black star marks the number of genes that are significantly upregulated in all pathogen groups. (b) Top10 upregulated genes that are unique to the pathogen group. (c) Relative abundance of bacterial taxa in the feces of infected/mock treated (PBS) OligoMM^12^ mice on day 3 and the last day of the experiment for the individual pathogen groups.

To better understand the cecum tissue responses that are common between some pathogens or unique between others we compared the differentially expressed gene lists to find overlaps (Figure 4a). Additionally, to gain deeper insights into the function and pathways associated with the genes of these created groups, we performed DAVID enrichment analyses (see Methods). As a common general element in the mucosal immune response to the infection by all four pathogens we found upregulated genes (139, Figure 4a) involved in innate immunity, cellular response to interferon gamma (INFγ), interferon beta (INFβ), lipo-polysaccharide (LPS) and activity of GTPase proteins (Figure S11).

Our data analyses revealed that the activation of adaptive immune responses in the cecum tissue, more specifically Th1, Th2 and Th17 mediated responses, is unique to *C. jejuni* infection in OligoMM^12^ mice (Figure S12). In line with that we found IL-21, granzyme K and the cell surface antigens CD247, CD5 and CD6 to be among the top 10 upregulated genes (unique to *C. jejuni* infection; Figure 4b). This expression profile suggests the involvement of specific T-cell components, particularly CD247 as part of the CD3 subunits of the TCR complex. Future work should confirm the hits and dissect this in more detail.

Also, the upregulation of CD5 and CD6 receptors offers new insights into their roles in the context of *C. jejuni* infections. Given *C. jejuni*‘s known activation of cross-reactive T cells in humans,^59^ these findings emphasize the value of OligoMM^12^ mice as a model for *C. jejuni* infections. This model could be particularly useful for dissecting the immune dynamics of *C. jejuni* infection, particularly the Th1 single, and Th17/Th1 dual immune responses, closely mirroring these observed in human infections and the extensively studied in IL-10^−/−^ mice.^59,60^ Importantly, these findings highlight the key role of T-cells in eliciting the cell-mediated response to *C. jejuni* infection. On the other hand, these responses were not detected in resistant *Campylobacter*-infected SPF mice (presumably due to colonization resistance), further highlighting the value of OligoMM^12^ mice as a model for *C. jejuni* infections. However, we want to emphasize that we did not confirm the expression levels of the above mentioned genes by qPCR, which should be done before drawing any further conclusions.

#### *C. jejuni, C. rodentium* and *L. monocytogenes* infections affect the OligoMM^12^ microbiota much less than *S*. Tm

To understand the impact of infection on the microbiome of OligoMM^12^ we used 16S rRNA sequencing (Figure 4c, S16–21) and analyzed the feces from day 3 (every pathogen group) and from the last day of the infection (which varies depending on the pathogen group). We did not find major changes in the microbiome structure of infected OligoMM^12^,except for the *S*. Tm group (Figure 4c, S15). In *S*. Tm infected OligoMM^12^ mice we found a massive shift toward facultative aerobic bacteria (*S*. Tm and *Enterococcus*) which is in line with previous findings^61^ in *S*. Tm infected SPF and OligoMM^12^ mice. *S*. Tm takes over more than 50% of the gut population in OligoMM^12^ mice on day 4, which is likely due to the high levels of inflammation and associated rise in the concentrations of terminal electron acceptors like oxygen species, tetrathionate of nitrate in the gut.^62–68^

Our data verifies that the *S*. Tm triggered gut inflammation in OligoMM^12^ mice leads to a strong shift in microbiota composition. In contrast, the other three pathogens appear to colonize the murine gut, while leaving the global microbiota composition intact. Our work does not exclude the possibility of local microbiome changes which are limited to the mucosal surface and could form the basis of further study. However, to gain a broader understanding of how entero-pathogen infection impacts the gut environment as a whole, we shifted our focus to global metabolic changes in the feces (and cecum content).

#### All four pathogens elicit elevated succinate levels in the OligoMM^12^ mouse gut compared to mock infected controls

**Figure 5.**
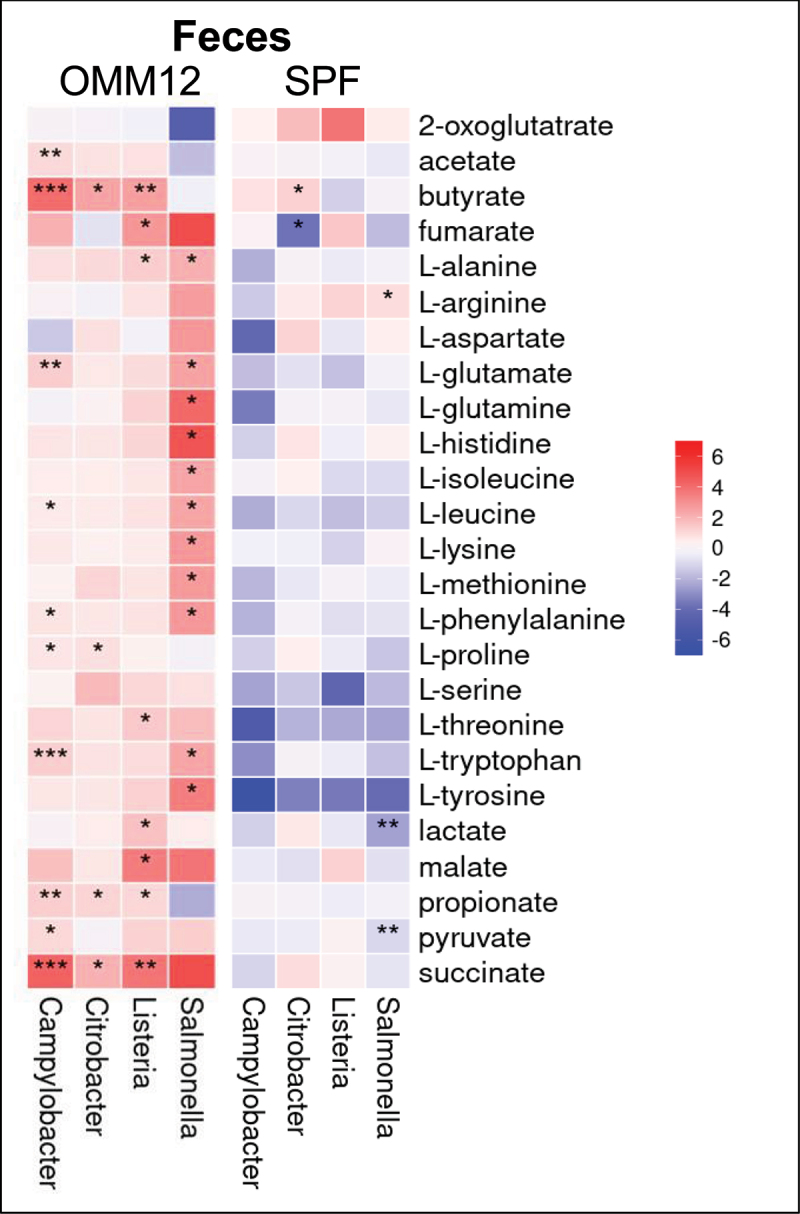
Heatmap of metabolite abundance in the feces of OligoMM^12^ and SPF mice relative to mock (PBS) treated mice. Color gradient indicates log2 fold change of average peak area of metabolites relative to PBS, and the stars indicate significance. (Wilcoxon test: *p* < .05 = *, *p* < .01 = **, *p* < .001 = ***, absence denotes non-significant values).

The human and the mouse gut contain dietary substrates and products of microbial and host metabolism, many of which can mediate microbe-host interactions.^69,70^ As the growth of bacteria and release of metabolic products is intimately linked to the availability of nutrients in the gut environment^71^ we investigated changes in certain metabolites due to entero-pathogen infection. We utilized an existing targeted metabolomics pipeline to measure an array of 25 compounds including amino acids and short-chain fatty acids (SCFAs) in the cecum content and the feces of the mice at the endpoint (varies depending on pathogen, see Figure 1). Amino acids in the host diet may serve as carbon and nitrogen sources for microorganisms.^70^ However, the uptake of amino acids by the host in the small intestine is very efficient, reducing the availability of these nutrients for the majority of the gut microbial community in the more distal parts of the GI tract.^70,72–74^ Short-chain fatty acids (SCFAs) on the other hand, are produced by anaerobic bacteria in the gut (*Bacteroidetes* and *Firmicutes*) by fermentation of dietary-fibers.^75^ These SCFA are absorbed by intestinal epithelial cells and serve important functions in the homeostasis of a healthy gut.^76^ In OligoMM^12^ mice infected with either of the four pathogens we observed an overall increase in most of the metabolites in the feces and cecum content compared to mock (PBS) infected OligoMM^12^ mice (Figure 5, S22). This could be explained by inflammation due to entero-pathogen infection and a resulting reduction in nutrient absorption by the host. We do not know if amino acids released by dying gut epithelium cells may also contribute.

Butyrate and propionate levels were found to be significantly increased in the feces of infected OligoMM^12^ mice in all pathogen groups except for the *S*. Tm group (Figure 5, S22). In the *S*. Tm group, we observed a decrease (even though not always significant) in these two metabolites in the feces (Figure 5) as well as cecum content (Figure S22). This could be due to the significant decline of SCFAs producing *Bacteroidetes* and *Firmicutes* on day 4 in OligoMM^12^ mice infected with *S*. Tm (Figure S25). In contrast, the increase of these metabolites in the other pathogen groups (inflammation levels too low to cause microbiota shift) might reflect the decrease in uptake of SCFA by the intestinal epithelial cells as a result of low-level inflammation and immune system activation. Butyrate has been shown to indirectly inhibit pathogen growth by manipulating host responses e.g. IECs and intestinal macrophages which in turn reduce oxygen availability and activate antimicrobial functions.^77^ Cross comparison of the impacts of the four pathogens in our study suggests that butyrate is available in higher concentrations during low level inflammation but absent after a certain inflammatory threshold (somewhere between 10^2^-10^3^ ng/g lipocalin; Figure 3,5) is exceeded, as is the case in *S*. Tm infection.

In *S*. Tm infected OligoMM^12^ mice we found elevated levels of almost all amino acids measured (Figure 5). This is likely due to either the death of host cells that are expelled into the gut lumen^78^ or the death of microbiota members caused by inflammation, which leads to the release of their contents into the gut. Under physiological conditions the amount of AA found in the large intestine and the cecum is rather low as most of the AAs from food are already absorbed in the upper parts (stomach and small intestine) of the GI tract.^70^

Furthermore, we found increased amounts of succinate in the feces and cecum content of infected OligoMM^12^ mice in all pathogen groups (Figure 5 and S22). *S*. Tm uses malate (and aspartate) to produce the electron acceptor fumarate, which is required in an incomplete (and reverse) TCA cycle for initial growth in the mouse gut.^79^ This metabolic pathway of fumarate respiration results in an overproduction of succinate that is exported from the *S*.Tm cell into the gut lumen.^79^ *C. jejuni* and *L. monocytogenes* are as well capable of fumarate respiration under anaerobic conditions^80,81^ and *C. rodentium* carries the genes for the fumarate reductase and the transporter (*dcuABC)* for succinate export. Additionally, we detected significantly less succinate in mock (PBS) treated OligoMM^12^ mice than in mock (PBS) treated SPF mice (Figure S23-24). These observations may hint that fumarate respiration could be a central metabolic pathway for entero-pathogens to colonize the gut.

## Discussion

The presented data establishes OligoMM^12^ mice as a mouse model for *C. jejuni* infections which allows to study host-pathogen-microbiota interactions. Our side-by-side comparison to three other enteropathogenic bacteria highlights the versatility of the OligoMM^12^ mouse model beyond *S*. Tm infection experiments toward a universal model to investigate growth kinetics and host response against a wide range of enteropathogenic bacteria. The comparison of different enteropathogenic bacteria in this gnotobiotic model provides a platform to dissect the common anti-microbial defense pathways of the murine mucosa and to discover unique infection strategies employed by the different pathogens.

Gnotobiotic mouse models allow the supplementation of additional microbiota members, e.g. for studying their effect on colonization resistance. As *C. jejuni* typically presents an all-or-nothing infection phenotype^50,82^ an interesting approach going forward would be to add microbial strains to the OligoMM^12^ consortium to identify commensal strains capable of reestablishing colonization resistance, as this was achieved for *S*. Tm.^5^ An initial approach would be to select bacteria (e.g. from the mouse intestinal bacterial collection – miBC^83^) that are capable of metabolizing the substrates which are suspected to be important for *C. jejuni* growth in the OligoMM^12^ mouse gut.^84–88^

It seems that triggering strong inflammation is a *S*. Tm specific strategy to disrupt the resident microbiota and thus create niches to grow, while the other three pathogens manage to grow to high numbers while eliciting lower grades of gut inflammation. The advantage of this brute-force strategy of *S*. Tm could be to open up multiple nutrient niches at once which increases the chance to obtain at least one niche that provides enough resources for massive proliferation and transmission to a new host. As a generalist, *S*. Tm probably has the option to colonize a variety of niches in the gut. *C. jejuni* has a quite different metabolic strategy, as indicated by its incapacity to metabolize many common carbohydrates.^88^ *C. jejuni* is however able to metabolize the amino acids aspartate, glutamate serine and proline.^88,89^ Thus, elevated proline levels in OligoMM^12^ mice (compared to SPF; Figure S23–24) might play a role in supporting *C. jejuni* growth, highlighting proline metabolism as a promising target for further investigations.

Furthermore, the vast majority of analyses concerning the involvement of the immune system in *C. jejuni* infection was performed with C57BL/6J IL-10^−/−^ mice.^82,90^ We provide evidence that the OligoMM^12^ model could be a good alternative to a mouse strain with a knockout in an essential immunological pathway. The Th1/Th17 mixed response in the cecum tissue of infected OligoMM^12^ mice seems to be unique to *C. jejuni* infection (Figure 4a,b) in our experiments, which was also shown to be a characteristic of *C. jejuni* infection in human intestinal tissue.^59^ The Th1/Th17 mixed response also characterizes colitis in IL-10^−/−^ mice infected with *C. jejuni*.^60^ We did detect cellular responses to IFN-gamma (Th1 response^91^ as a common feature of gene expression against all pathogens (Figure S11). However, IL-21 (Th17 response^92^) was uniquely upregulated in *C. jejuni* infected OligoMM^12^ mice (Figure 4b, S12). The lack of a similar response in *L. monocytogenes* ^93^ and *S*. Tm^94,95^ infected mice might be explained by the (earlier; day 3/4 vs. day 6 post infection; Figure 2) timing of our measurements for these pathogens.

OligoMM^12^ mice offer several model-specific advantages. Unlike secondary abiotic^96^ or humanized microbiota mice,^82^ OligoMM^12^ mice offer the benefits of a well-defined microbiome, lower variability between mice, and no need for extra interventions (antibiotics) that could have an effect on the host^5^(Figure 1). Compared to germ-free mice, which tend to show extremely fast gut-luminal pathogen growth for *S*. Tm^5,32,61,97,98^ and *C. rodentium* ^99^ (≥10^9^ cfu/g stool within 8-24 h), along with an earlier and more severe disease phenotype, the colonization kinetics and the onset of disease are delayed in OligoMM^12^ mice (≥10^9^ cfu/g stool within >72 h; Figure 2). *C. jejuni* and *L. monocytogenes* growth kinetics appear to be similar in OligoMM^12^ and germ-free mice^28,100^ (Figure 2). It remains to be investigated if pathology and disease outcome is milder in *C. jejuni* or *L. monocytogenes* infected OligoMM^12^ mice compared to germ-free mice. This suggests that the gnotobiotic microbiota of OligoMM^12^ mice confers considerable colonization resistance at least against some of the four pathogens. This colonization resistance slows down pathogen growth and thereby permits studying protective roles of the gut microbiota. It also indicates that their more mature immune system enables protective responses to acute bacterial infection, which are lacking or reduced in germ-free animals.^83^ Based on these observations, it will be an interesting task for future work to compare *C. jejuni* disease dynamics and immune responses in OligoMM^12^ mice reported here, to those in GF mice.

Despite its advantages for *C. jejuni* infections, the OligoMM^12^ model also has some disadvantages. This model only simulates a less severe type of Campylobacteriosis without visible bloody diarrhea, which is the case for most *Campylobacter* infections with the same *C. jejuni* strain in humans.^37^ Lower levels of inflammation and tissue damage also decreases the range of detection and makes it harder to measure the positive effects of interventions. Future work should address if IL-10 knockout mice with an OligoMM^12^ microbiota may provide a means to study these more severe mucosal disease phenotypes elicited by *C. jejuni*. Despite high similarity of the baseline gene expression in the cecum tissue of OligoMM^12^ mice compared to SPF mice (Figure S8), there are physiological differences (most notably cecum size or carbohydrate concentrations^101^ in the mice carrying these defined microbiota).^83^ This is probably due to missing microbial functions in the OligoMM^12^ consortium and advancements have recently been made to improve the model.^83^

The kinetics of disease progression are one critical parameter of an enteric infection. Changes in gut colonization and the onset of gut inflammation can be employed to study the role of virulence factors of a given enteropathogenic bacterium. As a natural mouse pathogen, *C. rodentium* is able to colonize the gut despite the presence of a complex microbiota, and even takes advantage of some commensals for effective colonization.^102^ Interestingly, when infecting OligoMM^12^ mice, *C. rodentium* seems to colonize the gut at high levels from early on. This could indicate that in this model, the establishment phase, where *C. rodentium* infects the cecal lymphoid patch, adapts to the gut environment and expression of virulence genes is induced,^103^ is bypassed, emulating the infection cycle of ‘hyperinfectious’ *C. rodentium* after passage through the host.^104^ These observations highlight the importance of simplified microbiota models to explore the interactions that drive the activation of virulence genes and their necessity in complex microbiota systems.

We show that Lipocalin-2 can be used as a continuous noninvasive readout for the inflammation level in OligoMM^12^ mouse infections with all four pathogens. Lipocalin-2 levels correlate with *C. jejuni*, *C. rodentium* and *S*. Tm densities in the feces of OligoMM^12^ mice (Figure 3e). Apart from the typical crypt length increase in the distal colon due to *C. rodentium* infection our data suggests that the cecum tissue can also serve as a valuable site for analyses on host response for all of the pathogens analyzed.

The strategy of the murinized *L. monocytogenes* strain used in our study appears to be rapid tissue invasion which is independent of the pathogen burden in the cecum. The OligoMM^12^ mouse model allows for *L. monocytogens* growth in the gut, but this had no impact on the pathogen burden in systemic sites such as the liver (Figure 2; S1; S2). We used a murinized *L. monocytogenes* strain to enable our comparative approach, though we appreciate that murinized InlA has the potential to trigger N-cadherin-mediated (not just E-cadherin) responses in the host which is artificial and cannot be observed in humans or humanized mouse models.^26^ It is known that *L. monocytogenes* replicates in the murine gall bladder and is released into the gut most likely to aid onward transmission to another host.^105^ Thus, this pathogen might be less affected by the microbiome composition in the cecum and colon with dynamics of gastrointestinal colonization being influenced by systemic infection dynamics and growth in the gall bladder.

We show that succinate accumulates in feces of OligoMM^12^ mice colonized by any of the four pathogens (Figure 5). Succinate is an end product of fumarate respiration and this pathway has already been shown to be an important factor for *S*. Tm infections.^79,106^ In-depth investigations of fumarate respiration of all four pathogens in the OligoMM^12^ mouse model seems to be a promising approach to find a universal target to interfere with an important metabolic pathway. The presented work shows that OligoMM^12^ C57BL/6^Bern^ mice provide a valuable system to investigate a variety of enteric pathogens and offer the opportunity for standardization in the research field of food-born infections.

## Supplementary Material

Supplemental Material

## Data Availability

The data supporting the findings of this study are available as follows:
16S rRNA Gene Sequencing Data: The raw sequence data has been deposited in the NCBI Sequence Read Archive (SRA) repository under the BioProject ID PRJNA1152786. RNA-seq Data: The RNA sequencing data are available in the ETH research collection under the accession number 10.3929/ethz-b-000693607. Targeted Metabolomics Data: The metabolomics data, including both raw data and processed metabolite quantifications, are available in the MetaboLights repository^107^ under the accession number MTBLS11078 (www.ebi.ac.uk/metabolights/MTBLS11078.) Bacterial Densities (CFU/g): The colony-forming unit (CFU) data per gram of sample are included in the supplementary materials of this publication and can be accessed directly. Image Data (H&E, IF Staining and Macroscopic Images): The macroscopic images of cecum and colon are available in the ETH research collection under the accession number 10.3929/ethz-b-000707980. The images of immunofluorescence and H&E staining of the colon are available on Zenodo with the accession IDs 10.5281/zenodo.14276427 and 10.5281/zenodo.14392311 Pathology Scores: The detailed pathology scores used in this study are provided in the supplementary materials. Lipocalin-2 ELISA Data: The ELISA data for lipocalin-2 quantification is included in the supplementary materials. For additional information or specific data requests, please contact the corresponding author.

## Code availability

The code used to process and analyze the 16S rRNA sequencing data is available in the GitHub repository at https://github.com/LudinusDaleth/Pathogens-in-OMM12/tree/main.

## Highlights

- *Campylobacter jejuni* stably colonizes OligoMM^12^ mice and triggers inflammation in the cecum
- Only *S*. Tm triggers inflammation that is strong enough to disrupt the microbiome
- Validation of the comparative approach by identifying genes known to be important in human *C. jejuni* infection
