# Supplementary material for: Comparing Campylobacter jejuni to three other enteric pathogens in OligoMM12 mice reveals pathogen-specific host and microbiota responses
Source: Gut Microbes. 2025 Jan 21;17(1):2447832. doi: 10.1080/19490976.2024.2447832 (PMC12931697; doi:10.1080/19490976.2024.2447832)
Supplement: Supplemental Material [file KGMI_A_2447832_SM7659.zip › 2447832/20250112_four_path_supplements_revised.docx]

Comparing *Campylobacter jejuni* to three other enteric pathogens in OligoMM^12^ mice reveals pathogen specific host and microbiota responses

Supplements


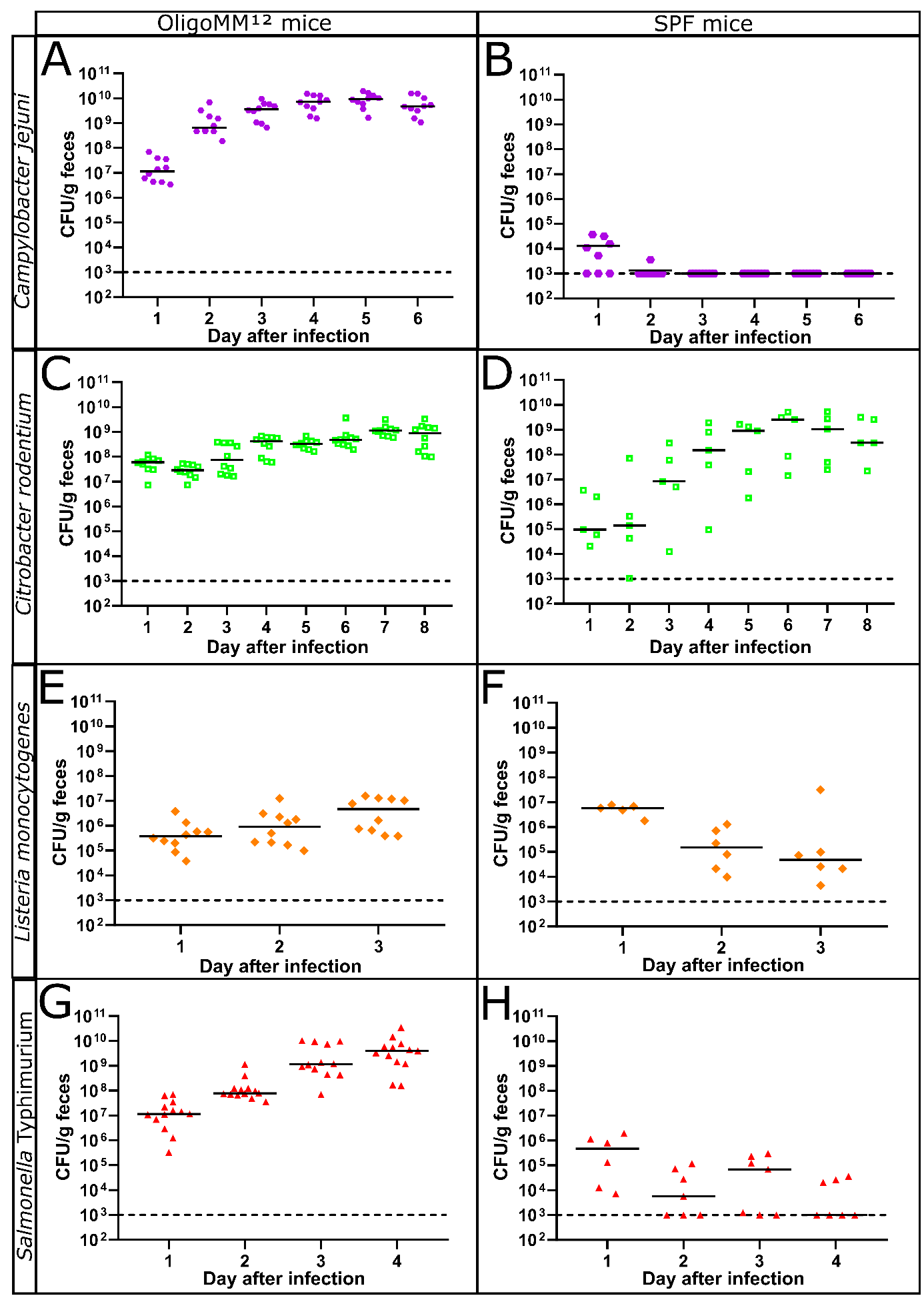


Figure 1: Fecal densities of the four pathogens in OligoMM^12^ and SPF mice.


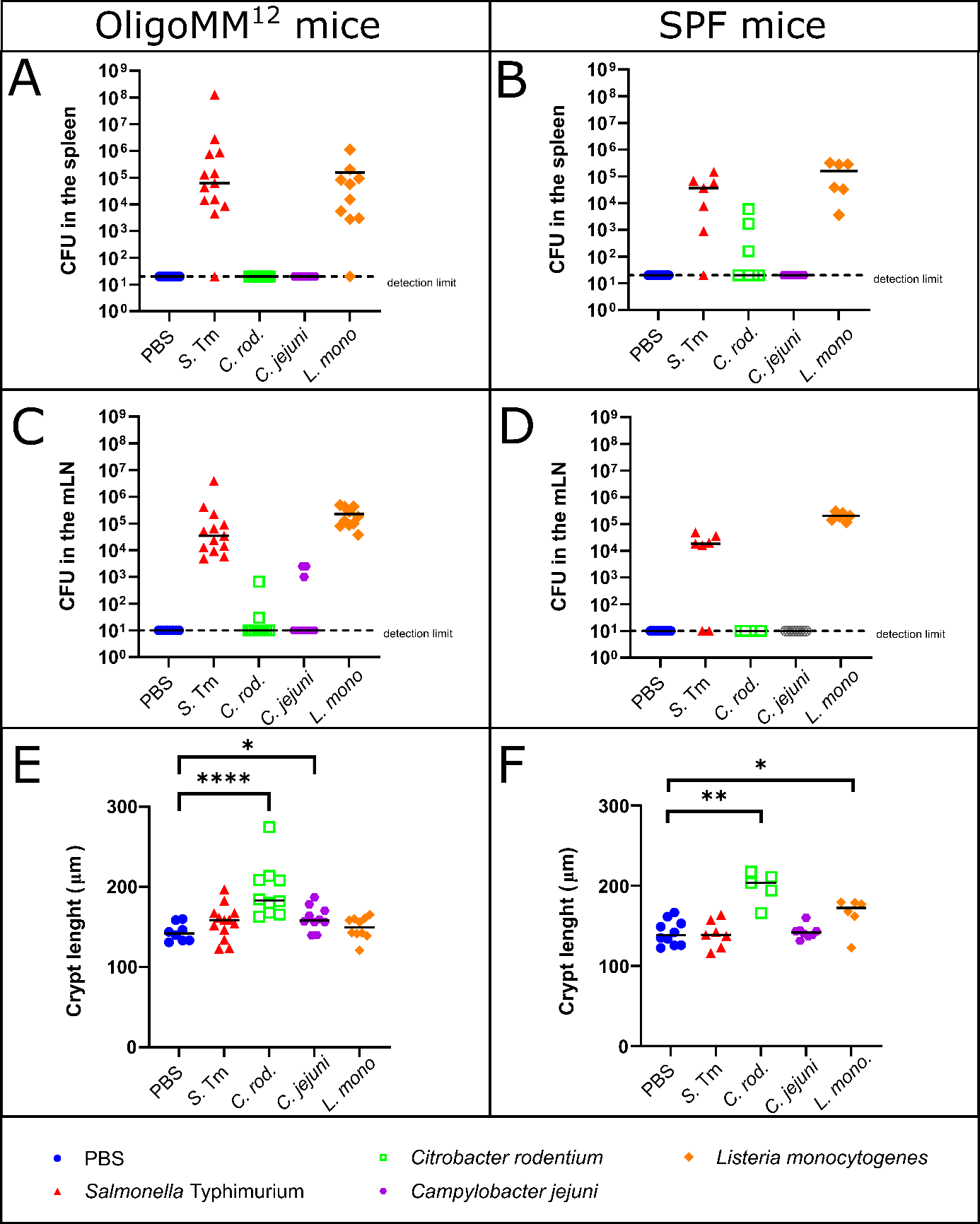


Figure 2: Colony forming units (CFU) in spleen and mesenteric lymph nodes (mLN) of OligoMM^12^ and SPF mice as well as crypt length in the distal colon.


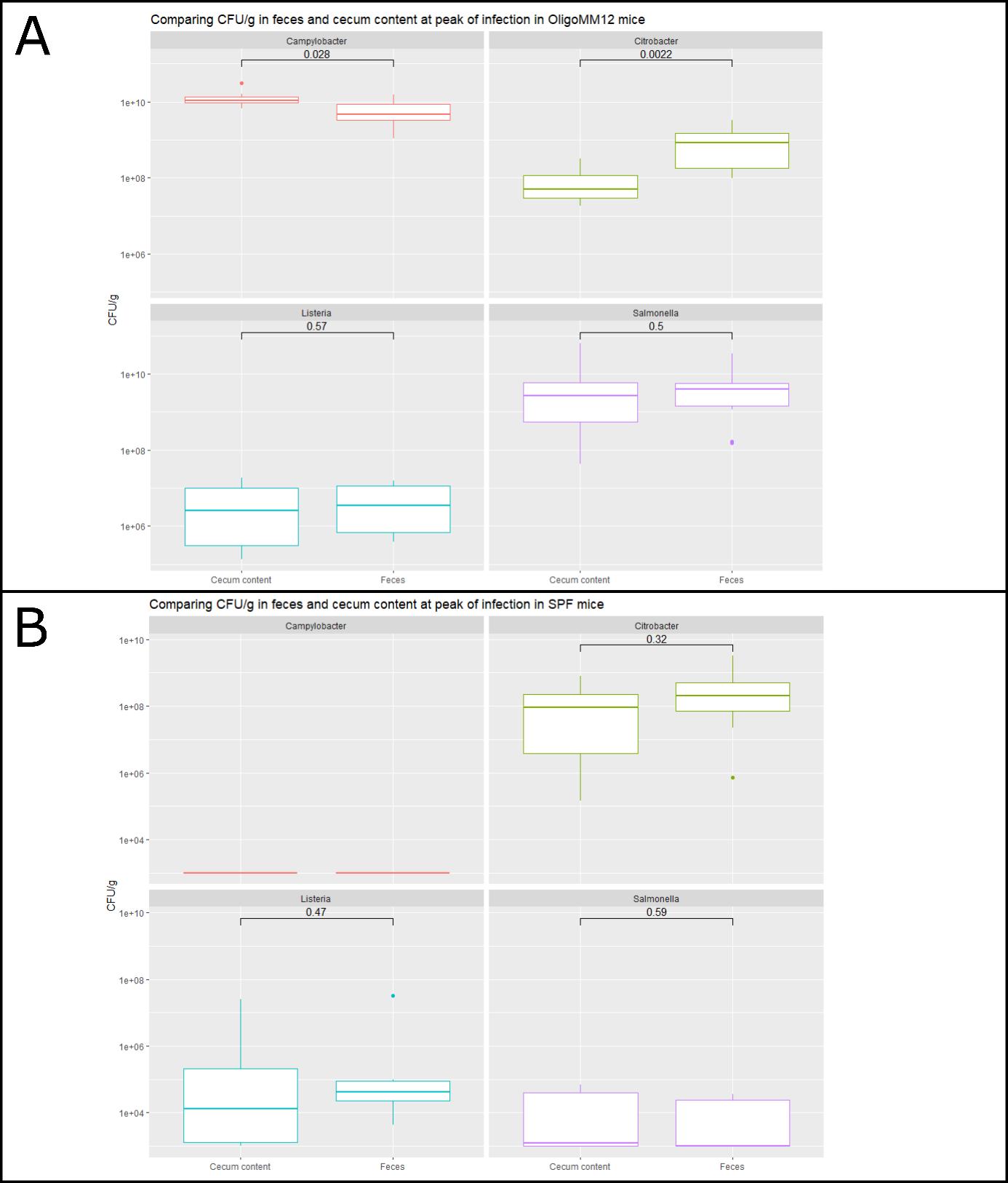


Figure 3: Comparing CFU/g of feces and CFU/g of cecum content on the last day of the experiment for all four pathogen groups in the two mouse models. A) CFU/g in feces or cecum content on the last day of infection in OligoMM^12^ mice. B) CFU/g in feces or cecum content on the last day of infection in SPF mice. Detection limit: 1000 CFU/g. Statistics: Wilcox test with approximate p-value calculation.


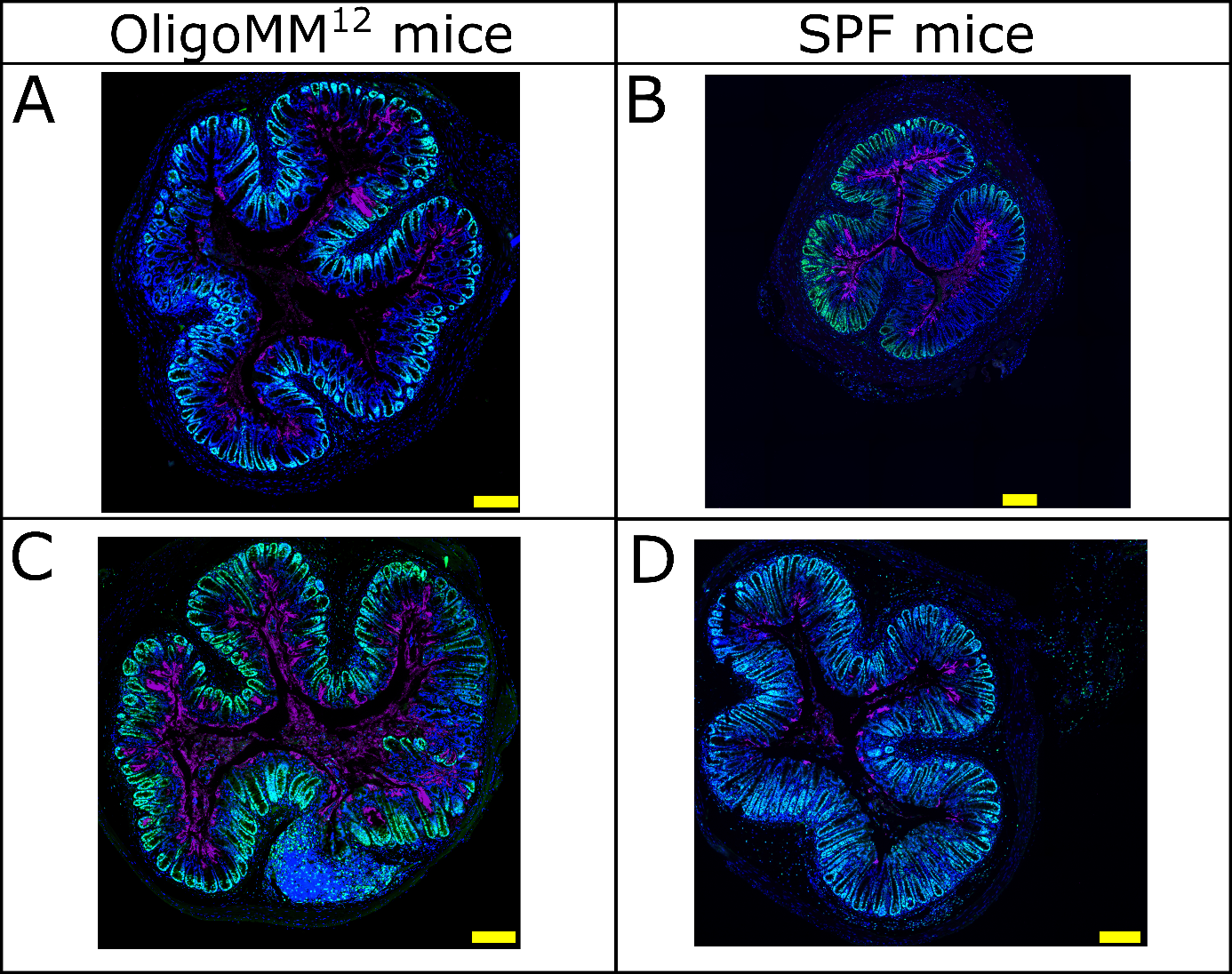


Figure 4: Representative images of the colon in C. rodentium infected OligoMM^12^ and SPF mice at endpoint (day 8). Immunofluorescence: C. rodentium (purple), proliferating cell nuclear antigen (green) and DAPI (blue). A) and C) are sections from OligoMM^12^ mice and B) and D) are sections from SPF mice. Similar C. rodentium densities in the feces were measured for A) (1.49*10^9 CFU/g) and B) (2.61*10^9 CFU/g), as well as for C) (2.67*10^8 CFU/g) and D) (3.01*10^8 CFU/g). Yellow scale bars correspond to 200 µm.


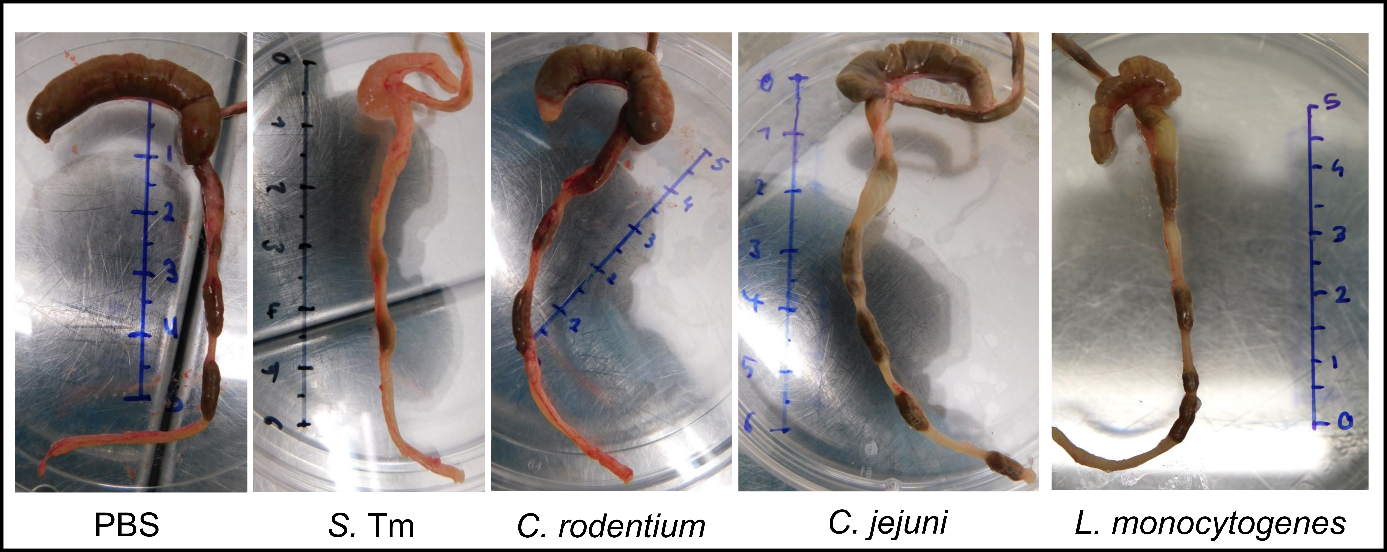


Figure 5: Representative macroscopic images of the cecum and colon of OligoMM^12^ mice infected with one of the four pathogens. The cecum and colon of a mock (PBS) treated mouse is shown for comparison. The unit of the blue scale bars is centimeter.


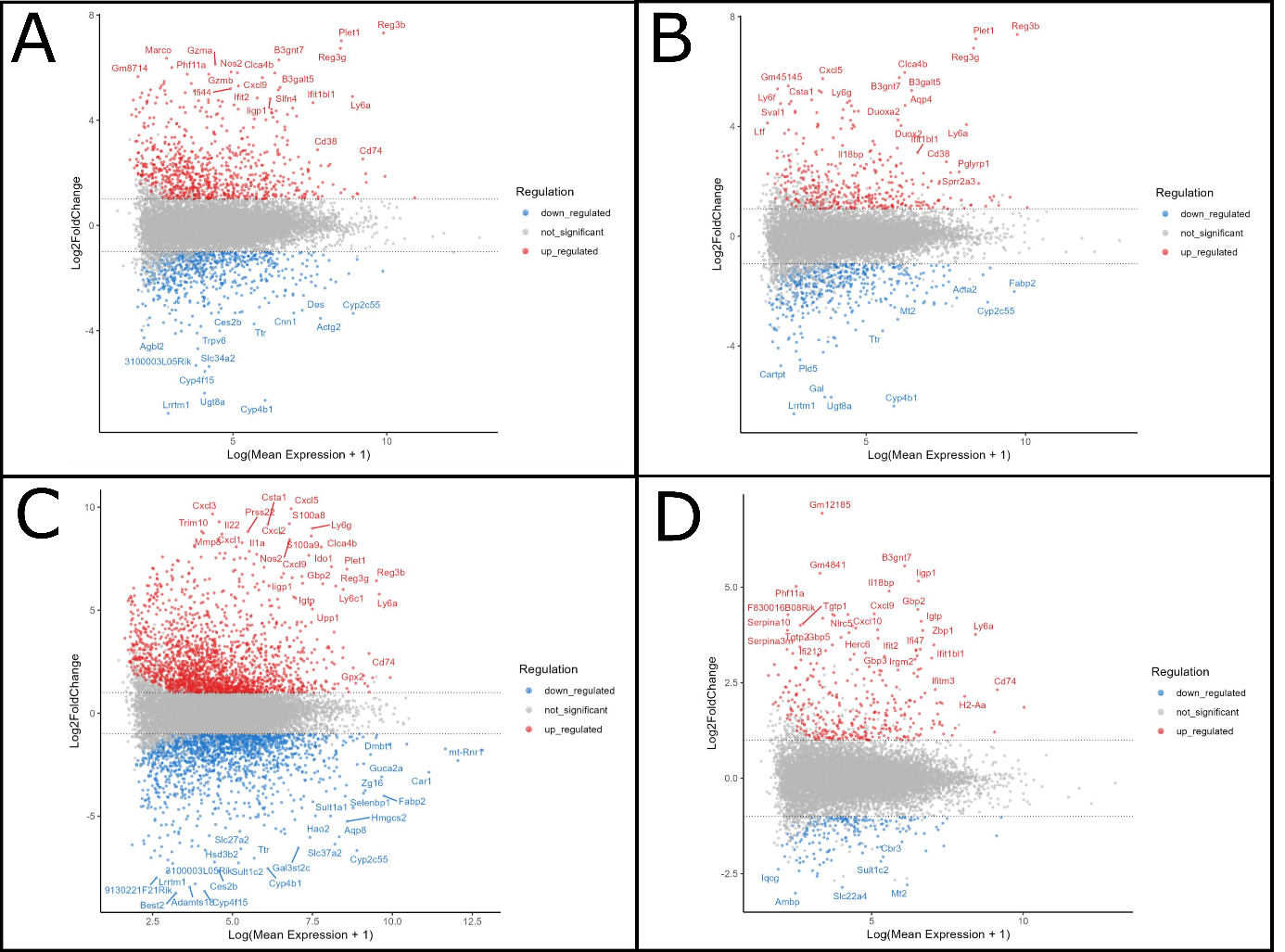


Figure 6: Differentially expressed genes in cecum tissue of OligoMM^12^ mice. A) C. jejuni vs PBS control. B) C. rodentium vs PBS control. C) S. Tm vs PBS control. D) L. monocytogenes vs PBS control. (Analysis by Alithea Genomics)


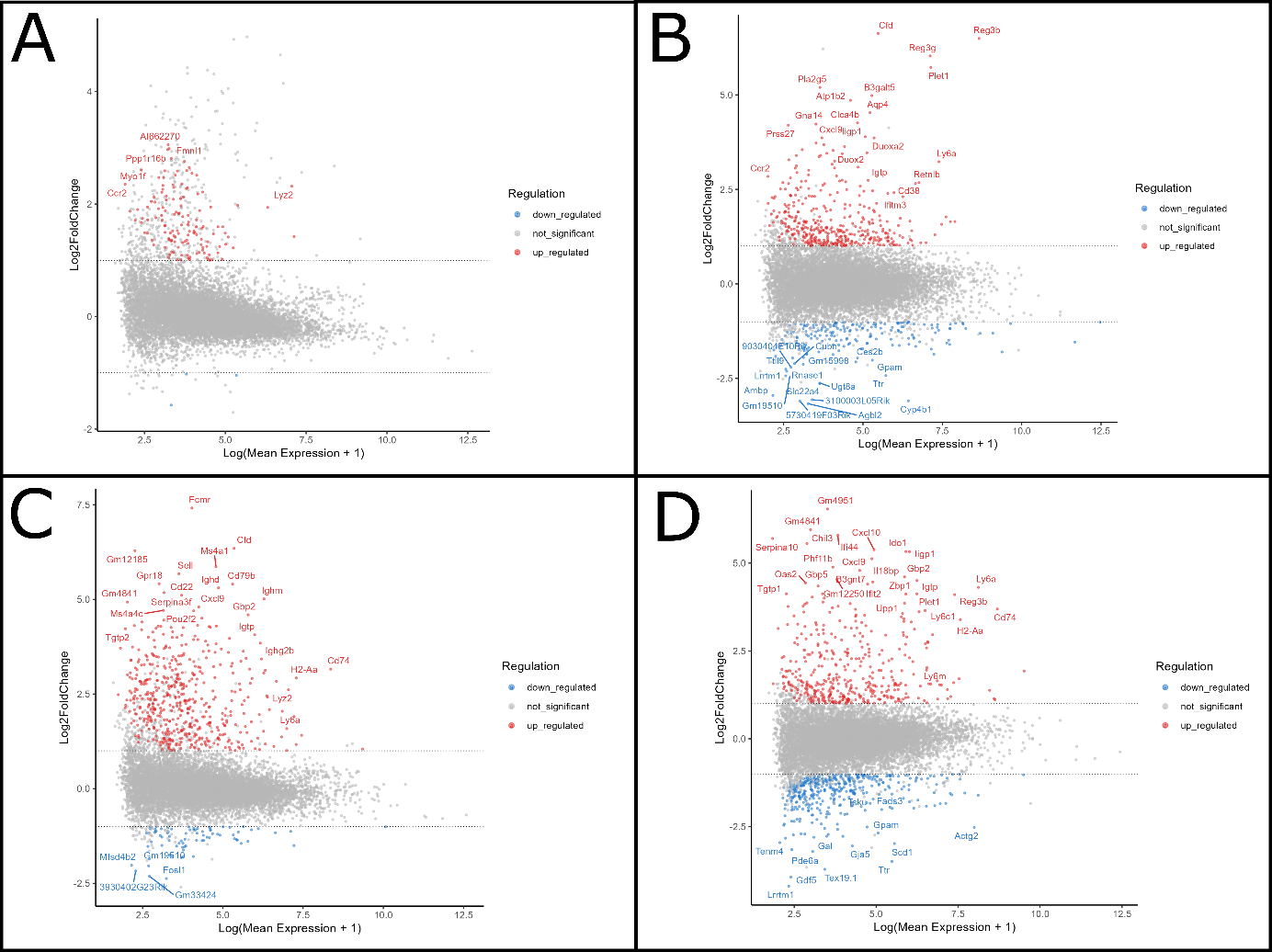


Figure 7: Differentially expressed genes in cecum tissue of SPF mice. A) C. jejuni vs PBS control. B) C. rodentium vs PBS control. C) S. Tm vs PBS control. D) L. monocytogenes vs PBS control. (Analysis by Alithea Genomics)


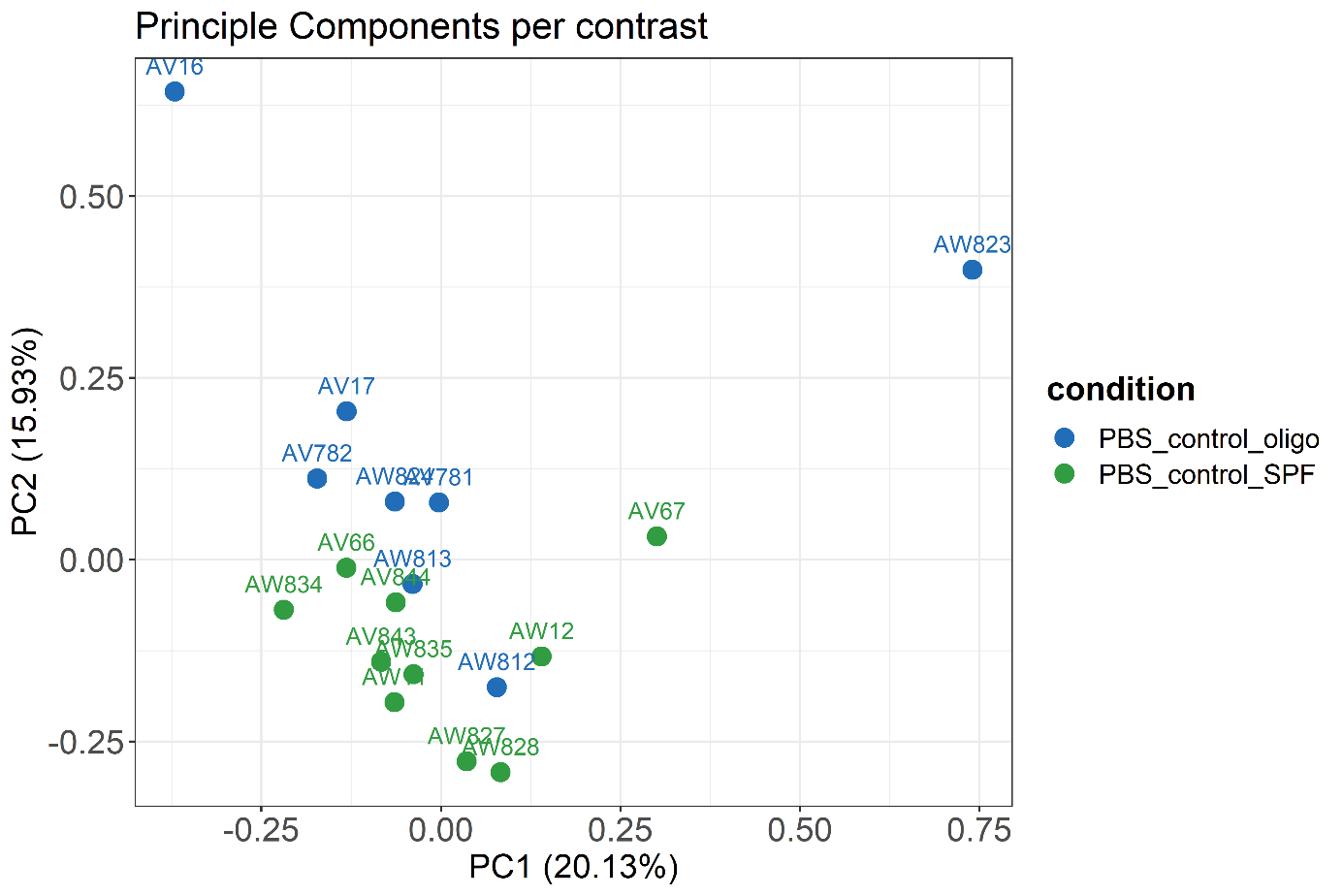


Figure 8: Principal component analysis of cecum tissue gene expression in mock (PBS) treated OligoMM^12^ and SPF mice.


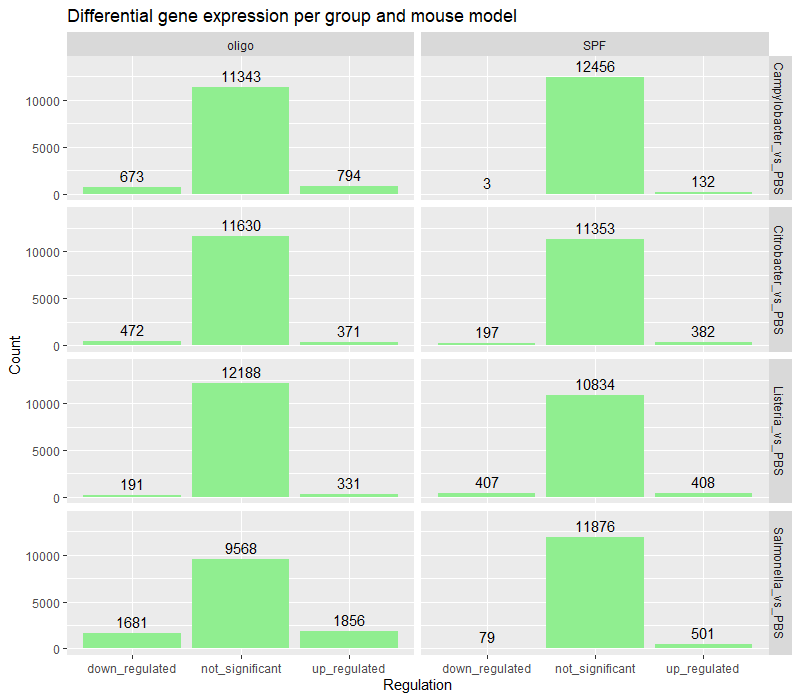


Figure 9: Number of detected genes in the cecum tissue of infected OligoMM^12^ and SPF mice.


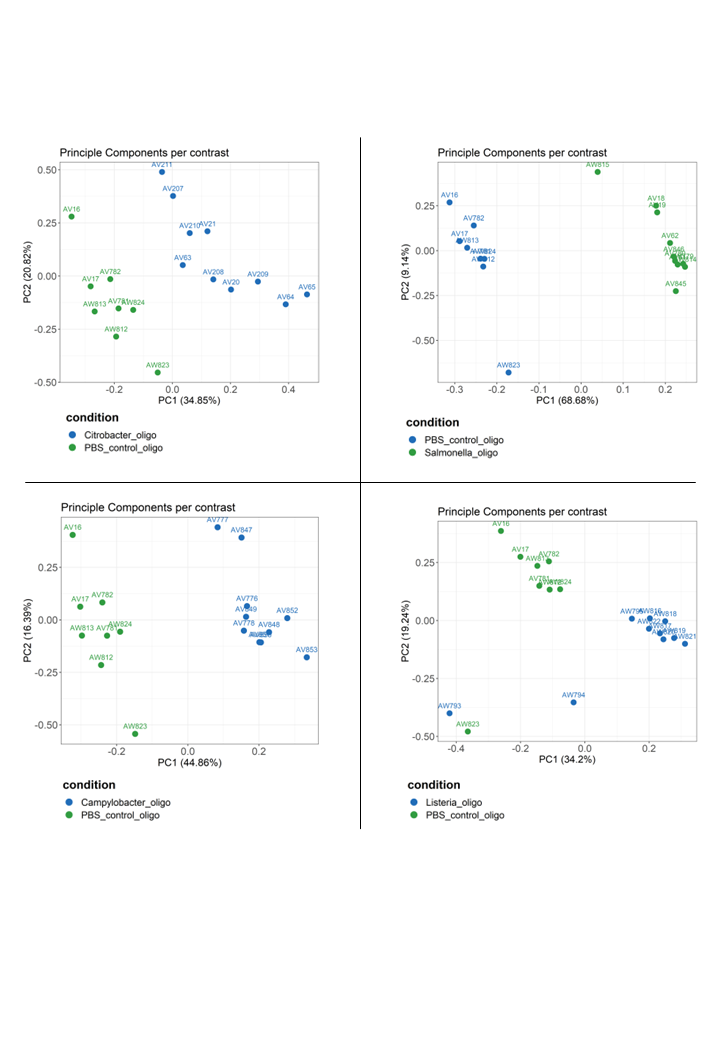


Figure 10: Principal component analysis of RNAseq data comparing infected animals with mock infected mice (all OligoMM^12^). (Analysis by Alithea Genomics)


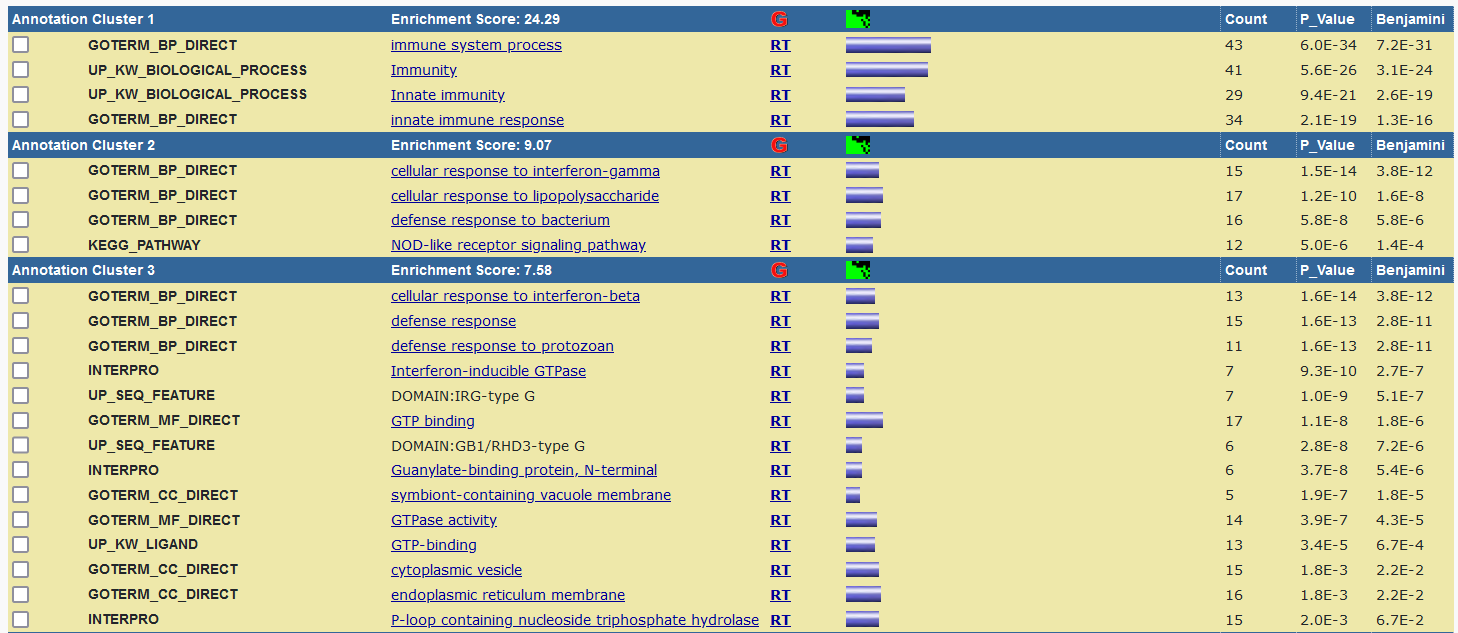


Figure 11: DAVID analysis with the genes (139) that are upregulated in the cecum tissue of OligoMM^12^ mice in all four pathogen groups. The list of Ensemble gene IDs were submitted to the DAVID online platform (default annotation categories) and “Functional Annotation Clustering” was chosen to obtain the results shown in this figure.


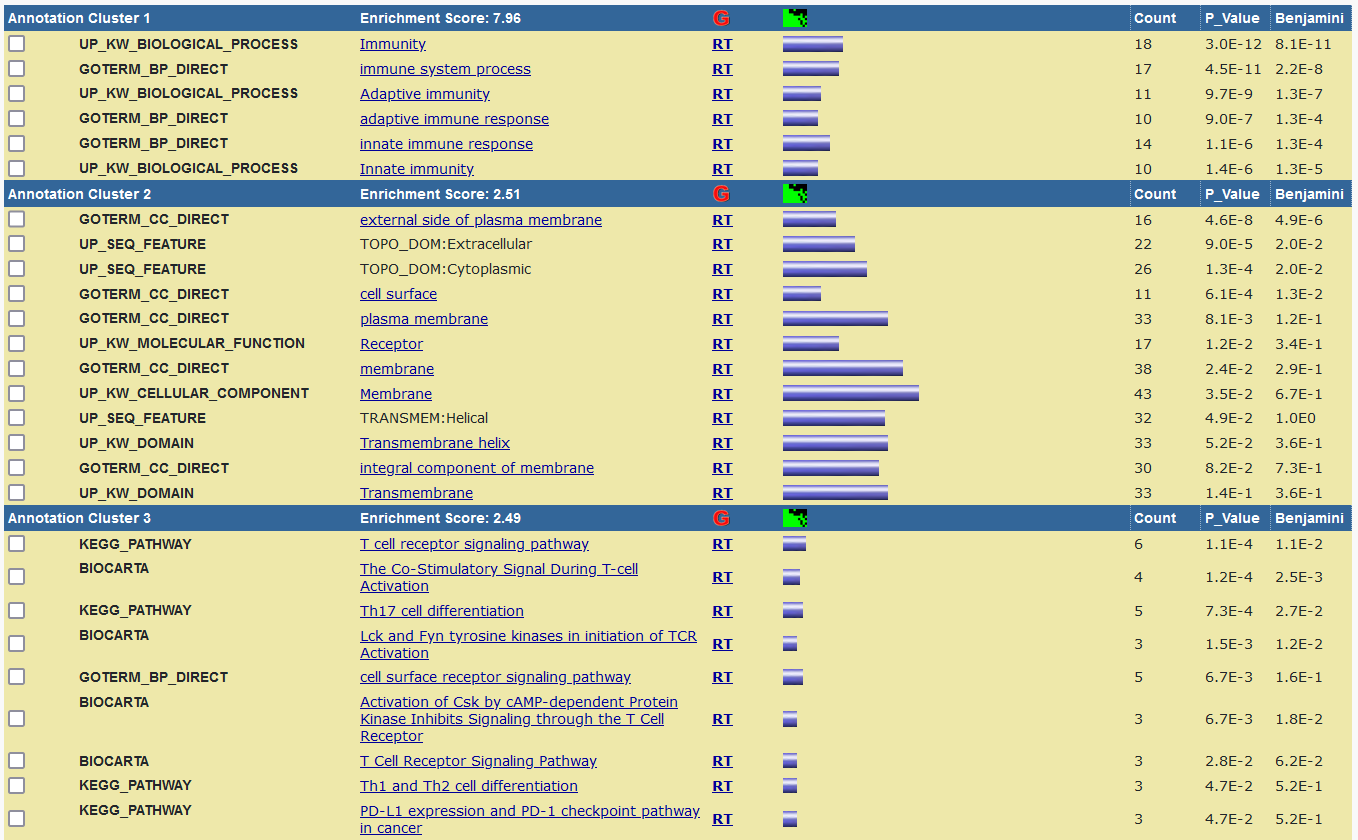


Figure 12: DAVID analysis with the genes (88) that are uniquely upregulated in the cecum tissue of OligoMM^12^ mice infected with C. jejuni. The list of Ensemble gene IDs was submitted to the DAVID online platform (default annotation categories) and “Functional Annotation Clustering” was chosen to obtain the results shown in this figure.


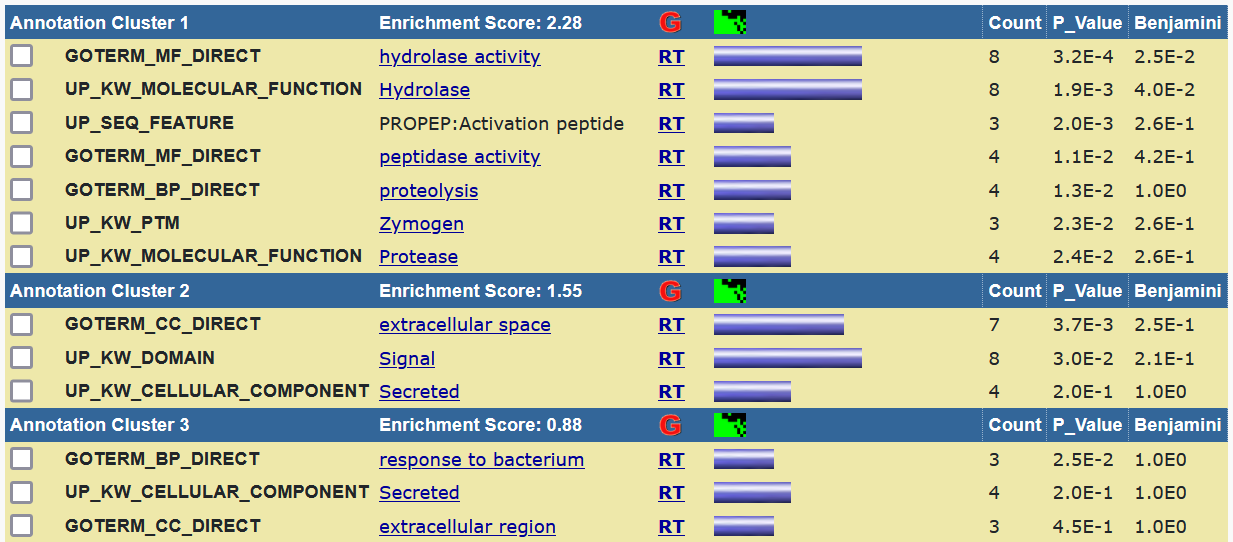


Figure 13: DAVID analysis with the genes (20) that are uniquely upregulated in the cecum tissue of OligoMM^12^ mice infected with C. rodentium. The list of Ensemble gene IDs was submitted to the DAVID online platform (default annotation categories) and “Functional Annotation Clustering” was chosen to obtain the results shown in this figure.


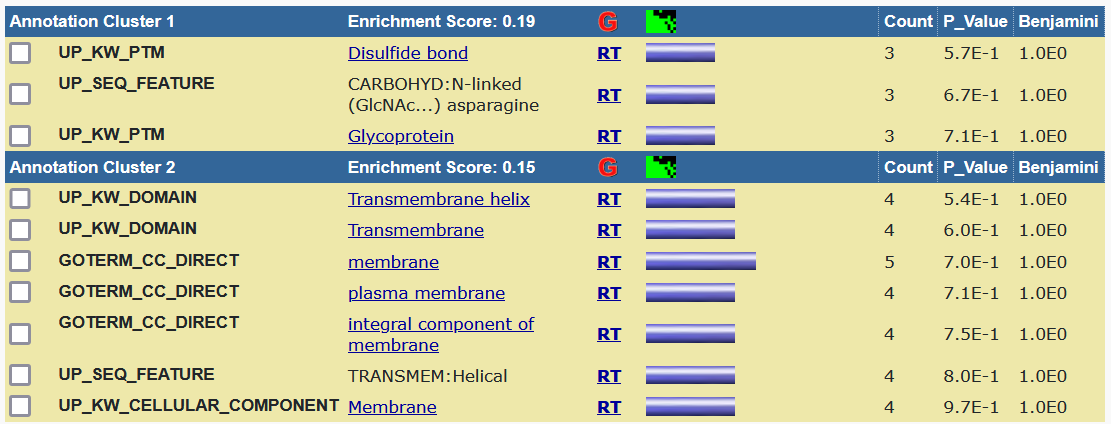


Figure 14: DAVID analysis with the genes (16) that are uniquely upregulated in the cecum tissue of OligoMM^12^ mice infected with L. monocytogenes. The list of Ensemble gene IDs was submitted to the DAVID online platform (default annotation categories) and “Functional Annotation Clustering” was chosen to obtain the results shown in this figure.


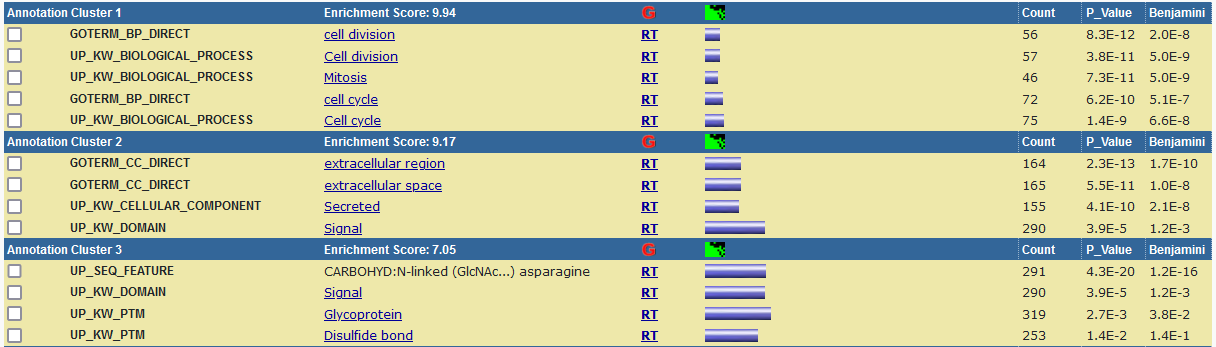


Figure 15: DAVID analysis with the genes (1132) that are uniquely upregulated in the cecum tissue of OligoMM^12^ mice infected with S. Tm. The list of Ensemble gene IDs was submitted to the DAVID online platform (default annotation categories) and “Functional Annotation Clustering” was chosen to obtain the results shown in this figure.


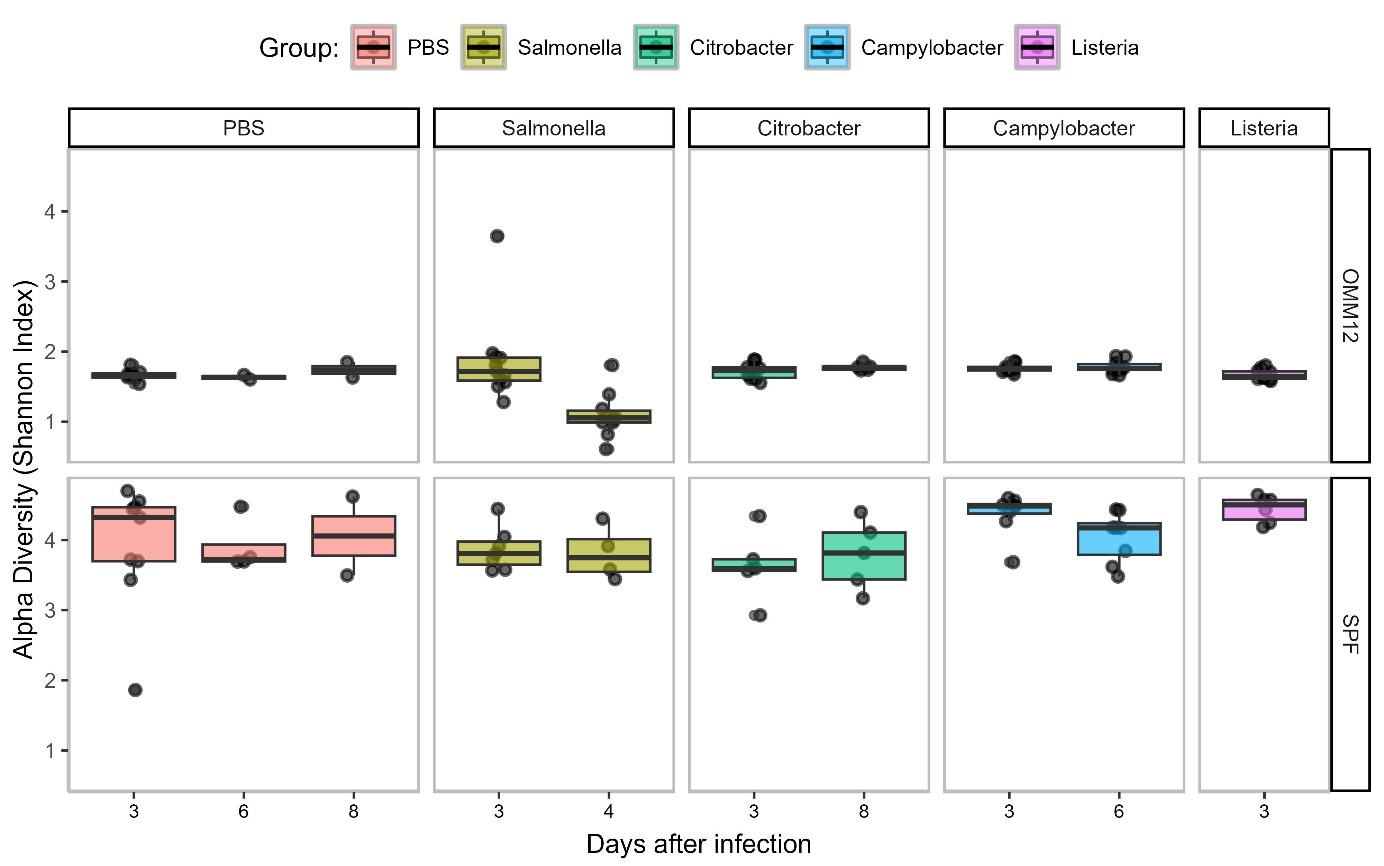


Figure 16: Alpha diversity of taxa in the feces of infected or mock (PBS) treated OligoMM^12^ and SPF mice.


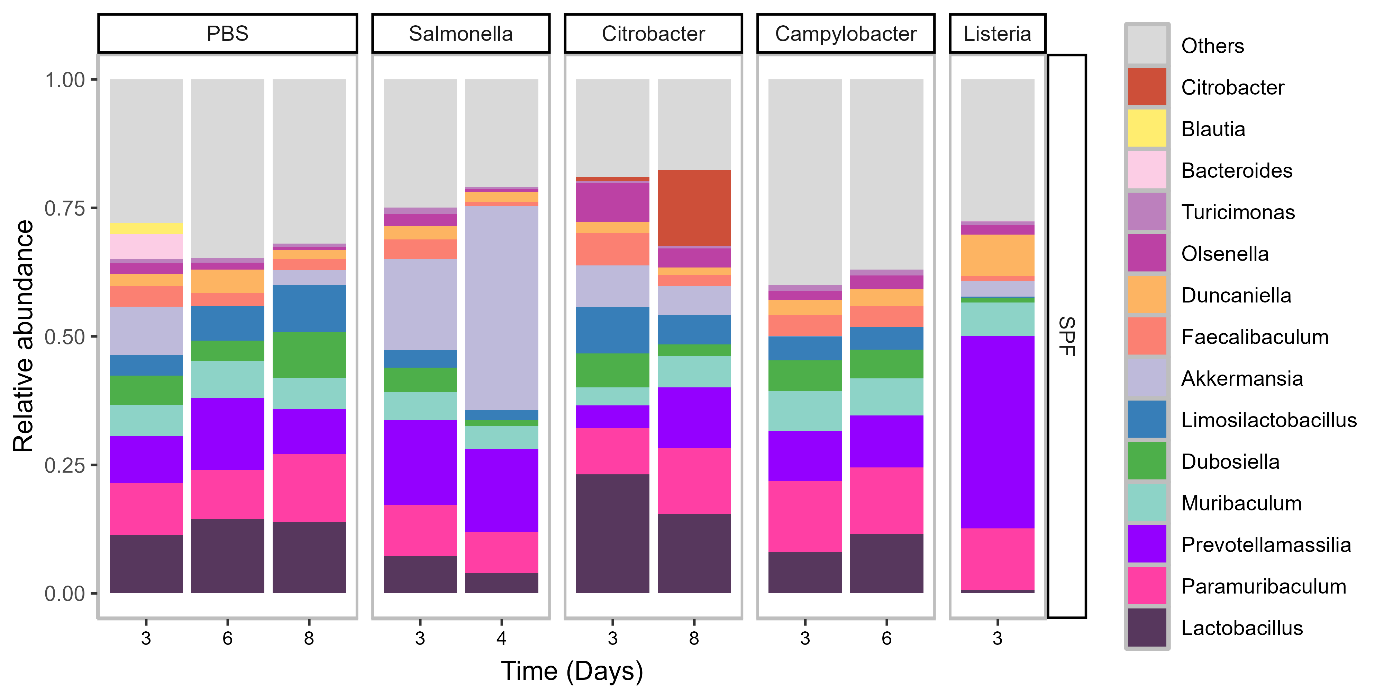


Figure 17: Relative abundance of genera in the SPF mice infected with one of the four pathogens or mock (PBS) treated.


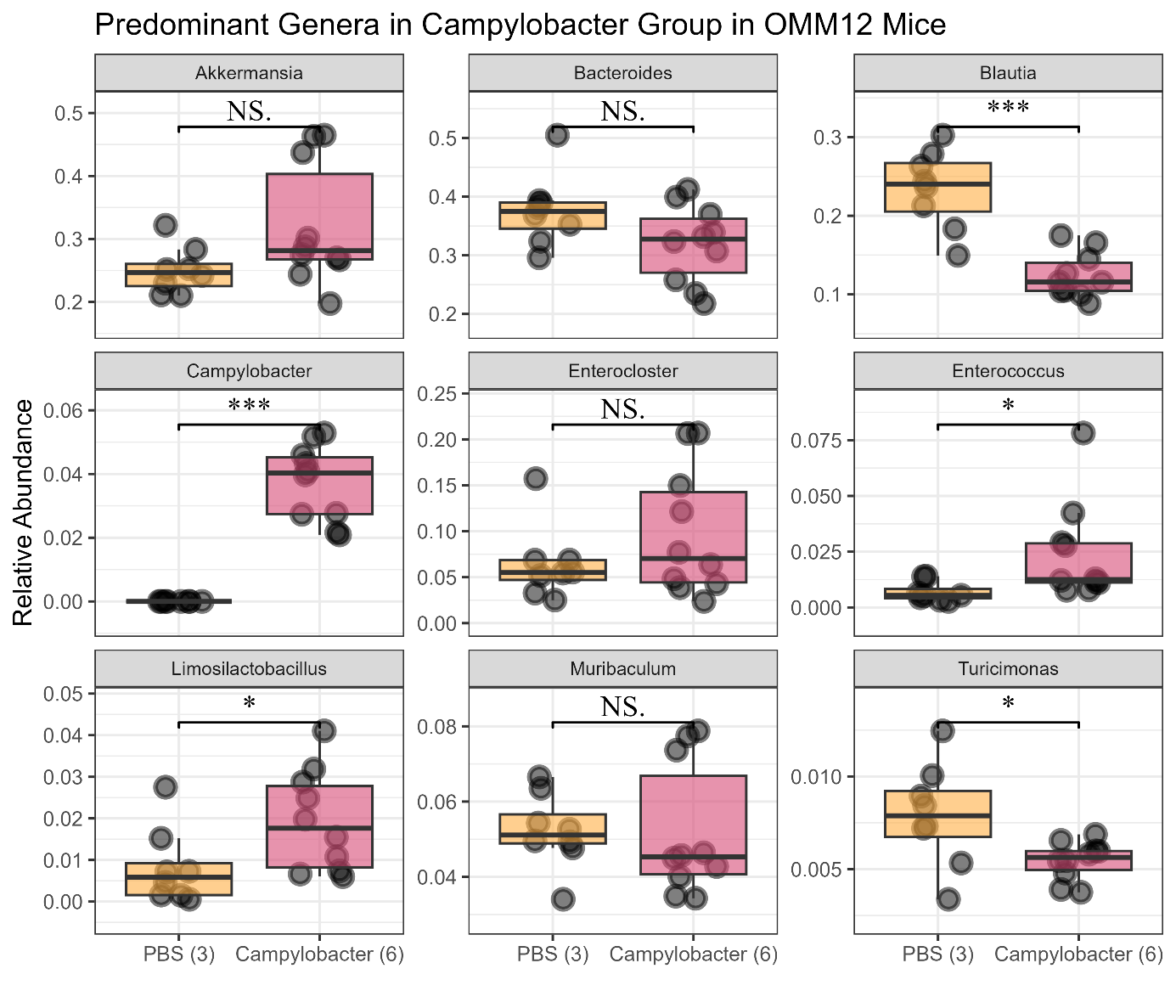


Figure 18: Predominant genera in C. jejuni infected OligoMM^12^ mice


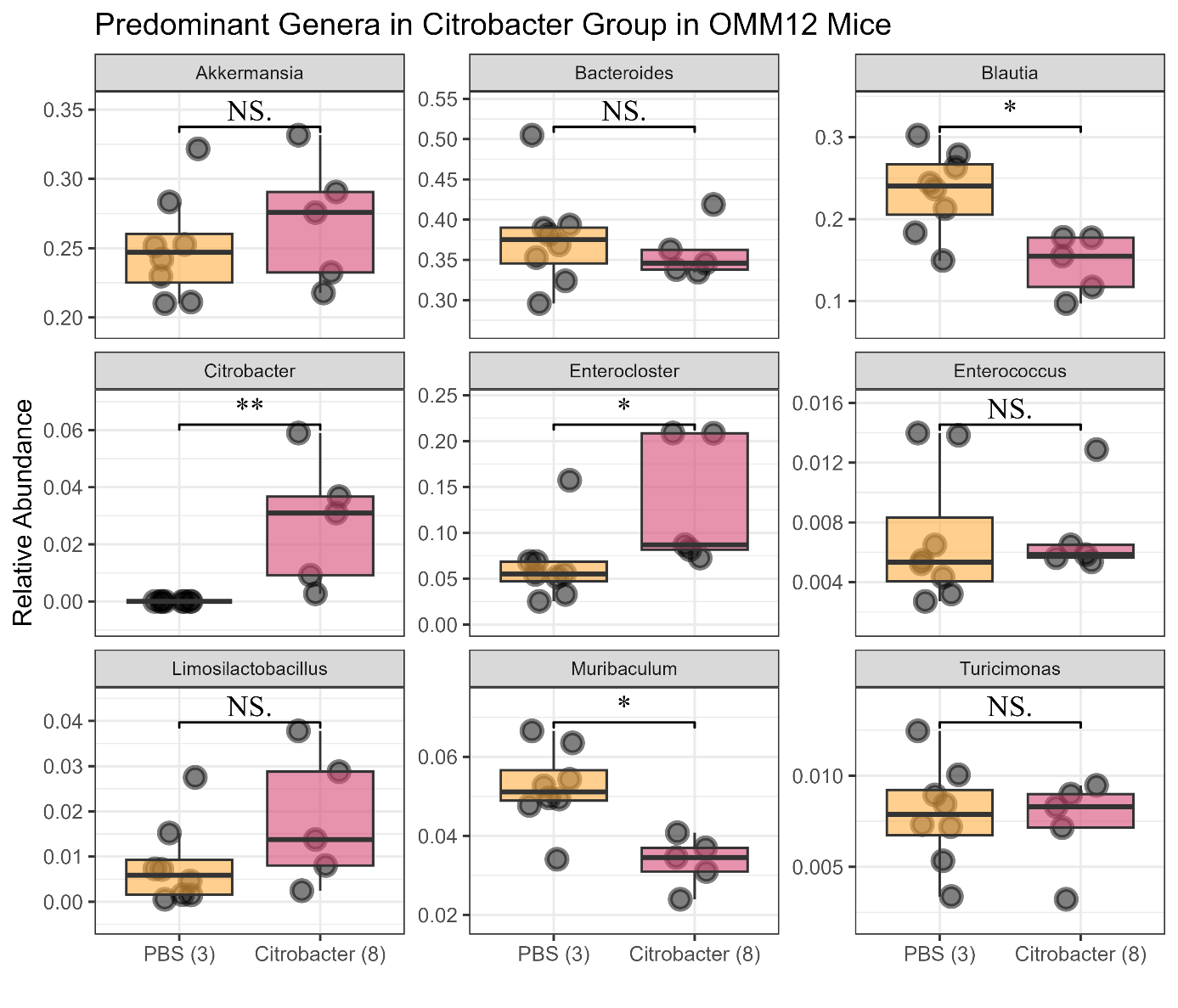


Figure 19: Predominant general in C. rodentium infected OligoMM^12^ mice.


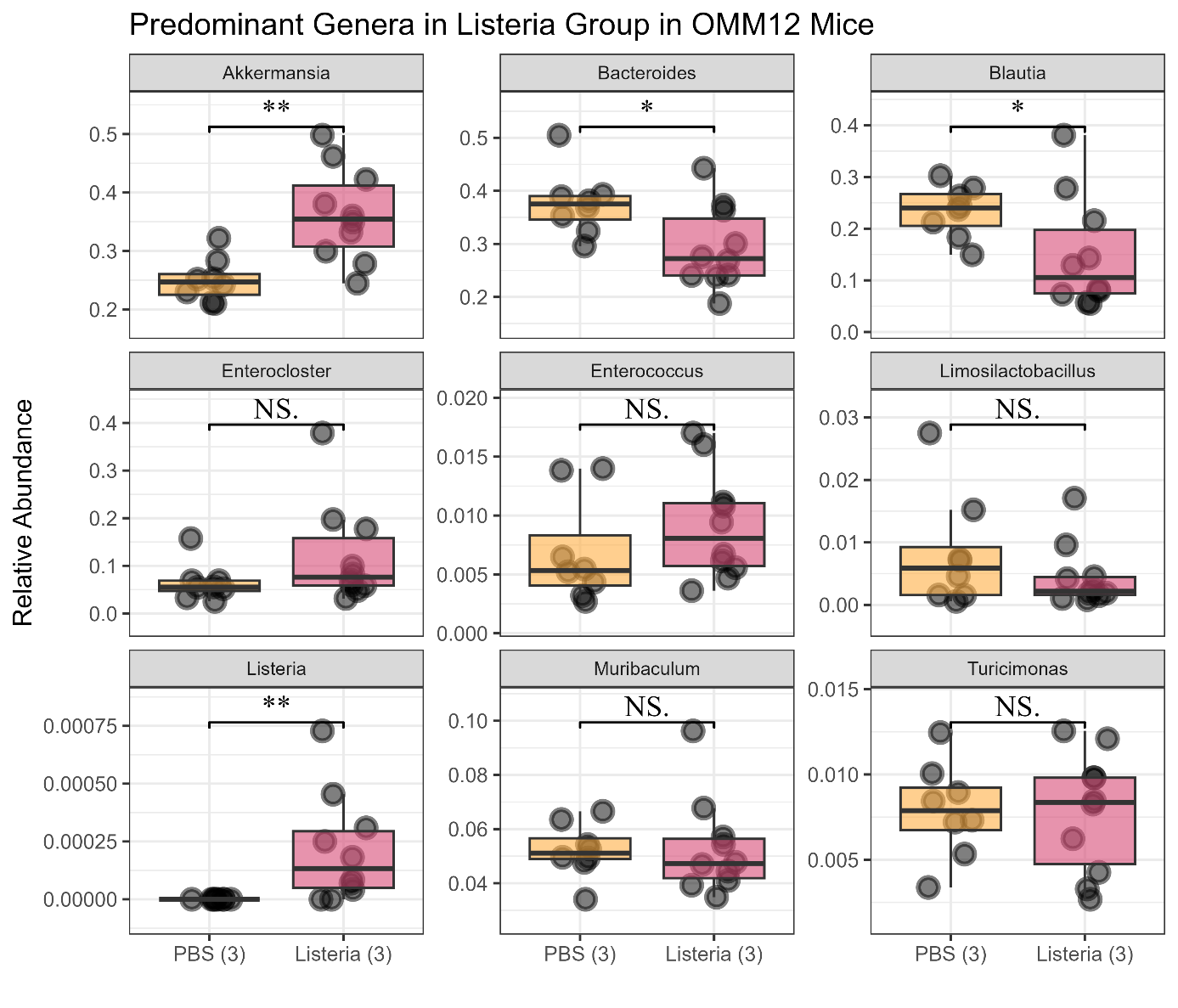


Figure 20: Predominant genera in L. monocytogenes infected OligoMM^12^ mice.


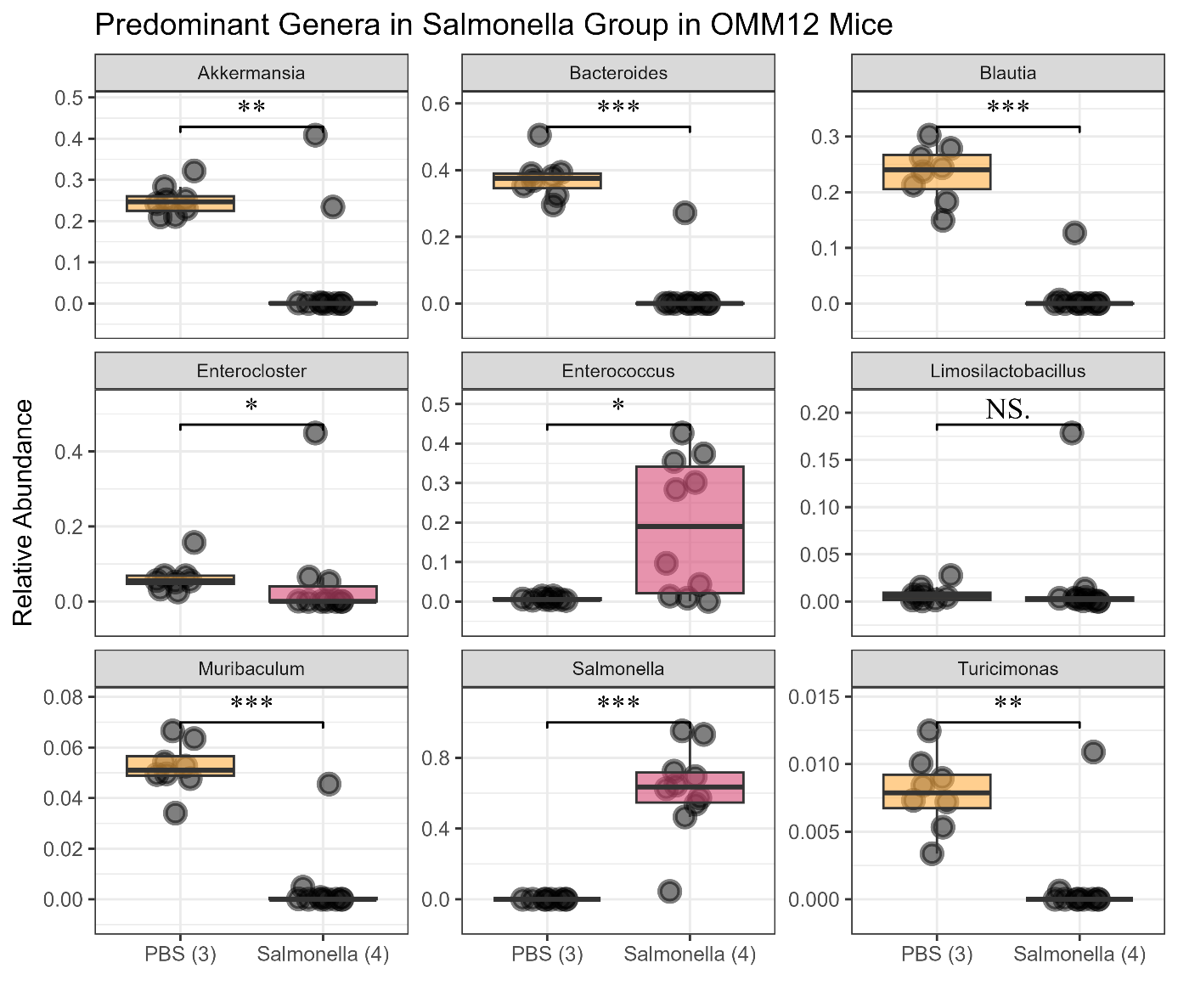


Figure 21: Predominant genera in S. Tm infected OligoMM^12^ mice.


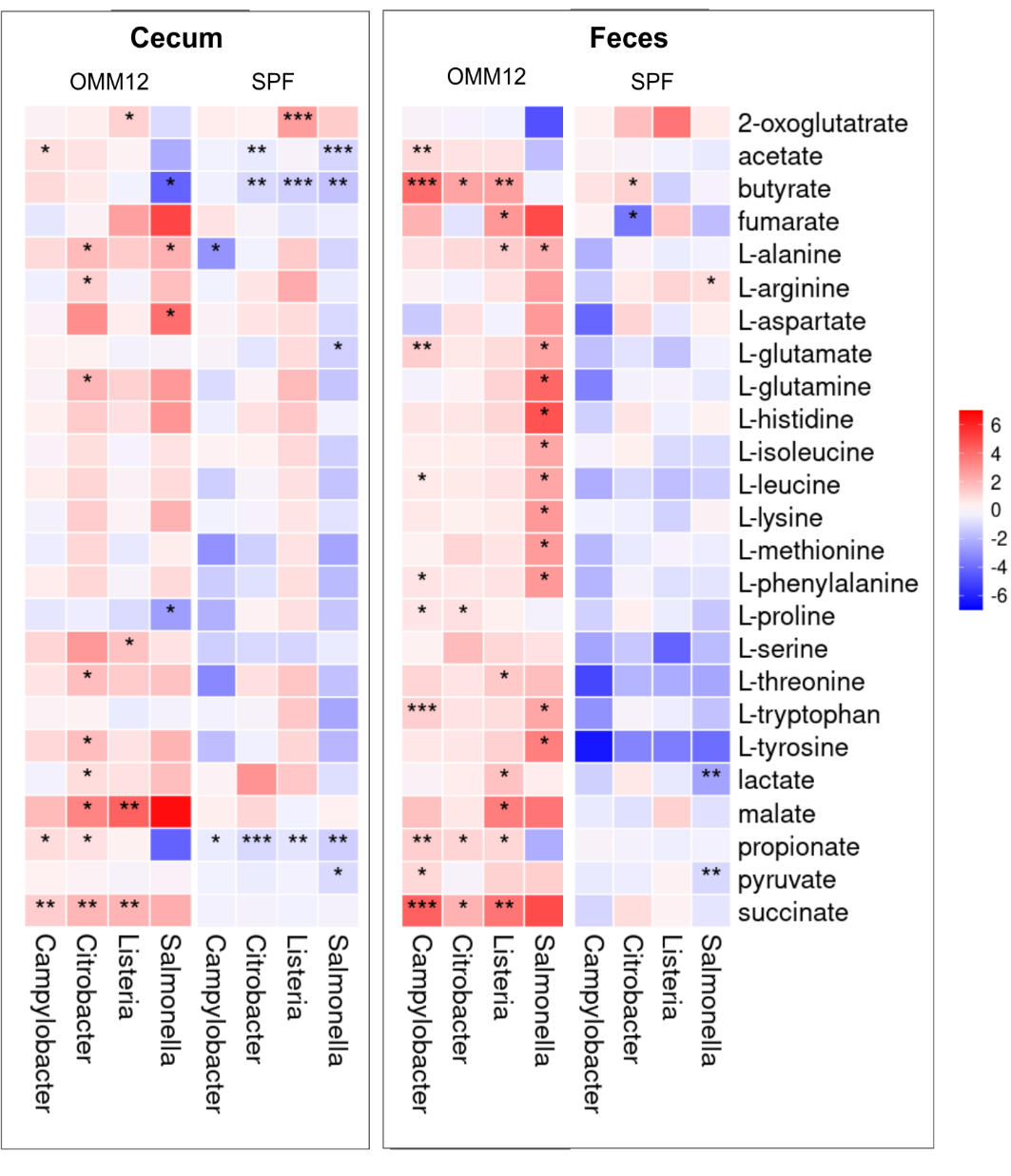


Figure 22: Heatmap of metabolite abundance in the feces and cecum of OligoMM^12^ and SPF mice relative to mock (PBS) treated mice. Color gradient indicates log2 fold change of average peak area of metabolites relative to PBS, and the stars indicate significance. (Wilcoxon test: p < 0.05 = *, p < 0.01 = **, p < 0.001 = ***, absence denotes non-significant values)


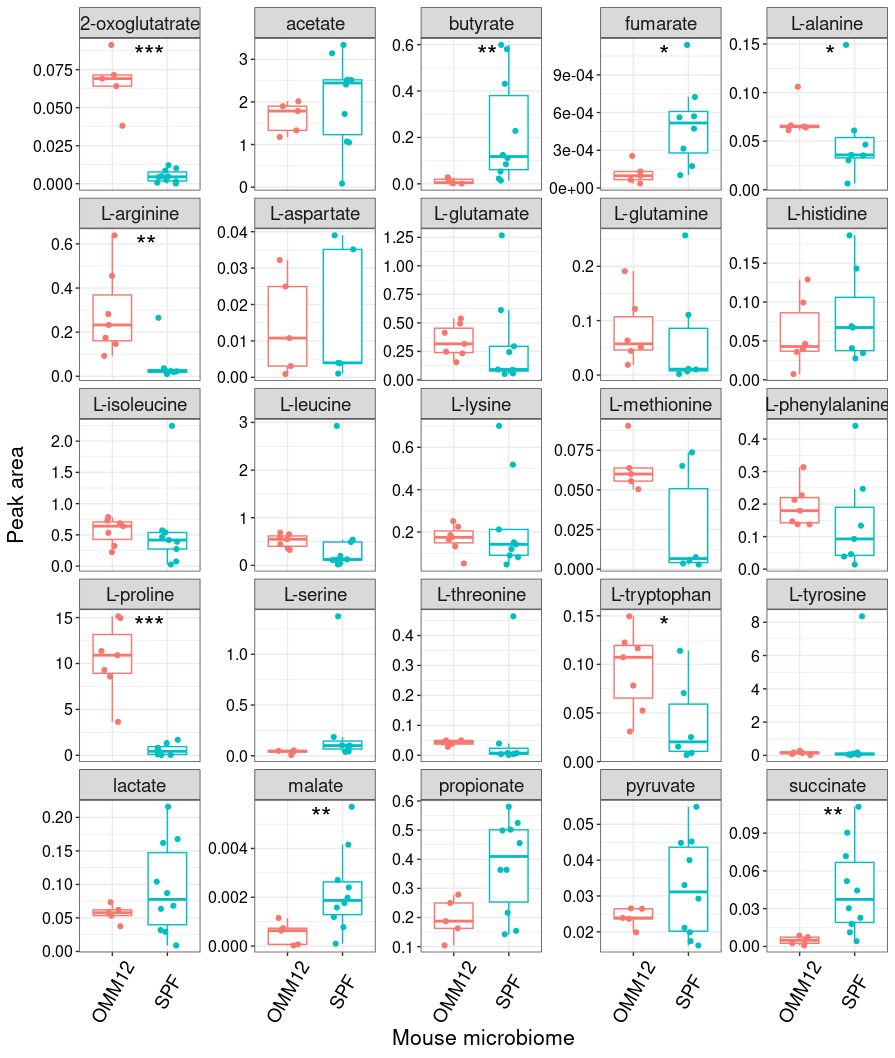


Figure 23: Targeted metabolomics data of feces of the mock (PBS) treated OligoMM^12^ and SPF mice (Wilcoxon test: p < 0.05 = *, p < 0.01 = **, p < 0.001 = ***, absence denotes non-significant values)


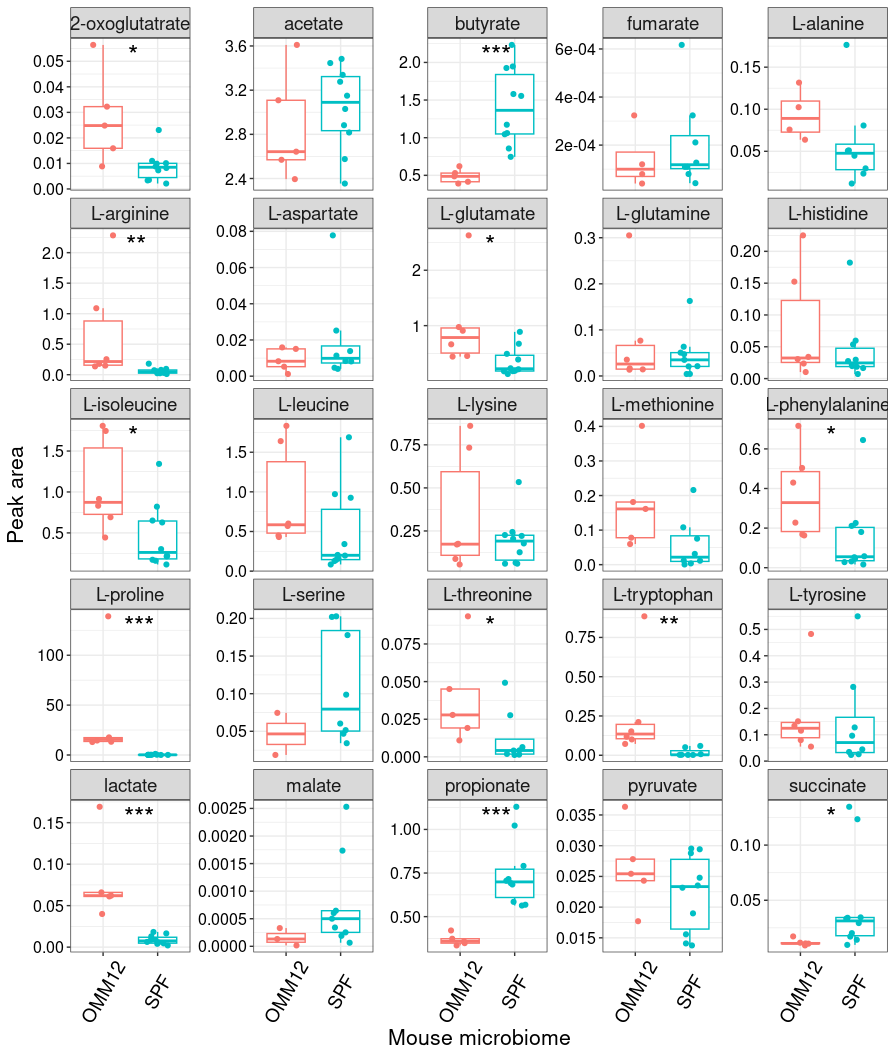


Figure 24: Targeted metabolomics data of the mock (PBS) treated OligoMM^12^ and SPF mice in cecum (Wilcoxon test: p < 0.05 = *, p < 0.01 = **, p < 0.001 = ***, absence denotes non-significant values)


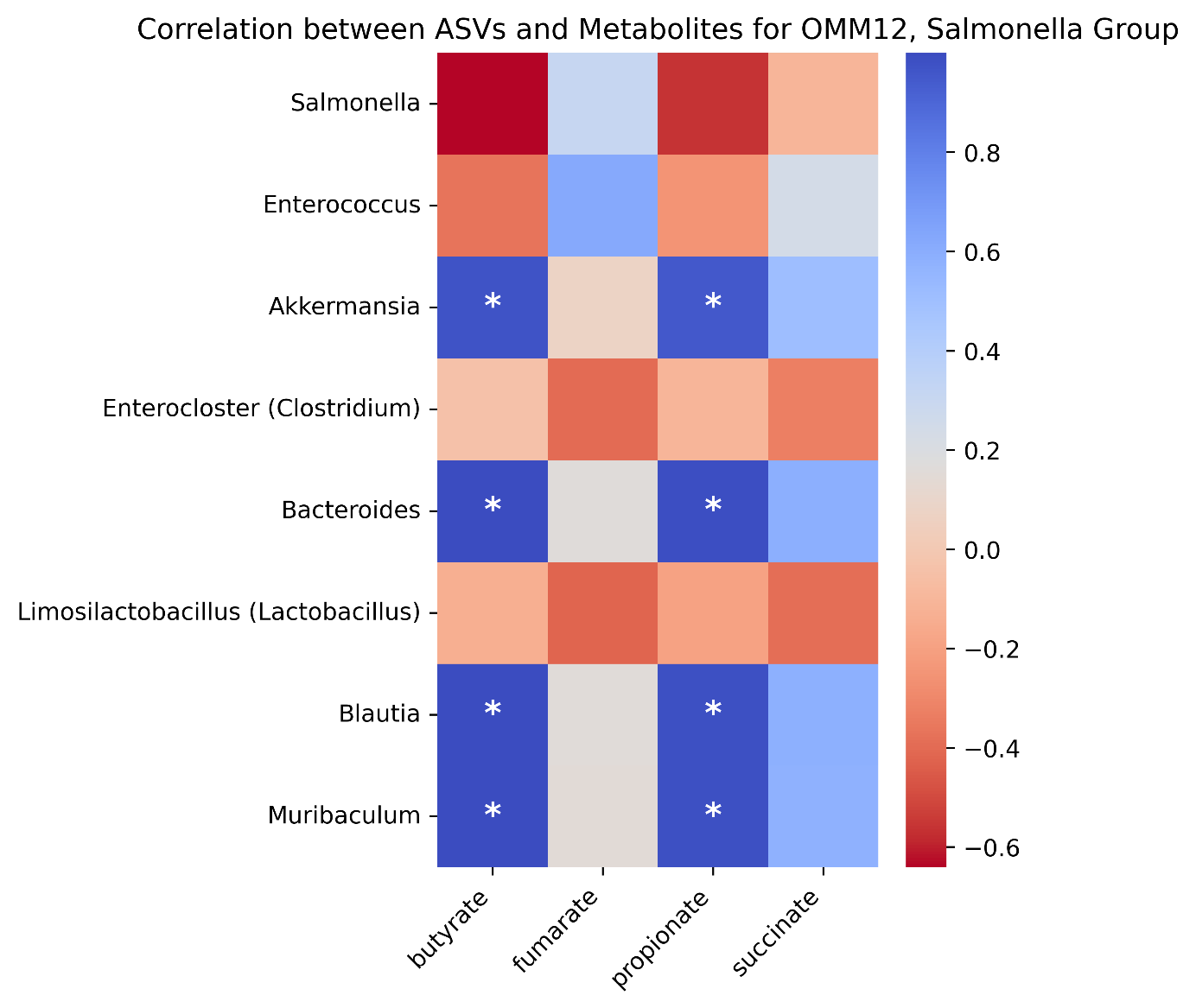


Figure 25: Correlation of the abundance of genera and selected metabolites in S. Tm infected OligoMM^12^ mice.

Table 1: Top20 up regulated genes in OligoMM^12^ mice infected with the four pathogens.

| ***C. jejuni*** | | ***C. rodentium*** | | ***L. monocytogenes*** | | ***S*. Tm** | |
| --- | --- | --- | --- | --- | --- | --- | --- |
| Gene name | Log2 fold  change | Gene name | Log2 fold  change | Gene name | Log2 fold  change | Gene name | Log2 fold  change |
| Reg3b | 7.32 | Reg3b | 7.35 | Gm12185 | 6.94 | Cxcl5 | 9.92 |
| Plet1 | 7.02 | Plet1 | 7.19 | B3gnt7 | 5.56 | Cxcl3 | 9.67 |
| Reg3g | 6.73 | Reg3g | 6.85 | Gm4841 | 5.37 | Il22 | 9.29 |
| Marco | 6.35 | Clca4b | 5.96 | Iigp1 | 5.16 | S100a8 | 9.20 |
| B3gnt7 | 6.29 | B3gnt7 | 5.78 | Phf11a | 5.02 | Csta1 | 9.16 |
| Gzma | 6.13 | Cxcl5 | 5.74 | Il18bp | 4.90 | Ly6g | 8.97 |
| Phf11a | 6.00 | Gm45145 | 5.47 | Gbp2 | 4.42 | Trim10 | 8.83 |
| Gzmb | 5.84 | Ly6f | 5.37 | Cxcl9 | 4.30 | Prss22 | 8.82 |
| Nos2 | 5.80 | B3galt5 | 5.31 | Gm12250 | 4.29 | Cxcl2 | 8.74 |
| Clca4b | 5.79 | Csta1 | 5.30 | Art2a | 4.29 | Mmp8 | 8.74 |
| Gm4841 | 5.75 | Prss27 | 5.25 | F830016B08Rik | 4.28 | Cxcl1 | 8.70 |
| Gm4951 | 5.75 | Gm14137 | 4.97 | Gm4951 | 4.26 | S100a9 | 8.60 |
| Gm8714 | 5.66 | Ly6g | 4.93 | Serpina3f | 4.19 | Nos2 | 8.42 |
| Cxcl9 | 5.62 | Plet1os | 4.91 | Igtp | 4.11 | Il1a | 8.28 |
| Gm45145 | 5.48 | Nos2 | 4.87 | Tgtp1 | 4.05 | Ifng | 8.14 |
| Gm14137 | 5.40 | Sval1 | 4.83 | Tgtp2 | 4.01 | Clec4e | 8.08 |
| Lrrc4 | 5.38 | Cxcl1 | 4.82 | Phf11b | 3.99 | Ptx3 | 8.07 |
| Plet1os | 5.31 | Aqp4 | 4.76 | Ifi44 | 3.93 | Clca4b | 8.06 |
| Ly6g | 5.30 | Cxcl2 | 4.75 | Cxcl10 | 3.89 | Ly6i | 7.86 |
| B3galt5 | 5.25 | Atp1b2 | 4.61 | Serpina10 | 3.87 | Gm4841 | 7.77 |

Table 2: Top20 down regulated genes in OligoMM^12^ mice infected with one of the four pathogens.

| ***C. jejuni*** | | ***C. rodentium*** | | ***L. monocytogenes*** | | ***S*. Tm** | |
| --- | --- | --- | --- | --- | --- | --- | --- |
| Gene name | Log2 fold  change | Gene name | Log2 fold  change | Gene name | Log2 fold  change | Gene name | Log2 fold  change |
| Lrrtm1 | -7.15 | Lrrtm1 | -6.47 | Ambp | -3.01 | Best2 | -8.74 |
| Cyp4b1 | -6.65 | Cyp4b1 | -6.19 | Slc22a4 | -2.86 | Cyp4f15 | -8.64 |
| Ugt8a | -6.38 | Ugt8a | -5.87 | Mt2 | -2.79 | Adamts18 | -8.44 |
| Cyp4f15 | -5.55 | Gal | -5.86 | Nmnat2 | -2.57 | 3100003L05Rik | -8.29 |
| Slc34a2 | -5.37 | Cartpt | -4.72 | Pld5 | -2.50 | 9130221F21Rik | -7.99 |
| 3100003L05Rik | -5.32 | Pld5 | -4.51 | Gm33424 | -2.43 | Lrrtm1 | -7.68 |
| Trpv6 | -4.69 | 3100003L05Rik | -4.23 | Lrrtm1 | -2.42 | Ces2b | -7.64 |
| Agbl2 | -4.27 | Ttll9 | -4.20 | Iqcg | -2.38 | Cyp4b1 | -7.54 |
| Pld5 | -4.11 | Ambp | -4.07 | Asb4 | -2.27 | Pld5 | -7.29 |
| Gal | -4.09 | Slc34a2 | -3.99 | 5430427M07Rik | -2.21 | Sult1c2 | -7.27 |
| Krt17 | -4.02 | Col8a2 | -3.87 | Best2 | -2.21 | Hsd3b2 | -7.22 |
| Ces2b | -4.01 | Slc22a4 | -3.85 | Gm15972 | -2.18 | Ugt2b36 | -7.12 |
| Gm45004 | -3.99 | Pde6a | -3.85 | Sult1c2 | -2.17 | Ttr | -7.03 |
| Slc22a4 | -3.84 | Adamts18 | -3.78 | Rassf10 | -2.15 | 5430427M07Rik | -7.01 |
| Pde6a | -3.79 | Susd4 | -3.76 | Cyp2c68 | -2.15 | Pbld1 | -6.92 |
| Igfbp2 | -3.78 | Vip | -3.72 | Per3 | -2.12 | Odf3b | -6.73 |
| Ttr | -3.75 | Pbld1 | -3.70 | Hoga1 | -2.11 | Afm | -6.67 |
| Best2 | -3.71 | Cyp4f15 | -3.69 | Slc16a11 | -2.09 | Cyp2c55 | -6.67 |
| Pbld1 | -3.70 | Nmnat2 | -3.69 | Cbr3 | -2.06 | Ugt8a | -6.64 |
| Odf3b | -3.59 | Calb2 | -3.56 | Susd4 | -2.06 | Slc27a2 | -6.58 |
