# Supplementary material for: Comparing Campylobacter jejuni to three other enteric pathogens in OligoMM12 mice reveals pathogen-specific host and microbiota responses
Source: Gut Microbes. 2025 Jan 21;17(1):2447832. doi: 10.1080/19490976.2024.2447832 (PMC12931697; doi:10.1080/19490976.2024.2447832)
Supplement: Supplemental Material [file KGMI_A_2447832_SM7659.zip › 2447832/20250112_four_path_supplements_revised.pdf]

Comparing *Campylobacter jejuni* to three other enteric pathogens in OligoMM^{12} mice reveals pathogen specific host and microbiota responses

Supplements

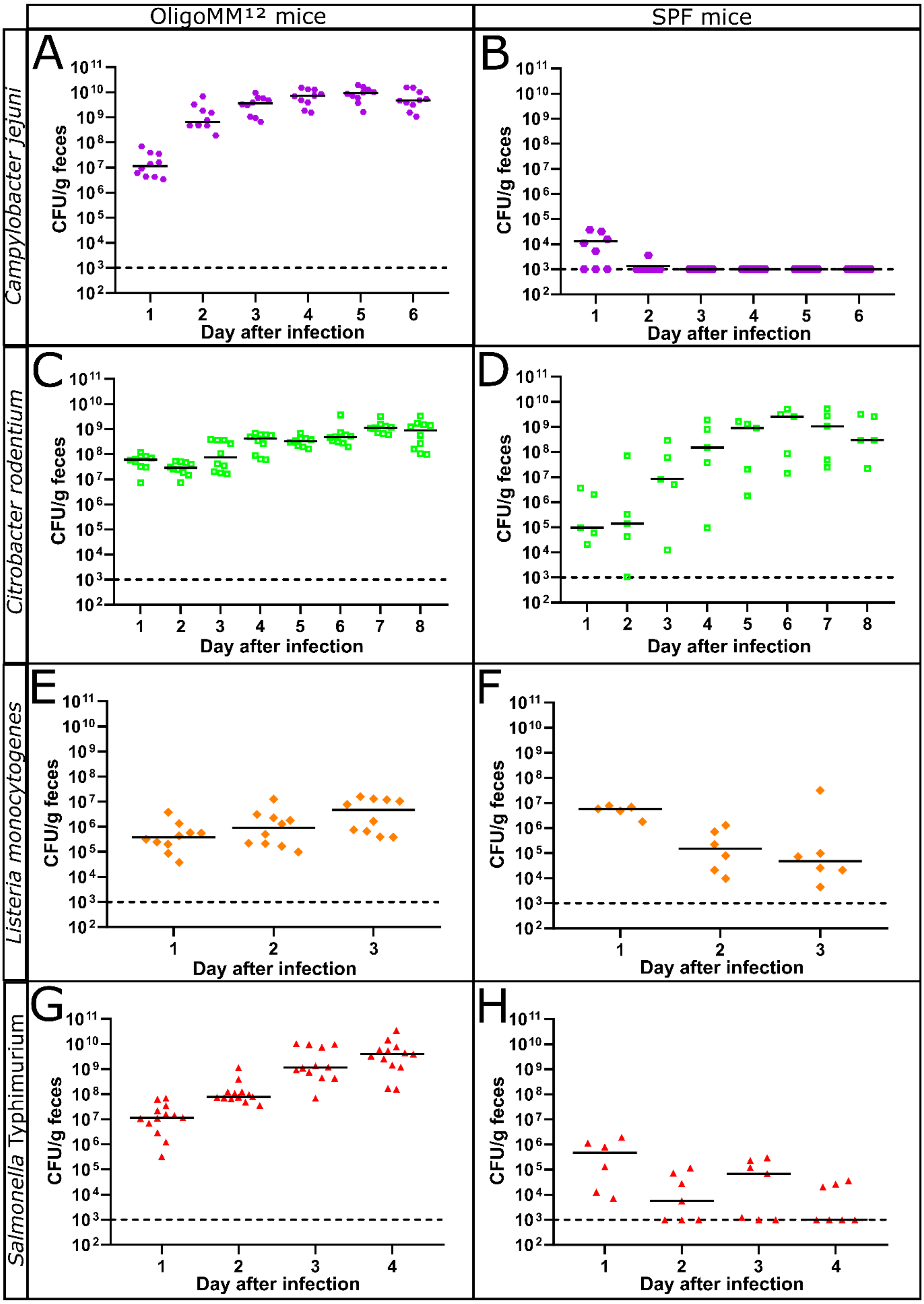

Figure 1: Fecal densities of the four pathogens in OligoMM¹² and SPF mice.

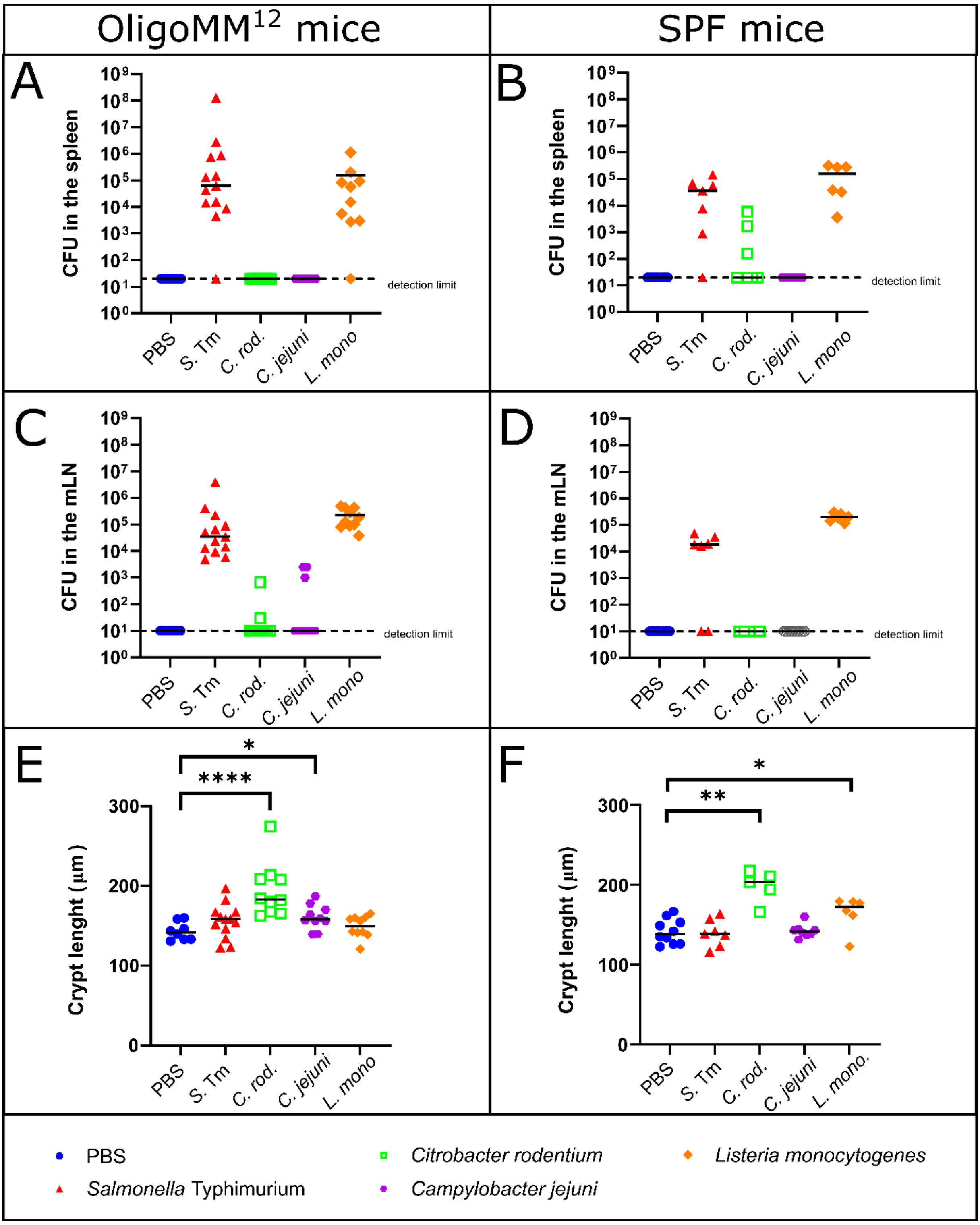

Figure 2: Colony forming units (CFU) in spleen and mesenteric lymph nodes (mLN) of OligoMM[12] and SPF mice as well as crypt length in the distal colon.

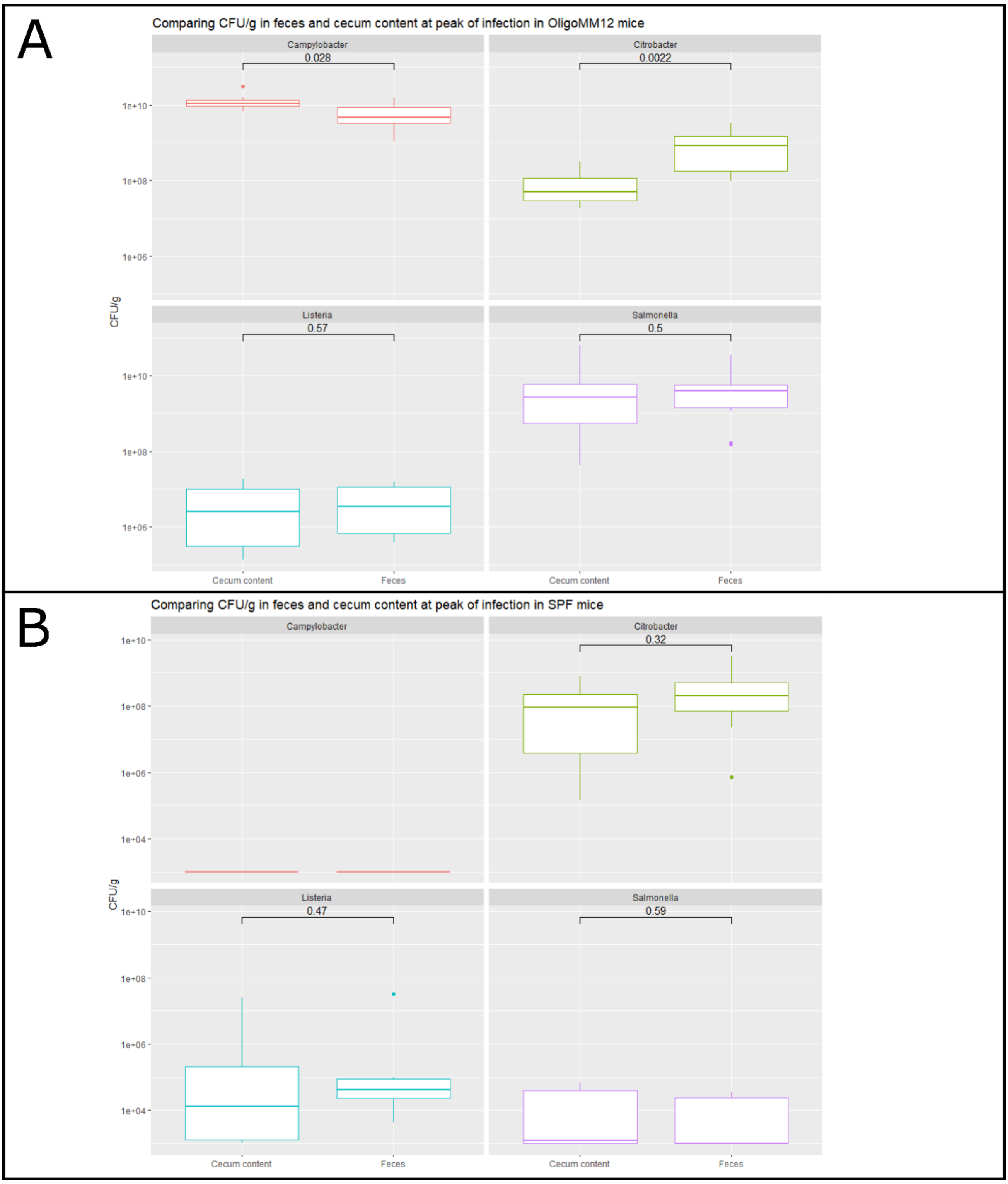

Figure 3: Comparing CFU/g of feces and CFU/g of cecum content on the last day of the experiment for all four pathogen groups in the two mouse models. A) CFU/g in feces or cecum content on the last day of infection in OligoMM^{12} mice. B) CFU/g in feces or cecum content on the last day of infection in SPF mice. Detection limit: 1000 CFU/g. Statistics: Wilcox test with approximate p-value calculation.

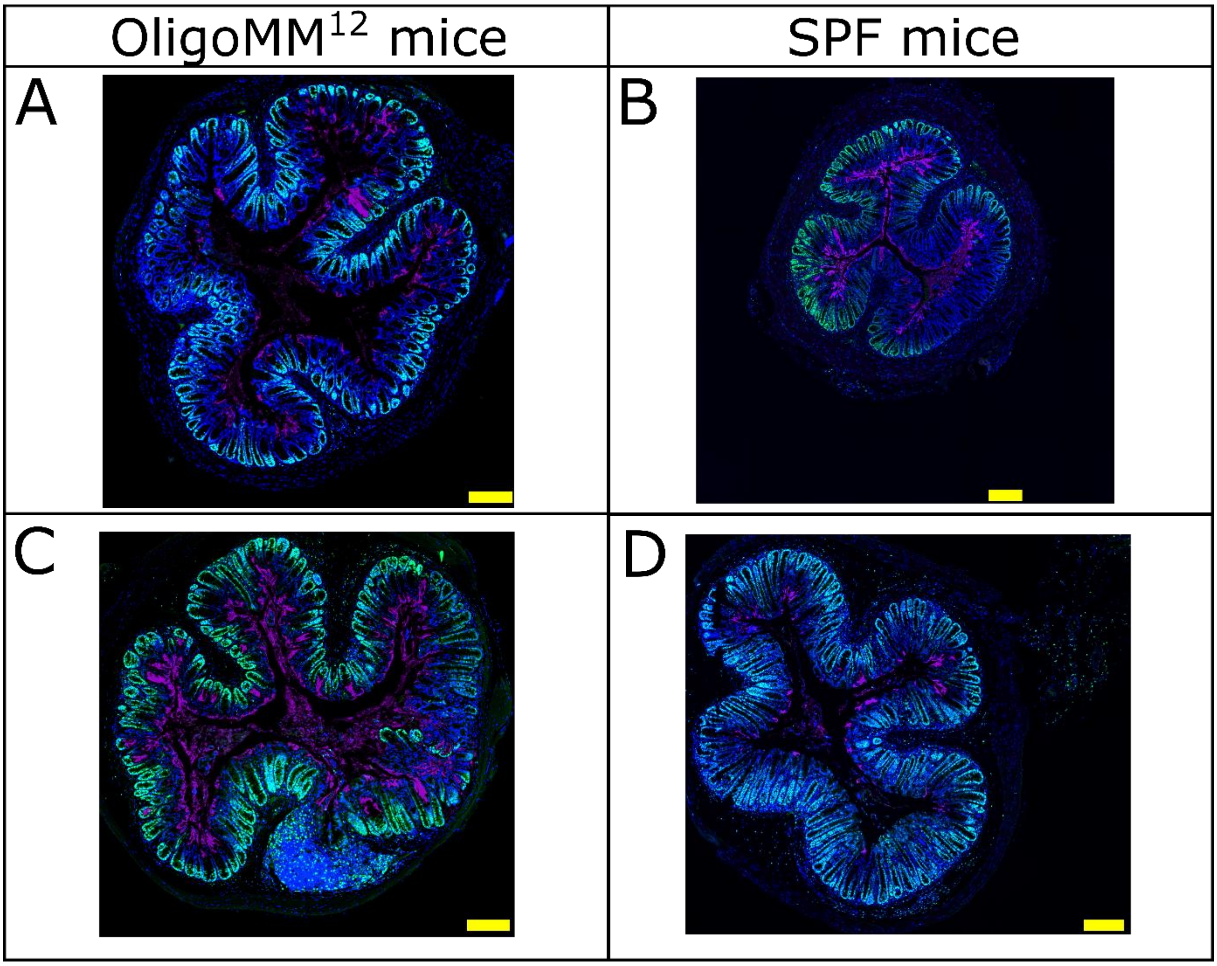

*Figure 4: Representative images of the colon in C. rodentium infected OligoMM[12] and SPF mice at endpoint (day 8). Immunofluorescence: C. rodentium (purple), proliferating cell nuclear antigen (green) and DAPI (blue). A) and C) are sections from OligoMM[12] mice and B) and D) are sections from SPF mice. Similar C. rodentium densities in the feces were measured for A) (1.49*10^9 CFU/g) and B) (2.61*10^9 CFU/g), as well as for C) (2.67*10^8 CFU/g) and D) (3.01*10^8 CFU/g). Yellow scale bars correspond to 200 µm.*

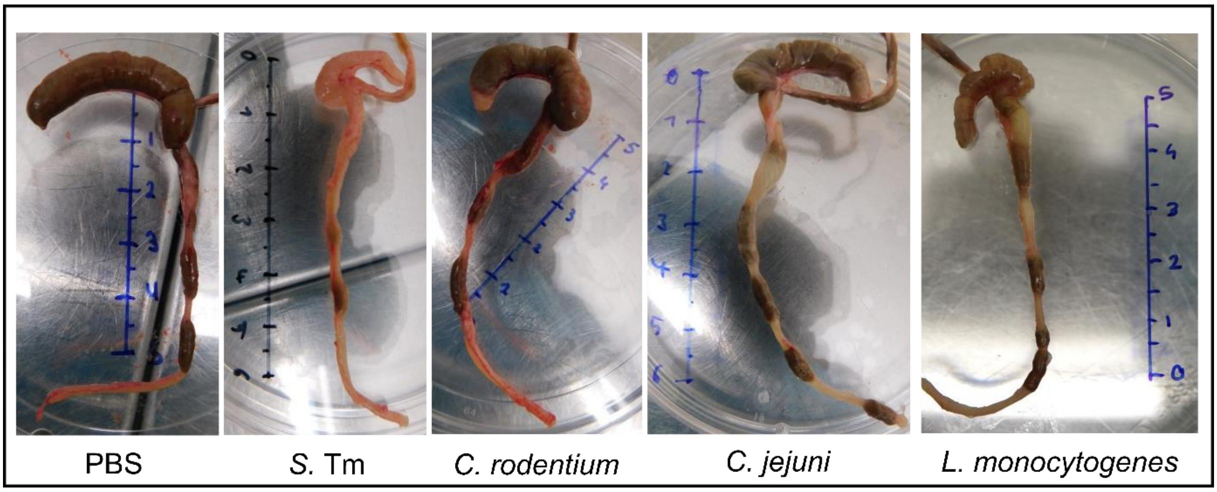

Figure 5: Representative macroscopic images of the cecum and colon of OligoMM^{12} mice infected with one of the four pathogens. The cecum and colon of a mock (PBS) treated mouse is shown for comparison. The unit of the blue scale bars is centimeter.

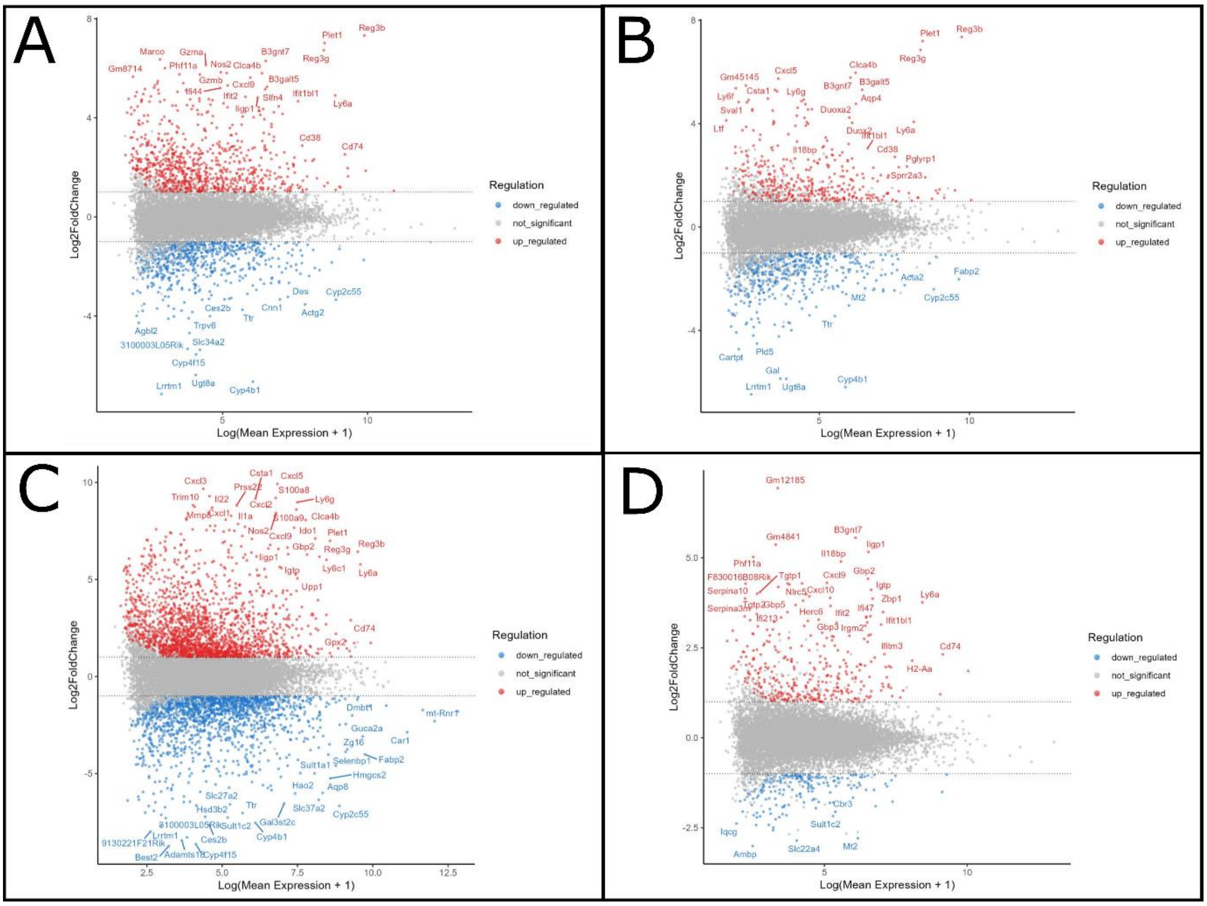

Figure 6: Differentially expressed genes in cecum tissue of OligoMM[12] mice. A) C. jejuni vs PBS control. B) C. rodentium vs PBS control. C) S. Tm vs PBS control. D) L. monocytogenes vs PBS control. (Analysis by Alithea Genomics)

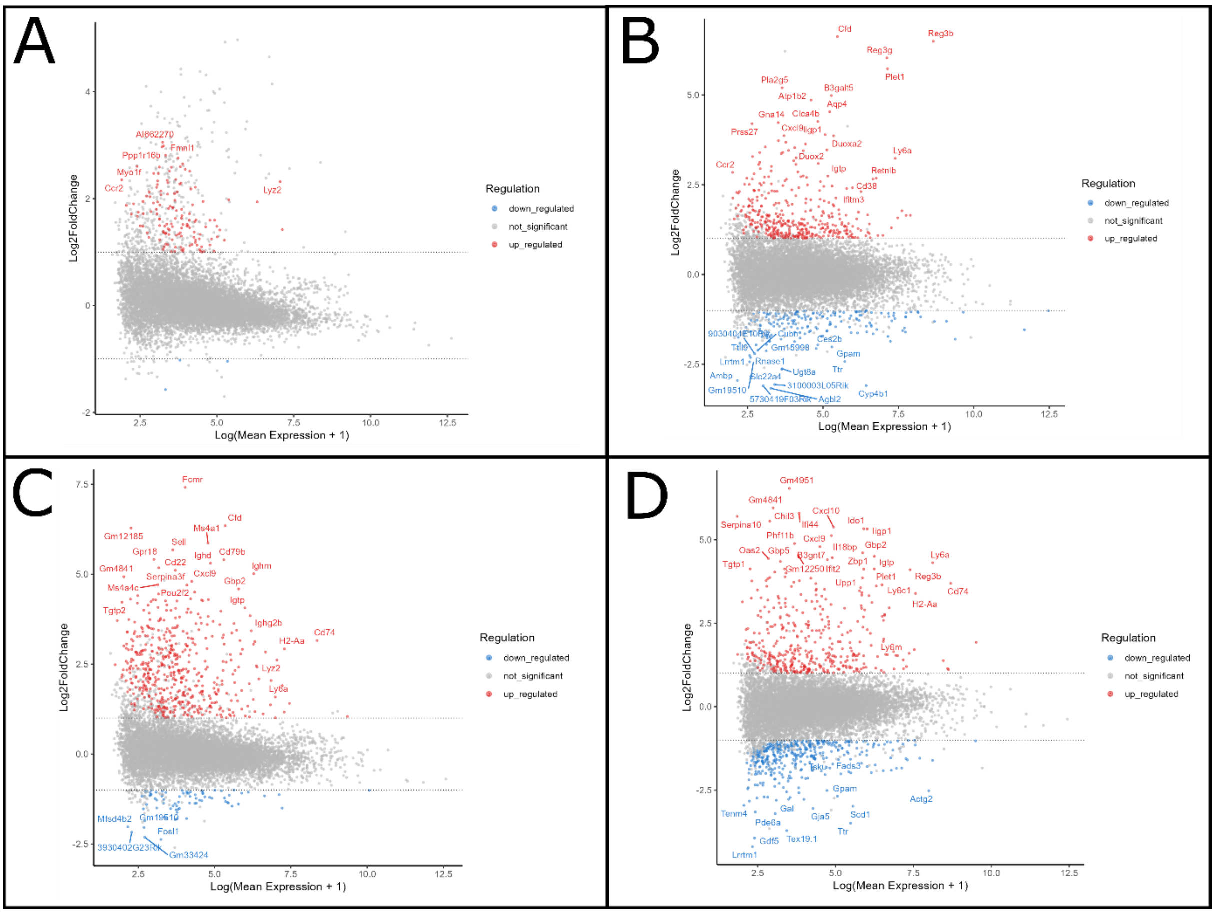

Figure 7: Differentially expressed genes in cecum tissue of SPF mice. A) C. jejuni vs PBS control. B) C. rodentium vs PBS control. C) S. Tm vs PBS control. D) L. monocytogenes vs PBS control. (Analysis by Alithea Genomics)

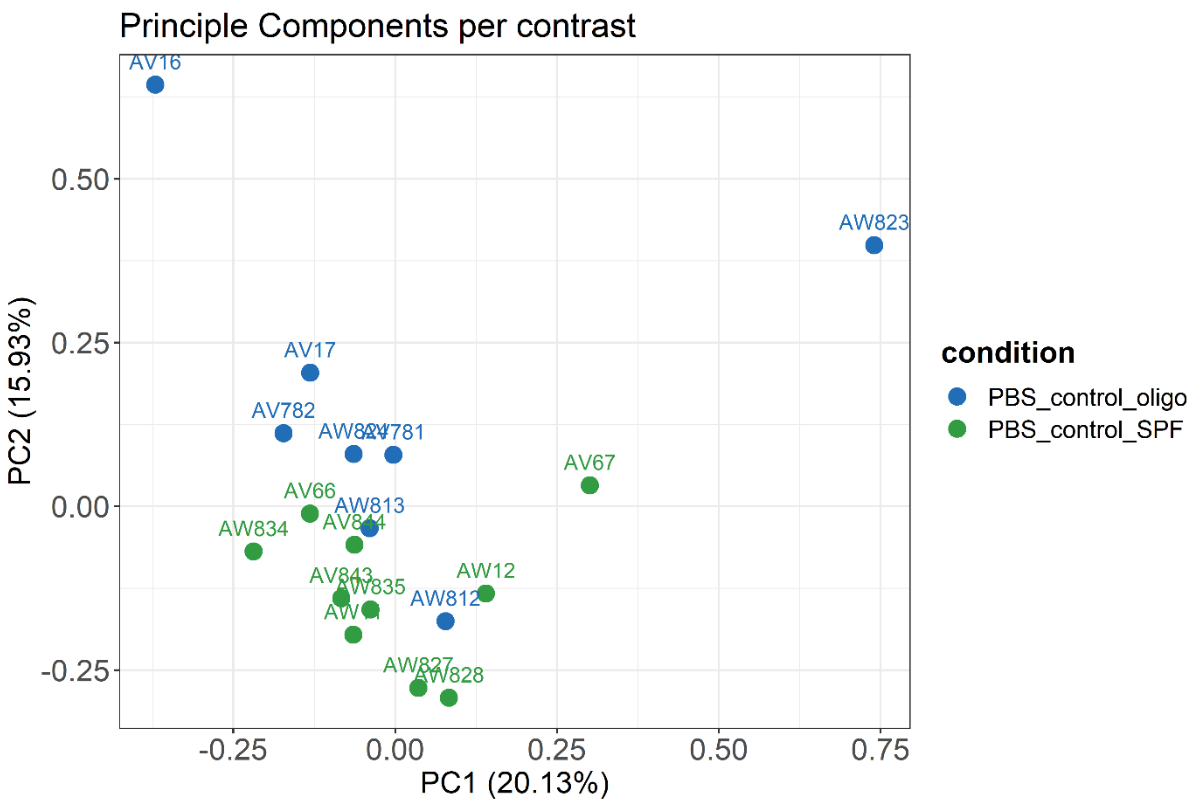

Figure 8: Principal component analysis of cecum tissue gene expression in mock (PBS) treated OligoMM[12] and SPF mice.

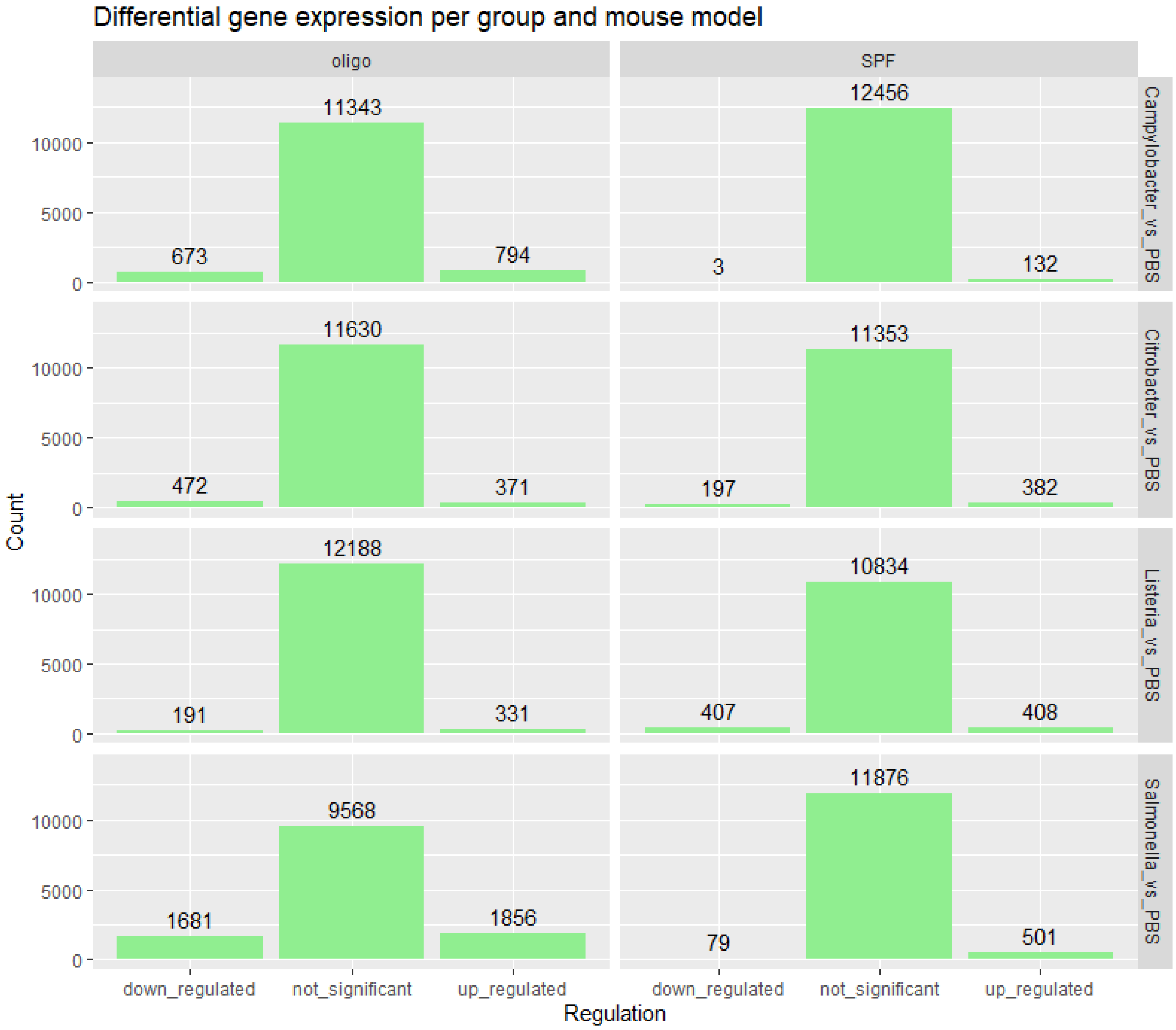

Figure 9: Number of detected genes in the cecum tissue of infected OligoMM[12] and SPF mice.

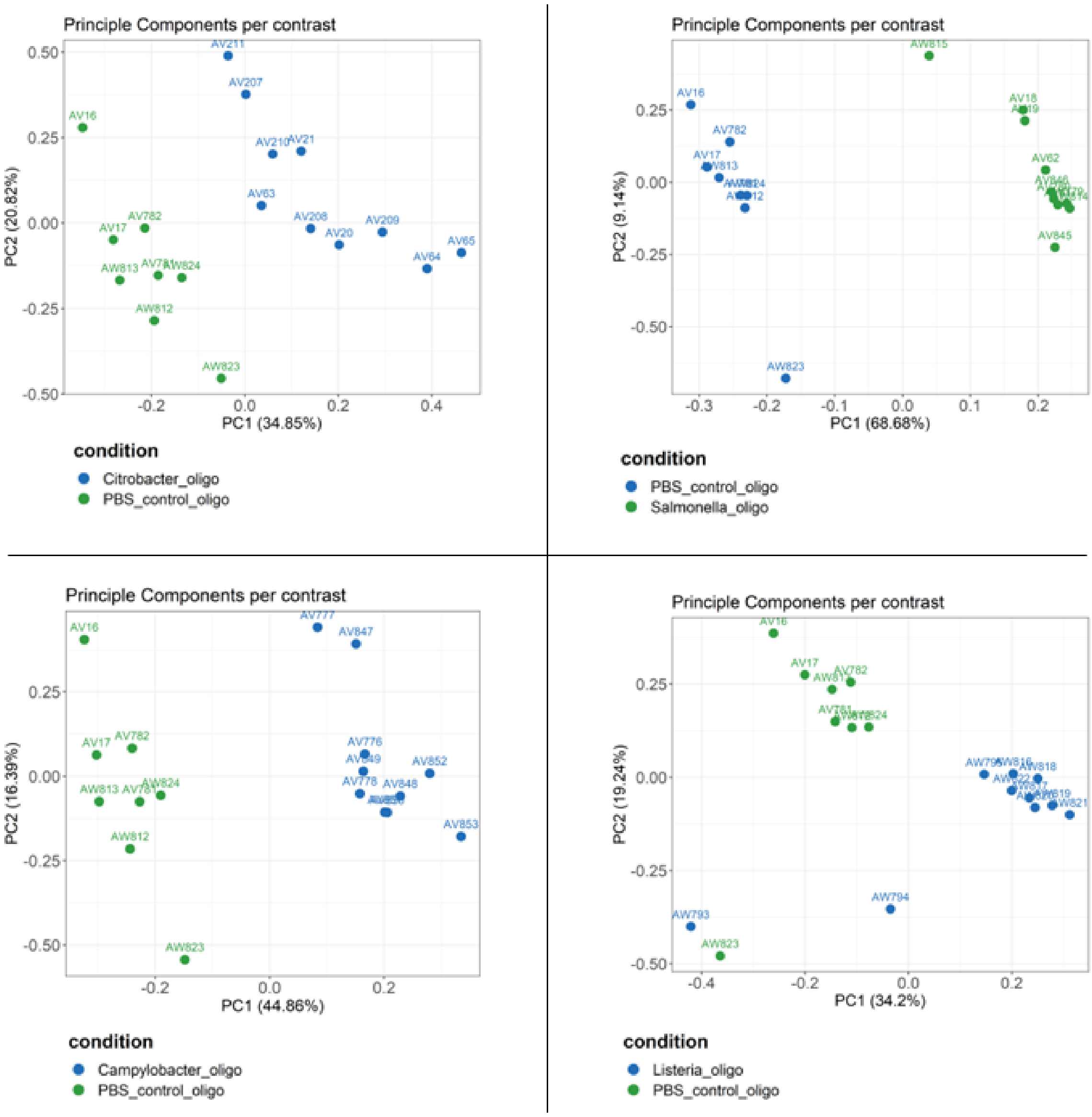

Figure 10: Principal component analysis of RNAseq data comparing infected animals with mock infected mice (all OligoMM[12]). (Analysis by Alithea Genomics)

Annotation Cluster 1	Enrichment Score: 24.29		Count	P_Value	Benjamini
GOTERM_BP_DIRECT	immune system process	RT	43	6.0E-34	7.2E-31
UP_KW_BIOLOGICAL_PROCESS	Immunity	RT	41	5.6E-26	3.1E-24
UP_KW_BIOLOGICAL_PROCESS	Innate immunity	RT	29	9.4E-21	2.6E-19
GOTERM_BP_DIRECT	innate immune response	RT	34	2.1E-19	1.3E-16
**Annotation Cluster 2**	**Enrichment Score: 9.07**		**Count**	**P_Value**	**Benjamini**
GOTERM_BP_DIRECT	cellular response to interferon-gamma	RT	15	1.5E-14	3.8E-12
GOTERM_BP_DIRECT	cellular response to lipopolysaccharide	RT	17	1.2E-10	1.6E-8
GOTERM_BP_DIRECT	defense response to bacterium	RT	16	5.8E-8	5.8E-6
KEGG_PATHWAY	NOD-like receptor signaling pathway	RT	12	5.0E-6	1.4E-4
**Annotation Cluster 3**	**Enrichment Score: 7.58**		**Count**	**P_Value**	**Benjamini**
GOTERM_BP_DIRECT	cellular response to interferon-beta	RT	13	1.6E-14	3.8E-12
GOTERM_BP_DIRECT	defense response	RT	15	1.6E-13	2.8E-11
GOTERM_BP_DIRECT	defense response to protozoan	RT	11	1.6E-13	2.8E-11
INTERPRO	Interferon-inducible GTPase	RT	7	9.3E-10	2.7E-7
UP_SEQ_FEATURE	DOMAIN:IRG-type G	RT	7	1.0E-9	5.1E-7
GOTERM_MF_DIRECT	GTP binding	RT	17	1.1E-8	1.8E-6
UP_SEQ_FEATURE	DOMAIN:GB1/RHD3-type G	RT	6	2.8E-8	7.2E-6
INTERPRO	Guanylate-binding protein, N-terminal	RT	6	3.7E-8	5.4E-6
GOTERM_CC_DIRECT	symbiont-containing vacuole membrane	RT	5	1.9E-7	1.8E-5
GOTERM_MF_DIRECT	GTPase activity	RT	14	3.9E-7	4.3E-5
UP_KW_LIGAND	GTP-binding	RT	13	3.4E-5	6.7E-4
GOTERM_CC_DIRECT	cytoplasmic vesicle	RT	15	1.8E-3	2.2E-2
GOTERM_CC_DIRECT	endoplasmic reticulum membrane	RT	16	1.8E-3	2.2E-2
INTERPRO	P-loop containing nucleoside triphosphate hydrolase	RT	15	2.0E-3	6.7E-2

Figure 11: DAVID analysis with the genes (139) that are upregulated in the cecum tissue of OligoMM[12] mice in all four pathogen groups. The list of Ensemble gene IDs were submitted to the DAVID online platform (default annotation categories) and "Functional Annotation Clustering" was chosen to obtain the results shown in this figure.

Annotation Cluster 1	Enrichment Score: 7.96		Count	P_Value	Benjamini
UP_KW_BIOLOGICAL_PROCESS	Immunity	RT	18	3.0E-12	8.1E-11
GOTERM_BP_DIRECT	immune system process	RT	17	4.5E-11	2.2E-8
UP_KW_BIOLOGICAL_PROCESS	Adaptive immunity	RT	11	9.7E-9	1.3E-7
GOTERM_BP_DIRECT	adaptive immune response	RT	10	9.0E-7	1.3E-4
GOTERM_BP_DIRECT	innate immune response	RT	14	1.1E-6	1.3E-4
UP_KW_BIOLOGICAL_PROCESS	Innate immunity	RT	10	1.4E-6	1.3E-5
**Annotation Cluster 2**	**Enrichment Score: 2.51**		**Count**	**P_Value**	**Benjamini**
GOTERM_CC_DIRECT	external side of plasma membrane	RT	16	4.6E-8	4.9E-6
UP_SEQ_FEATURE	TOPO_DOM:Extracellular	RT	22	9.0E-5	2.0E-2
UP_SEQ_FEATURE	TOPO_DOM:Cytoplasmic	RT	26	1.3E-4	2.0E-2
GOTERM_CC_DIRECT	cell surface	RT	11	6.1E-4	1.3E-2
GOTERM_CC_DIRECT	plasma membrane	RT	33	8.1E-3	1.2E-1
UP_KW_MOLECULAR_FUNCTION	Receptor	RT	17	1.2E-2	3.4E-1
GOTERM_CC_DIRECT	membrane	RT	38	2.4E-2	2.9E-1
UP_KW_CELLULAR_COMPONENT	Membrane	RT	43	3.5E-2	6.7E-1
UP_SEQ_FEATURE	TRANSMEM:Helical	RT	32	4.9E-2	1.0E0
UP_KW_DOMAIN	Transmembrane helix	RT	33	5.2E-2	3.6E-1
GOTERM_CC_DIRECT	integral component of membrane	RT	30	8.2E-2	7.3E-1
UP_KW_DOMAIN	Transmembrane	RT	33	1.4E-1	3.6E-1
**Annotation Cluster 3**	**Enrichment Score: 2.49**		**Count**	**P_Value**	**Benjamini**
KEGG_PATHWAY	T cell receptor signaling pathway	RT	6	1.1E-4	1.1E-2
BIOCARTA	The Co-Stimulatory Signal During T-cell Activation	RT	4	1.2E-4	2.5E-3
KEGG_PATHWAY	Th17 cell differentiation	RT	5	7.3E-4	2.7E-2
BIOCARTA	Lck and Fyn tyrosine kinases in initiation of TCR Activation	RT	3	1.5E-3	1.2E-2
GOTERM_BP_DIRECT	cell surface receptor signaling pathway	RT	5	6.7E-3	1.6E-1
BIOCARTA	Activation of Csk by cAMP-dependent Protein Kinase Inhibits Signaling through the T Cell Receptor	RT	3	6.7E-3	1.8E-2
BIOCARTA	T Cell Receptor Signaling Pathway	RT	3	2.8E-2	6.2E-2
KEGG_PATHWAY	Th1 and Th2 cell differentiation	RT	3	4.7E-2	5.2E-1
KEGG_PATHWAY	PD-L1 expression and PD-1 checkpoint pathway in cancer	RT	3	4.7E-2	5.2E-1

Figure 12: DAVID analysis with the genes (88) that are uniquely upregulated in the cecum tissue of OligoMM^{12} mice infected with C. jejuni. The list of Ensemble gene IDs was submitted to the DAVID online platform (default annotation categories) and "Functional Annotation Clustering" was chosen to obtain the results shown in this figure.

Annotation Cluster 1	Enrichment Score: 2.28		Count	P_Value	Benjamini
GOTERM_MF_DIRECT	hydrolase activity	RT	8	3.2E-4	2.5E-2
UP_KW_MOLECULAR_FUNCTION	Hydrolase	RT	8	1.9E-3	4.0E-2
UP_SEQ_FEATURE	PROPEP:Activation peptide	RT	3	2.0E-3	2.6E-1
GOTERM_MF_DIRECT	peptidase activity	RT	4	1.1E-2	4.2E-1
GOTERM_BP_DIRECT	proteolysis	RT	4	1.3E-2	1.0E0
UP_KW_PTM	Zymogen	RT	3	2.3E-2	2.6E-1
UP_KW_MOLECULAR_FUNCTION	Protease	RT	4	2.4E-2	2.6E-1
**Annotation Cluster 2**	**Enrichment Score: 1.55**		**Count**	**P_Value**	**Benjamini**
GOTERM_CC_DIRECT	extracellular space	RT	7	3.7E-3	2.5E-1
UP_KW_DOMAIN	Signal	RT	8	3.0E-2	2.1E-1
UP_KW_CELLULAR_COMPONENT	Secreted	RT	4	2.0E-1	1.0E0
**Annotation Cluster 3**	**Enrichment Score: 0.88**		**Count**	**P_Value**	**Benjamini**
GOTERM_BP_DIRECT	response to bacterium	RT	3	2.5E-2	1.0E0
UP_KW_CELLULAR_COMPONENT	Secreted	RT	4	2.0E-1	1.0E0
GOTERM_CC_DIRECT	extracellular region	RT	3	4.5E-1	1.0E0

Figure 13: DAVID analysis with the genes (20) that are uniquely upregulated in the cecum tissue of OligoMM^{12} mice infected with C. rodentium. The list of Ensemble gene IDs was submitted to the DAVID online platform (default annotation categories) and "Functional Annotation Clustering" was chosen to obtain the results shown in this figure.

Annotation Cluster 1	Enrichment Score: 0.19	G		Count	P_Value	Benjamini
UP_KW_PTM	Disulfide bond	RT		3	5.7E-1	1.0E0
UP_SEQ_FEATURE	CARBOHYD:N-linked (GlcNAc...) asparagine	RT		3	6.7E-1	1.0E0
UP_KW_PTM	Glycoprotein	RT		3	7.1E-1	1.0E0
**Annotation Cluster 2**	**Enrichment Score: 0.15**	G		**Count**	**P_Value**	**Benjamini**
UP_KW_DOMAIN	Transmembrane helix	RT		4	5.4E-1	1.0E0
UP_KW_DOMAIN	Transmembrane	RT		4	6.0E-1	1.0E0
GOTERM_CC_DIRECT	membrane	RT		5	7.0E-1	1.0E0
GOTERM_CC_DIRECT	plasma membrane	RT		4	7.1E-1	1.0E0
GOTERM_CC_DIRECT	integral component of membrane	RT		4	7.5E-1	1.0E0
UP_SEQ_FEATURE	TRANSMEM:Helical	RT		4	8.0E-1	1.0E0
UP_KW_CELLULAR_COMPONENT	Membrane	RT		4	9.7E-1	1.0E0

Figure 14: DAVID analysis with the genes (16) that are uniquely upregulated in the cecum tissue of OligoMM[12] mice infected with L. monocytogenes. The list of Ensemble gene IDs was submitted to the DAVID online platform (default annotation categories) and "Functional Annotation Clustering" was chosen to obtain the results shown in this figure.

Annotation Cluster 1	Enrichment Score: 9.94	G		Count	P_Value	Benjamini
GOTERM_BP_DIRECT	cell division	RT		56	8.3E-12	2.0E-8
UP_KW_BIOLOGICAL_PROCESS	Cell division	RT		57	3.8E-11	5.0E-9
UP_KW_BIOLOGICAL_PROCESS	Mitosis	RT		46	7.3E-11	5.0E-9
GOTERM_BP_DIRECT	cell cycle	RT		72	6.2E-10	5.1E-7
UP_KW_BIOLOGICAL_PROCESS	Cell cycle	RT		75	1.4E-9	6.6E-8
**Annotation Cluster 2**	**Enrichment Score: 9.17**	G		**Count**	**P_Value**	**Benjamini**
GOTERM_CC_DIRECT	extracellular region	RT		164	2.3E-13	1.7E-10
GOTERM_CC_DIRECT	extracellular space	RT		165	5.5E-11	1.0E-8
UP_KW_CELLULAR_COMPONENT	Secreted	RT		155	4.1E-10	2.1E-8
UP_KW_DOMAIN	Signal	RT		290	3.9E-5	1.2E-3
**Annotation Cluster 3**	**Enrichment Score: 7.05**	G		**Count**	**P_Value**	**Benjamini**
UP_SEQ_FEATURE	CARBOHYD:N-linked (GlcNAc...) asparagine	RT		291	4.3E-20	1.2E-16
UP_KW_DOMAIN	Signal	RT		290	3.9E-5	1.2E-3
UP_KW_PTM	Glycoprotein	RT		319	2.7E-3	3.8E-2
UP_KW_PTM	Disulfide bond	RT		253	1.4E-2	1.4E-1

Figure 15: DAVID analysis with the genes (1132) that are uniquely upregulated in the cecum tissue of OligoMM[12] mice infected with S. Tm. The list of Ensemble gene IDs was submitted to the DAVID online platform (default annotation categories) and "Functional Annotation Clustering" was chosen to obtain the results shown in this figure.

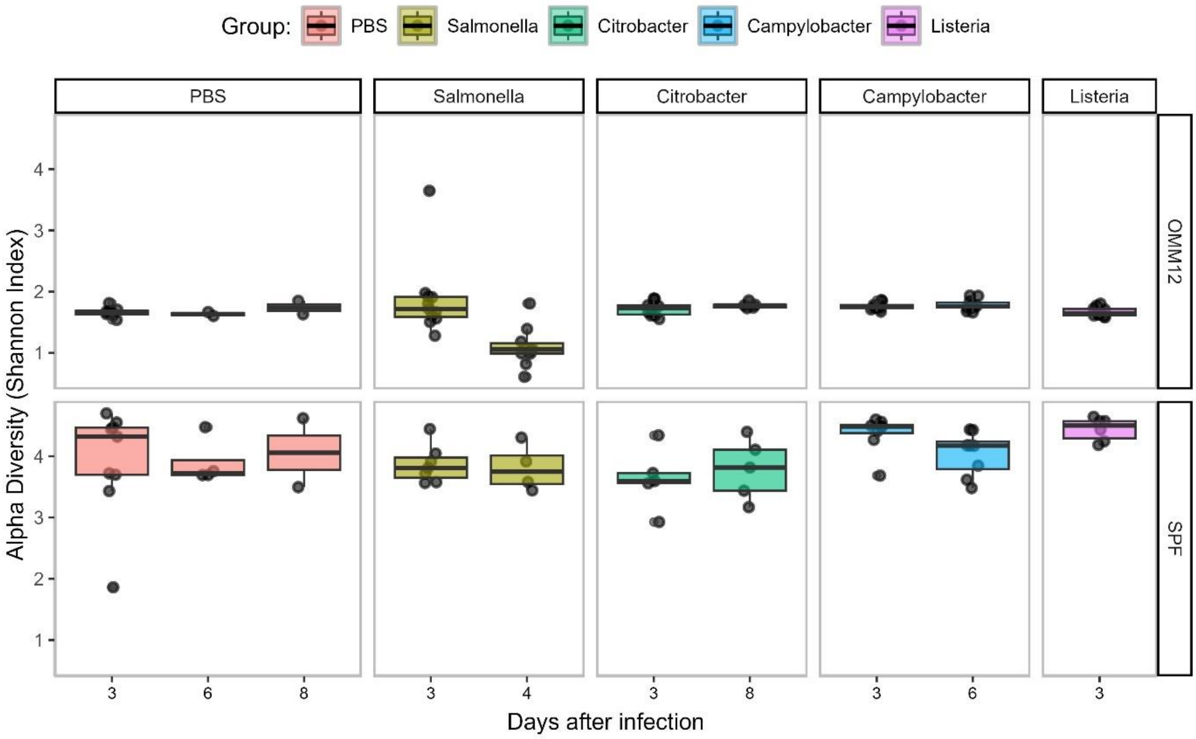

Figure 16: Alpha diversity of taxa in the feces of infected or mock (PBS) treated OligoMM[12] and SPF mice.

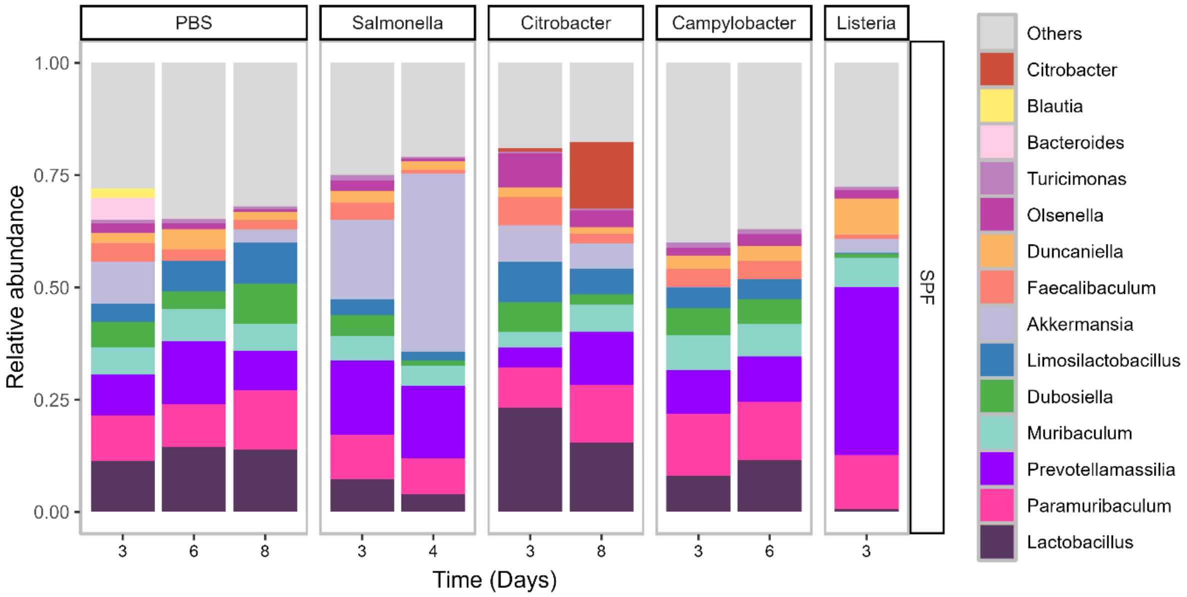

Figure 17: Relative abundance of genera in the SPF mice infected with one of the four pathogens or mock (PBS) treated.

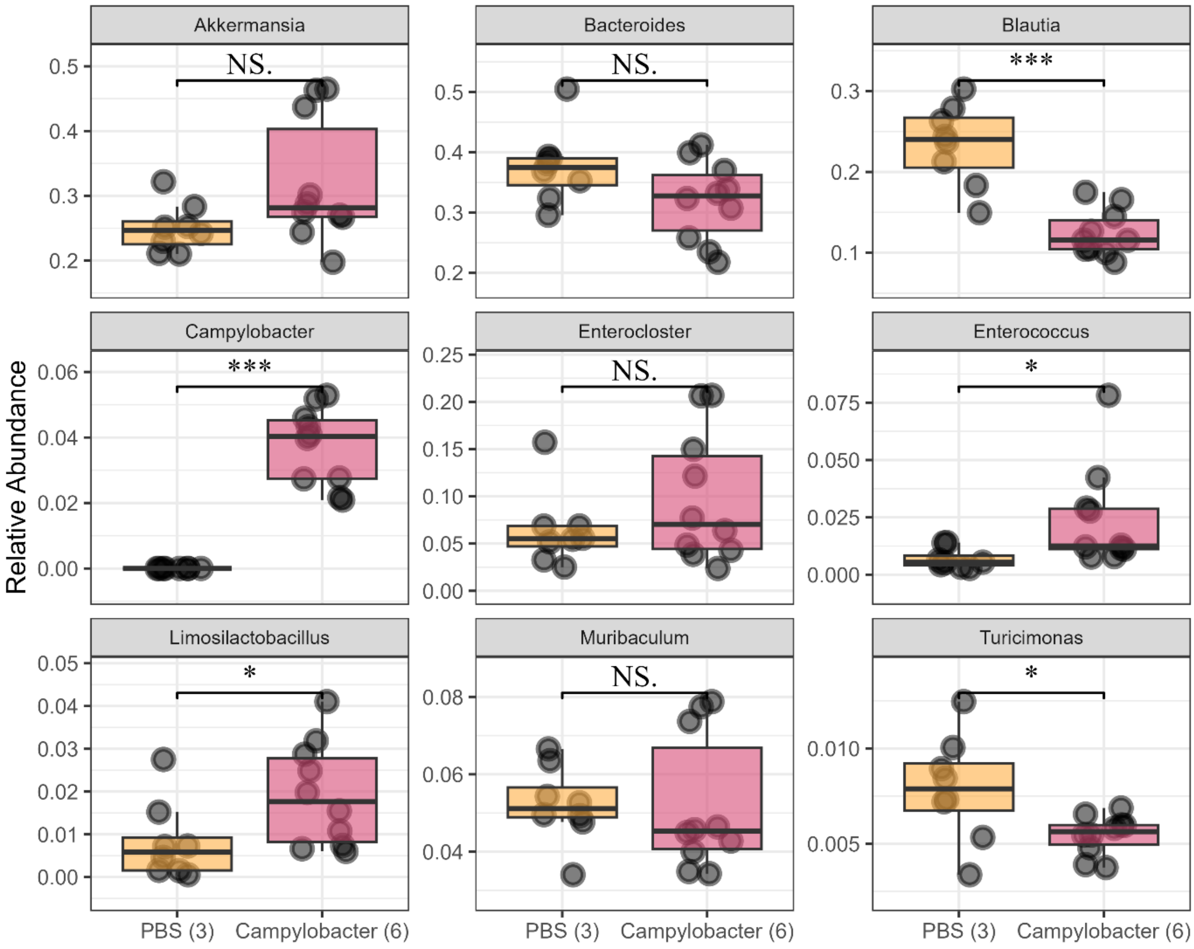

Figure 18: Predominant genera in C. jejuni infected OligoMM[12] mice

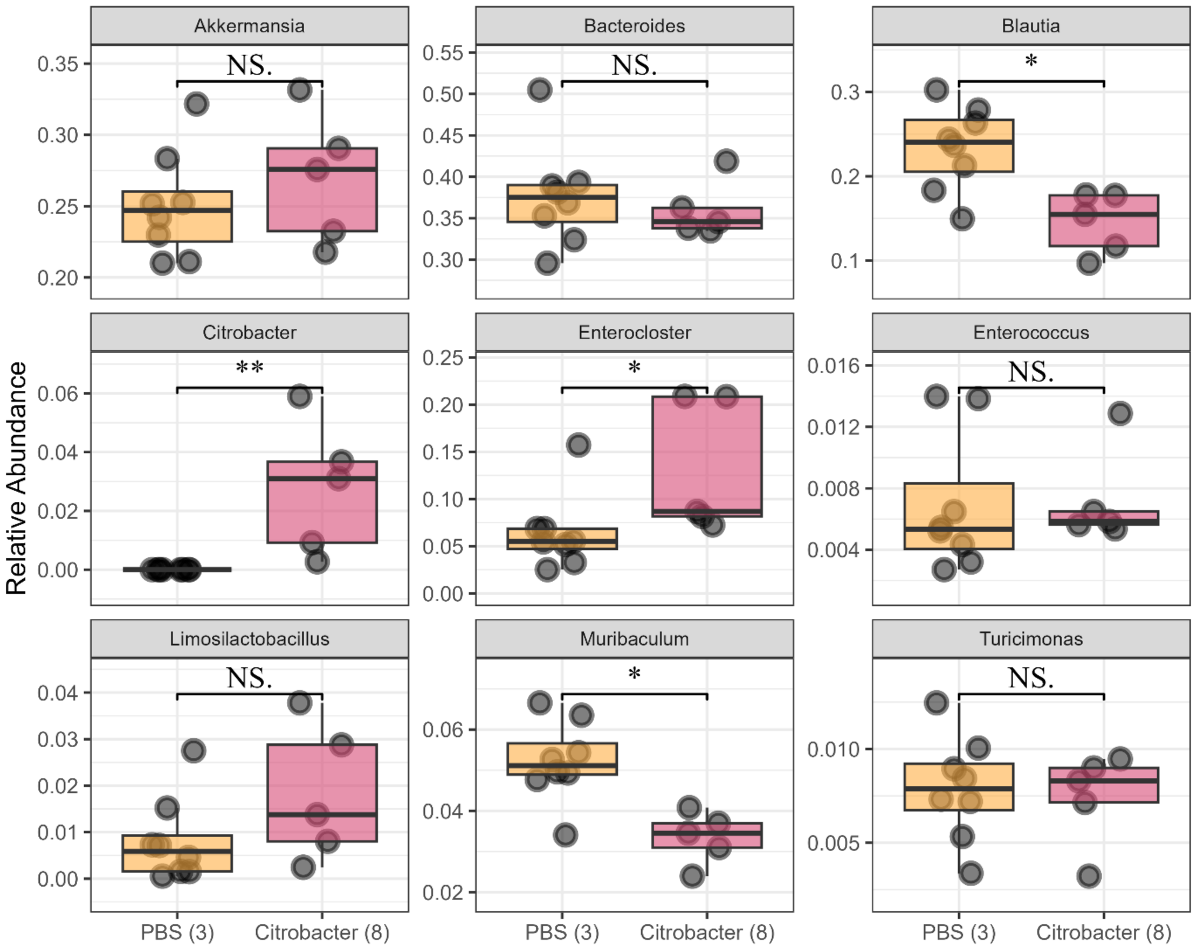

Figure 19: Predominant general in C. rodentium infected OligoMM[12] mice.

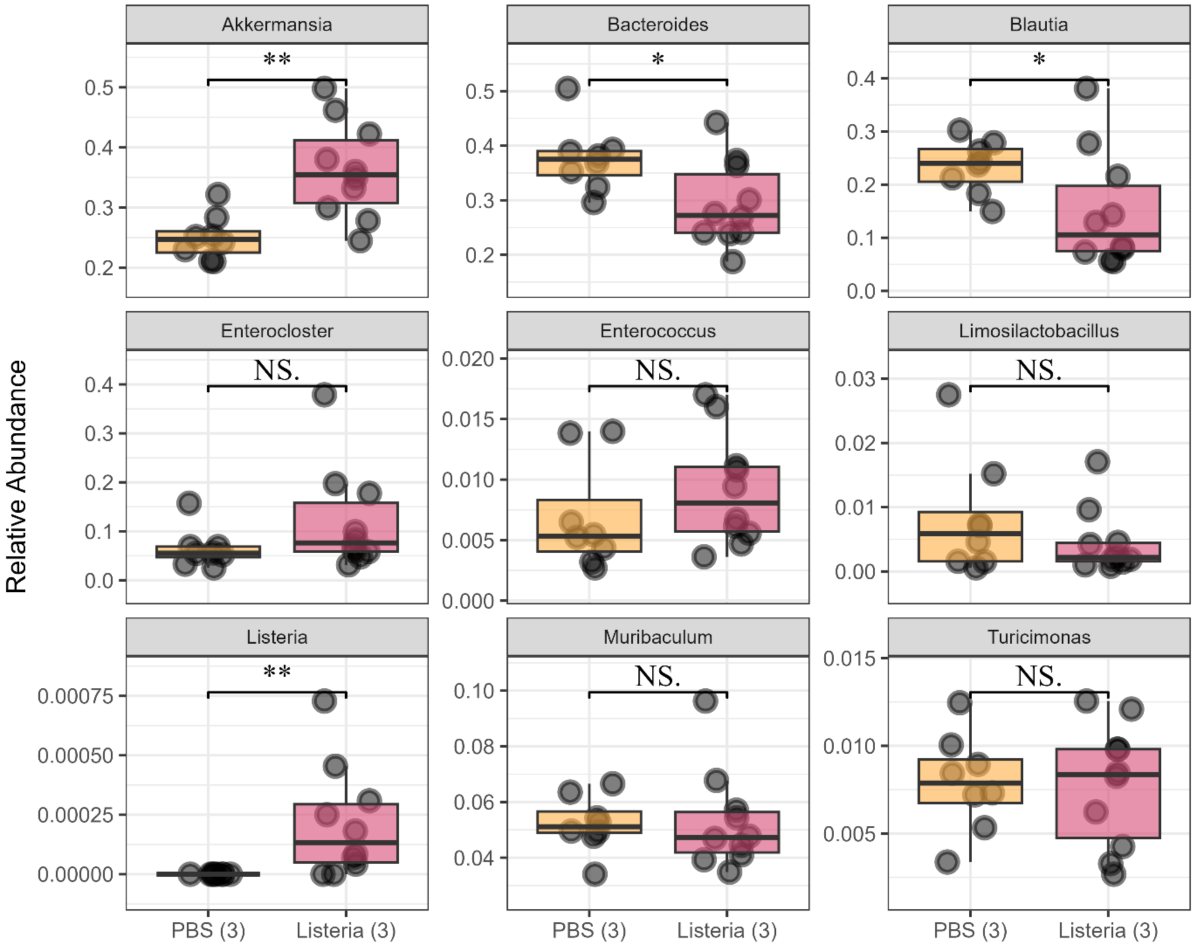

Figure 20: Predominant genera in L. monocytogenes infected OligoMM[12] mice.

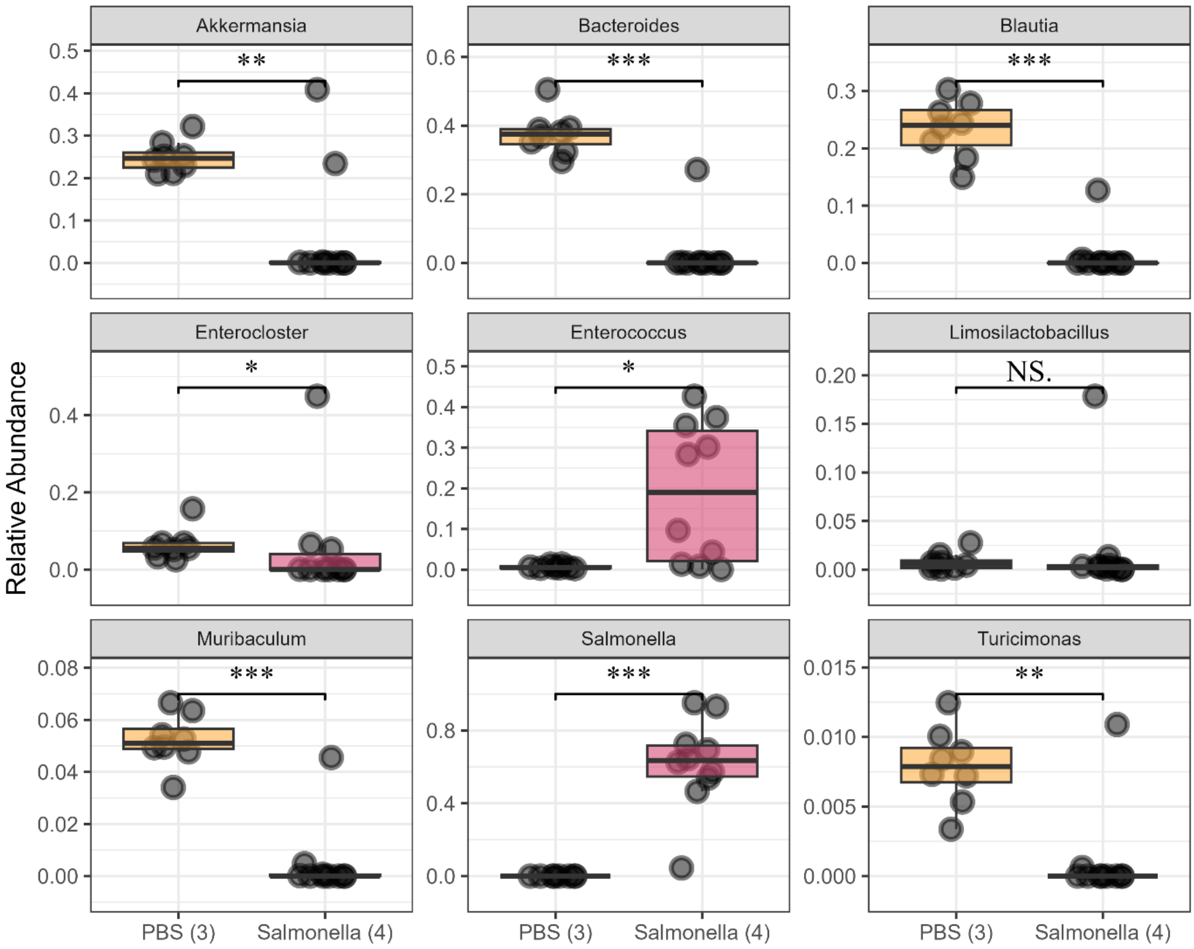

Figure 21: Predominant genera in S. Tm infected OligoMM^{12} mice.

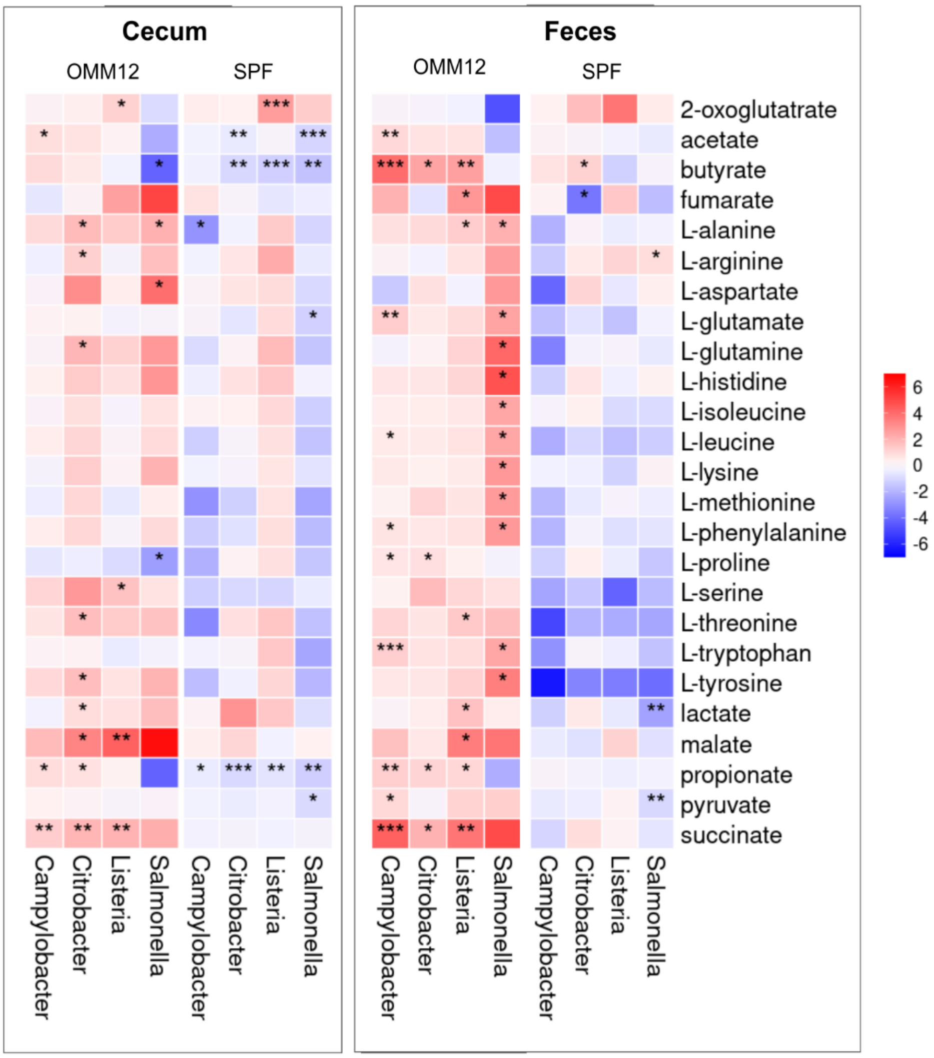

*Figure 22: Heatmap of metabolite abundance in the feces and cecum of OligoMM[12] and SPF mice relative to mock (PBS) treated mice. Color gradient indicates log2 fold change of average peak area of metabolites relative to PBS, and the stars indicate significance. (Wilcoxon test: $p < 0.05$ = *, $p < 0.01$ = **, $p < 0.001$ = ***, absence denotes non-significant values)*

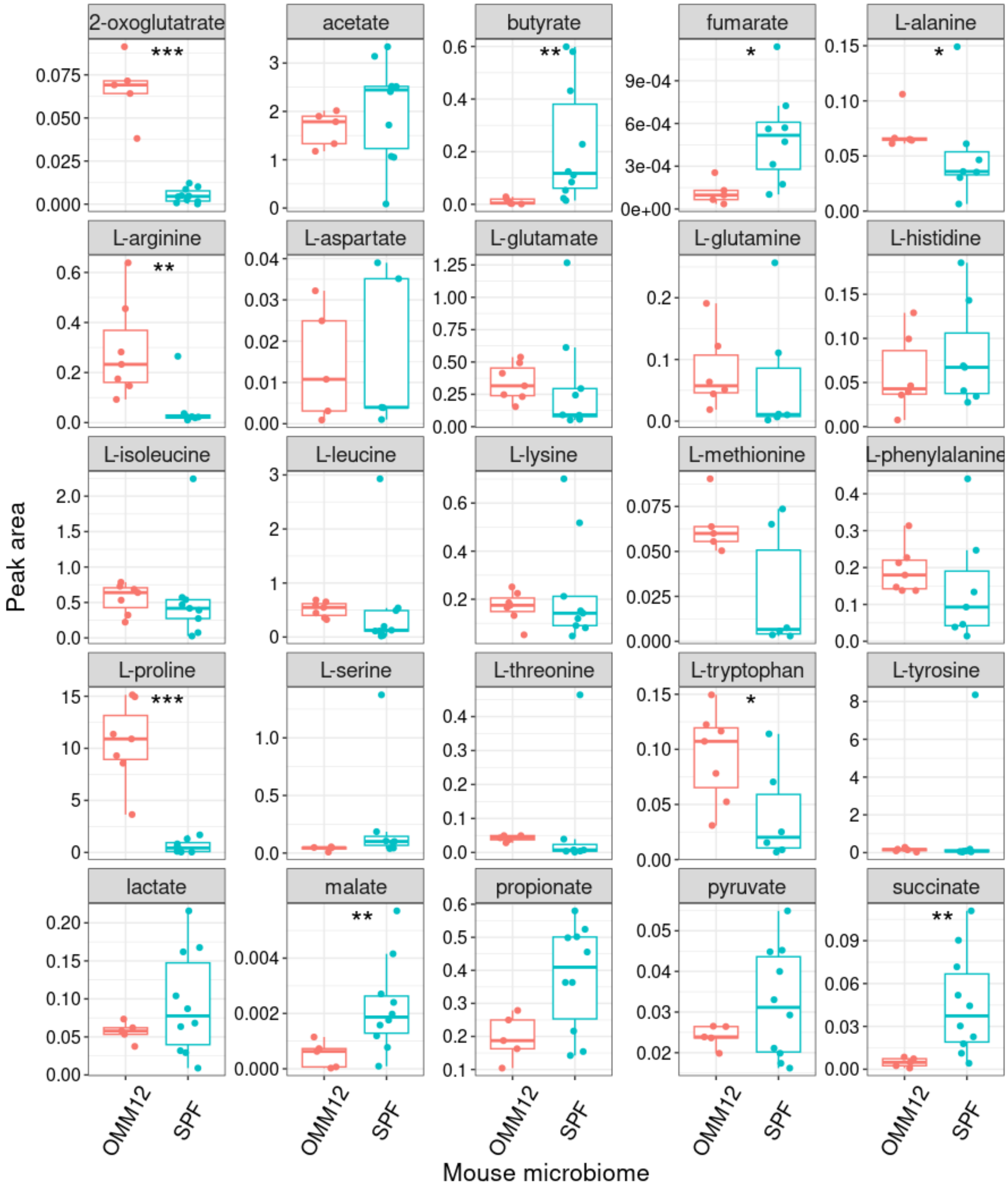

*Figure 23: Targeted metabolomics data of feces of the mock (PBS) treated OligoMM[12] and SPF mice (Wilcoxon test: p < 0.05 = *, p < 0.01 = **, p < 0.001 = ***, absence denotes non-significant values)*

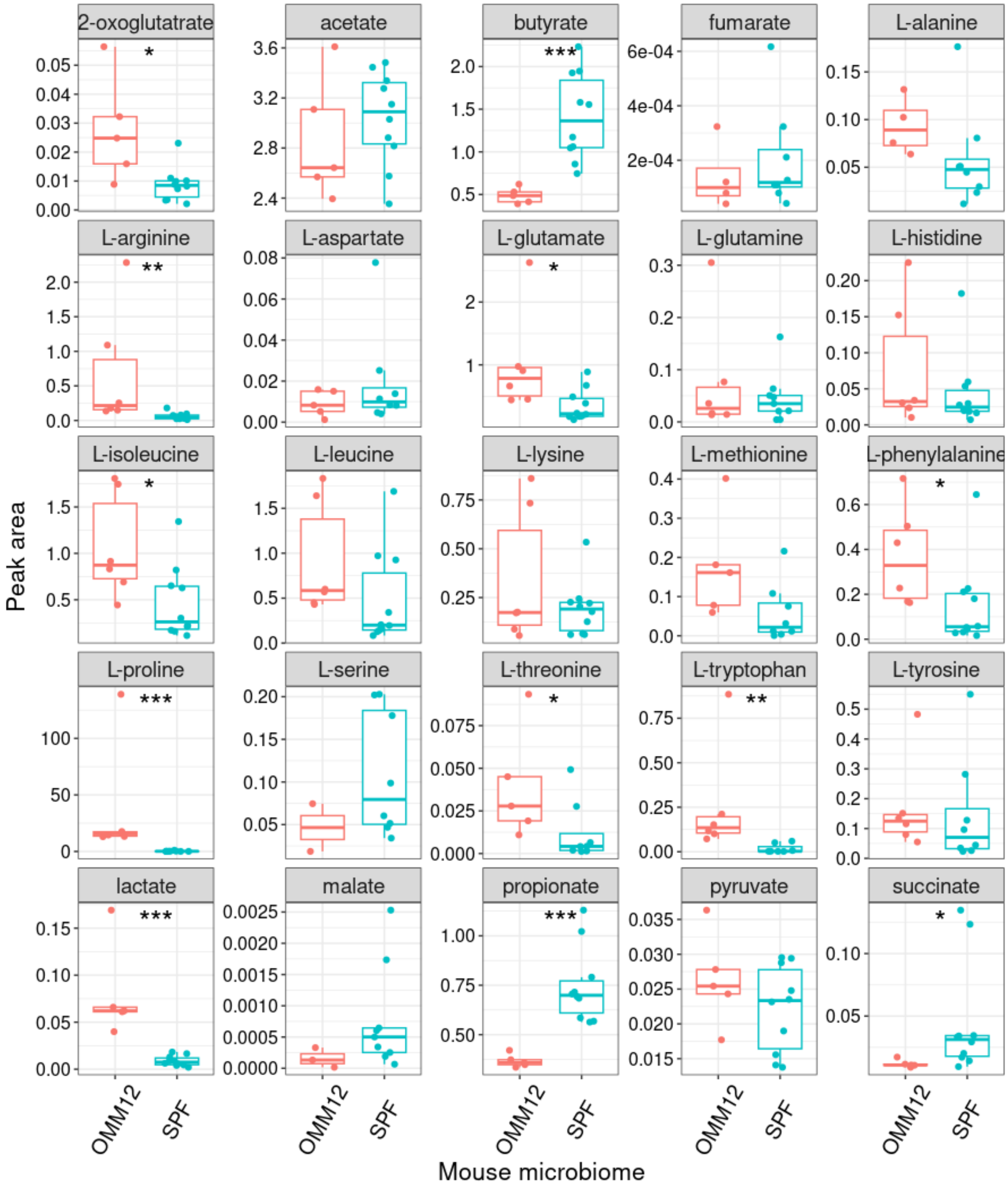

*Figure 24: Targeted metabolomics data of the mock (PBS) treated OligoMM[12] and SPF mice in cecum (Wilcoxon test: $p < 0.05$ = *, $p < 0.01$ = **, $p < 0.001$ = ***, absence denotes non-significant values)*

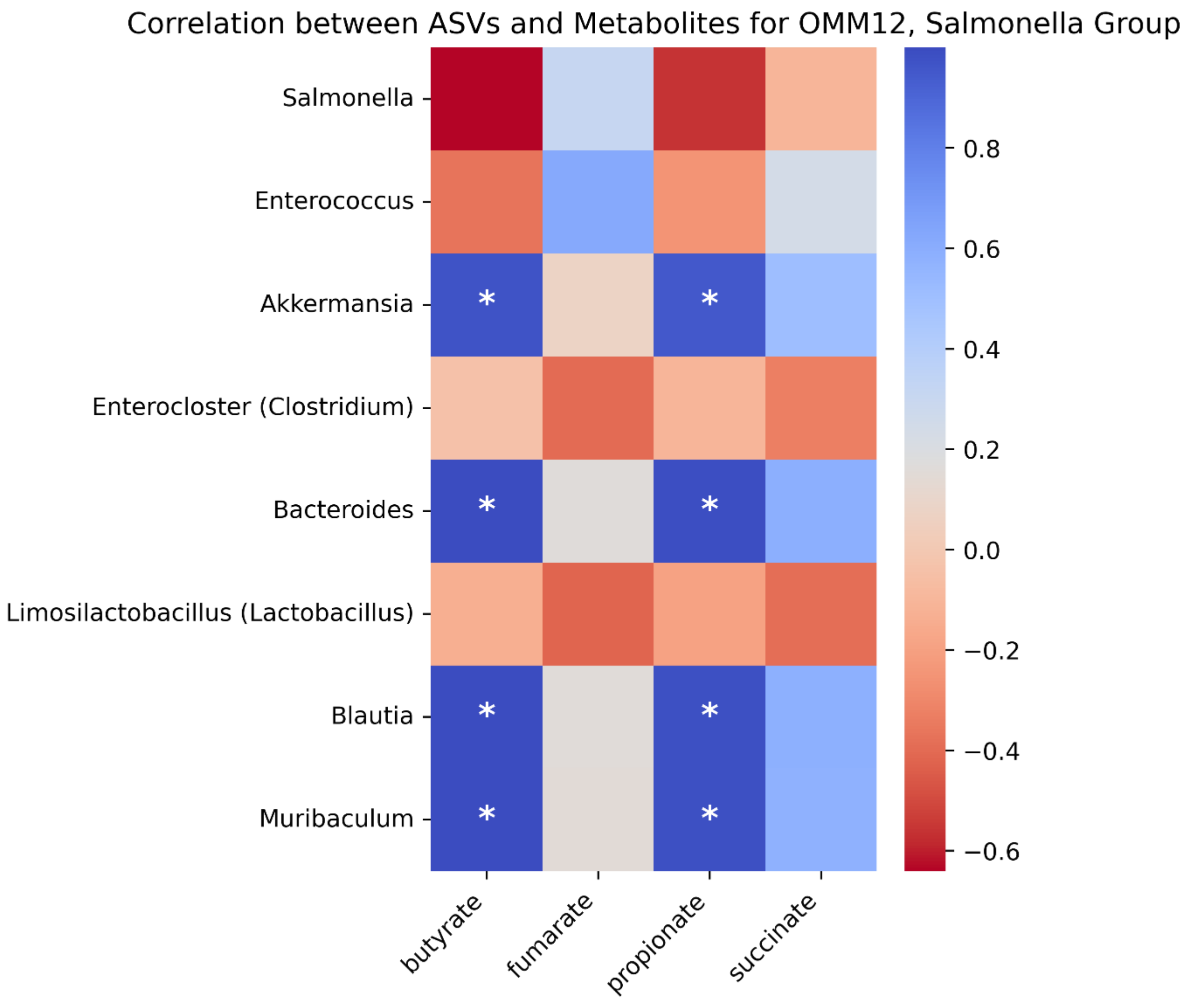

Figure 25: Correlation of the abundance of genera and selected metabolites in S. Tm infected OligoMM[12] mice.

Table 1: Top20 up regulated genes in OligoMM^{12} mice infected with the four pathogens.

C. jejuni		***C. rodentium***		***L. monocytogenes***		***S.* Tm**	
Gene name	Log2 fold change	Gene name	Log2 fold change	Gene name	Log2 fold change	Gene name	Log2 fold change
Reg3b	7.32	Reg3b	7.35	Gm12185	6.94	Cxcl5	9.92
Plet1	7.02	Plet1	7.19	B3gnt7	5.56	Cxcl3	9.67
Reg3g	6.73	Reg3g	6.85	Gm4841	5.37	Il22	9.29
Marco	6.35	Clca4b	5.96	Iigp1	5.16	S100a8	9.20
B3gnt7	6.29	B3gnt7	5.78	Phf11a	5.02	Csta1	9.16
Gzma	6.13	Cxcl5	5.74	Il18bp	4.90	Ly6g	8.97
Phf11a	6.00	Gm45145	5.47	Gbp2	4.42	Trim10	8.83
Gzmb	5.84	Ly6f	5.37	Cxcl9	4.30	Prss22	8.82
Nos2	5.80	B3galt5	5.31	Gm12250	4.29	Cxcl2	8.74
Clca4b	5.79	Csta1	5.30	Art2a	4.29	Mmp8	8.74
Gm4841	5.75	Prss27	5.25	F830016B08Rik	4.28	Cxcl1	8.70
Gm4951	5.75	Gm14137	4.97	Gm4951	4.26	S100a9	8.60
Gm8714	5.66	Ly6g	4.93	Serpina3f	4.19	Nos2	8.42
Cxcl9	5.62	Plet1os	4.91	Igtp	4.11	Il1a	8.28
Gm45145	5.48	Nos2	4.87	Tgtp1	4.05	Ifng	8.14
Gm14137	5.40	Sval1	4.83	Tgtp2	4.01	Clec4e	8.08
Lrrc4	5.38	Cxcl1	4.82	Phf11b	3.99	Ptx3	8.07
Plet1os	5.31	Aqp4	4.76	Ifi44	3.93	Clca4b	8.06
Ly6g	5.30	Cxcl2	4.75	Cxcl10	3.89	Ly6i	7.86
B3galt5	5.25	Atp1b2	4.61	Serpina10	3.87	Gm4841	7.77

Table 2: Top20 down regulated genes in OligoMM^{12} mice infected with one of the four pathogens.

C. jejuni		***C. rodentium***		***L. monocytogenes***		***S.* Tm**	
Gene name	Log2 fold change	Gene name	Log2 fold change	Gene name	Log2 fold change	Gene name	Log2 fold change
Lrrtm1	-7.15	Lrrtm1	-6.47	Ambp	-3.01	Best2	-8.74
Cyp4b1	-6.65	Cyp4b1	-6.19	Slc22a4	-2.86	Cyp4f15	-8.64
Ugt8a	-6.38	Ugt8a	-5.87	Mt2	-2.79	Adamts18	-8.44
Cyp4f15	-5.55	Gal	-5.86	Nmnat2	-2.57	3100003L05Rik	-8.29
Slc34a2	-5.37	Cartpt	-4.72	Pld5	-2.50	9130221F21Rik	-7.99
3100003L05Rik	-5.32	Pld5	-4.51	Gm33424	-2.43	Lrrtm1	-7.68
Trpv6	-4.69	3100003L05Rik	-4.23	Lrrtm1	-2.42	Ces2b	-7.64
Agbl2	-4.27	Ttll9	-4.20	Iqcg	-2.38	Cyp4b1	-7.54
Pld5	-4.11	Ambp	-4.07	Asb4	-2.27	Pld5	-7.29
Gal	-4.09	Slc34a2	-3.99	5430427M07Rik	-2.21	Sult1c2	-7.27
Krt17	-4.02	Col8a2	-3.87	Best2	-2.21	Hsd3b2	-7.22
Ces2b	-4.01	Slc22a4	-3.85	Gm15972	-2.18	Ugt2b36	-7.12
Gm45004	-3.99	Pde6a	-3.85	Sult1c2	-2.17	Ttr	-7.03
Slc22a4	-3.84	Adamts18	-3.78	Rassf10	-2.15	5430427M07Rik	-7.01
Pde6a	-3.79	Susd4	-3.76	Cyp2c68	-2.15	Pbld1	-6.92
Igfbp2	-3.78	Vip	-3.72	Per3	-2.12	Odf3b	-6.73
Ttr	-3.75	Pbld1	-3.70	Hoga1	-2.11	Afm	-6.67
Best2	-3.71	Cyp4f15	-3.69	Slc16a11	-2.09	Cyp2c55	-6.67
Pbld1	-3.70	Nmnat2	-3.69	Cbr3	-2.06	Ugt8a	-6.64
Odf3b	-3.59	Calb2	-3.56	Susd4	-2.06	Slc27a2	-6.58
